# Integrating genomics and metabolomics for scalable non-ribosomal peptide discovery

**DOI:** 10.1038/s41467-021-23502-4

**Published:** 2021-05-28

**Authors:** Bahar Behsaz, Edna Bode, Alexey Gurevich, Yan-Ni Shi, Florian Grundmann, Deepa Acharya, Andrés Mauricio Caraballo-Rodríguez, Amina Bouslimani, Morgan Panitchpakdi, Annabell Linck, Changhui Guan, Julia Oh, Pieter C. Dorrestein, Helge B. Bode, Pavel A. Pevzner, Hosein Mohimani

**Affiliations:** 1 Bioinformatics and Systems Biology Program, University of California San Diego, La Jolla, CA USA; 2 Center for Microbiome Innovation, University of California at San Diego, La Jolla, CA USA; 3 Computational Biology Department, School of Computer Science, Carnegie Mellon University, Pittsburgh, PA USA; 4 Molecular Biotechnology, Department of Biosciences, Goethe University Frankfurt, Frankfurt am Main, Germany; 5 Center for Algorithmic Biotechnology, Institute of Translational Biomedicine, St. Petersburg State University, St Petersburg, Russia; 6 Tiny Earth Chemistry Hub, University of Wisconsin–Madison, Madison, WI USA; 7 Collaborative Mass Spectrometry Innovation Center, Skaggs School of Pharmacy and Pharmaceutical Sciences, University of California San Diego, La Jolla, CA USA; 8 The Jackson Laboratory of Medical Genomics, Farmington, CT USA; 9 Buchmann Institute for Molecular Life Sciences (BMLS), Goethe University Frankfurt & Senckenberg Research Institute, Frankfurt am Main, Germany; 10 Max-Planck-Institute for Terrestrial Microbiology, Department for Natural Products in Organismic Interactions, Marburg, Germany; 11 Department of Computer Science and Engineering, University of California San Diego, La Jolla, CA USA

**Keywords:** Data mining, Software, Natural products

## Abstract

Non-Ribosomal Peptides (NRPs) represent a biomedically important class of natural products that include a multitude of antibiotics and other clinically used drugs. NRPs are not directly encoded in the genome but are instead produced by metabolic pathways encoded by *biosynthetic gene clusters* (BGCs). Since the existing genome mining tools predict many putative NRPs synthesized by a given BGC, it remains unclear which of these putative NRPs are correct and how to identify post-assembly modifications of amino acids in these NRPs in a blind mode, without knowing which modifications exist in the sample. To address this challenge, here we report NRPminer, a modification-tolerant tool for NRP discovery from large (meta)genomic and mass spectrometry datasets. We show that NRPminer is able to identify many NRPs from different environments, including four previously unreported NRP families from soil-associated microbes and NRPs from human microbiota. Furthermore, in this work we demonstrate the anti-parasitic activities and the structure of two of these NRP families using direct bioactivity screening and nuclear magnetic resonance spectrometry, illustrating the power of NRPminer for discovering bioactive NRPs.

## Abstract (web summary)

Current genome mining methods predict many putative non-ribosomal peptides (NRPs) from their corresponding biosynthetic gene clusters, but it remains unclear which of those exist in nature and how to identify their post-assembly modifications. Here, the authors develop NRPminer, a modification-tolerant tool for the discovery of NRPs from large genomic and mass spectrometry datasets, and use it to find 180 NRPs from different environments.

## Introduction

Microbial natural products represent a major source of bioactive compounds for drug discovery^1^. Among these molecules, non-ribosomal peptides (NRPs) represent a diverse class of natural products that include antibiotics, immunosuppressants, anticancer agents, toxins, siderophores, pigments, and cytostatics^1–4^. NRPs have been reported in various habitats, from marine environments^5^ to soil^3^ and even human microbiome^6–9^. However, the discovery of NRPs remains a slow and laborious process because NRPs are not directly encoded in the genome and are instead assembled by non-ribosomal peptide synthetases (NRPSs).

NRPSs are multi-modular proteins that are encoded by a set of chromosomally adjacent genes called biosynthetic gene clusters (BGCs)^10,11^. Each NRP-producing BGC encodes for one or more genes composed of NRPS modules. Together the NRPS modules synthesize the core NRP in an assembly line fashion, with each module responsible for adding one amino acid to the growing NRP. Each NRPS module contains an Adenylation domain (A-domain) that is responsible for recognition and activation of the specific amino acid^12^ that can be incorporated by that module through the non-ribosomal code^10^ (as opposed to the genetic code). At minimum, each NRPS module also includes a Thiolation domain (T-domain) and a Condensation domain (C-domain) that are responsible for loading and elongation of the NRP scaffold, respectively. Additionally, an NRPS module may include additional domains such as Epimerization domain (E-domain) or dual-function Condensation/Epimerization domain (C/E domain). An “NRPS assembly line” refers to a sequence of NRPS modules that together assemble a core NRP. The core NRP often undergoes post-assembly modifications (PAMs) that transform it into a mature NRP. The order of the modules in an NRPS assembly line can be different from the order of NRPS modules encoded in the BGC through iterative use of NRPS modules^13,14^.

In the past decade, genome mining methods have been developed for predicting the NRP sequences from their BGC sequences^15,16^. Genome mining tools, such as antiSMASH^17^, start by identifying the NRPS BGCs in a microbial genome using Hidden Markov Models (HMMs). Afterwards, they identify NRPS modules and predict the amino acids incorporated by the A-domain in each module using the substrate prediction algorithms (such as NRPSpredictor2 (ref. ^15^) or SANDPUMA^18^) that are based on machine learning techniques trained on a set of A-domains with known specificities^16,18^. For each observed A-domain, these algorithms predict a set of amino acids potentially recruited by that A-domain, along with the specificity score reflecting confidence of each amino acid prediction. The use of genome mining is becoming increasingly popular for discovering NRPs over the past decade^19–21^, demonstrating the potential of large-scale (meta)genomic projects for NRP discovery.

Although genome mining tools like SMURF^22^ and antiSMASH^17^ greatly facilitate BGC analysis, the core NRPs (let alone mature NRPs) for the vast majority of sequenced NRP-producing BGCs (>99%) remain unknown^23,24^. Identification of NRP-producing BGCs, without revealing the final molecular products they encode, does not capture its full potential for finding bioactive compounds^25^. Thus, integrating (meta)genome mining with metabolomics is necessary for realizing the true promise of large-scale NRP discovery^4^. However, the existing genome mining strategies fail to reveal the chemical diversity of NRPs. For example, these methods fall short in correctly identifying PAMs, which are a unique feature of NRPs that make them the most diverse class of natural products^26^ and play a crucial role in their mode of action^27,28^. As a result, the promise of large-scale NRP discovery has not yet been realized^29^.

Discovery of NRPs involves a multitude of challenges such as PAM identification (with exception of methylation and epimerization^17^, genome mining tools fail to identify PAMs) and accounting for substrate promiscuity of A-domains. The substrate promiscuity in NRP biosynthesis refers to the ability of an A-domain in an NRPS to incorporate several different amino acids into the NRP. The existing genome mining tools often predict a set of incorporated amino acids and output a ranked list of multiple amino acids for each A-domain. Allowing for all amino acid possibilities for each A-domain in an NRPS module results in a large number of putative NRPs predicted from each BGC. Without additional complementary data (such as mass spectra of NRPs), the genome mining approaches cannot identify the correct NRP among the multitude of putative NRPs^29,30^.

Another challenge in discovering NRPs is due to the non-canonical assembly lines. While in many NRPSs each A-domain incorporates exactly one designated amino acid and the sequence of amino acids in NRP matches the order of the A-domains in the BGC^13,31,32^ (see Supplementary Fig. 1a), there are many NRP families that violate this pattern^7,11,32–39^. Since an NRPS system may have multiple assembly lines^40^, one needs to consider different combinations of NRPS units encoded by each open reading frames (ORFs) for finding the core NRPs^27,40^. In some non-canonical assembly lines, A-domains encoded by at least one ORF may be incorporated multiple times (in tandem) in the NRPS^7,34–36^ (Supplementary Fig. 1b). For example, during biosynthesis of rhabdopeptides^34,38^ and lugdunins^7^, a single ORF encodes for one Val-specific NRPS module that loads multiple Val in the final NRPs. Moreover, in some NRPS assembly lines, the A-domains in some ORFs do not contribute to the core NRP^32,37,41^ (see Supplementary Fig. 1c). For example, surugamide BGC^30,32,42,43^ from *Streptomyces albus* produces two completely distinct NRPs through different non-canonical assembly lines (Supplementary Fig. 2). The non-canonical biosynthesis of surugamide makes its discovery particularly difficult as one need to account for these non-canonical assembly lines by generating different combinations of ORFs in the process of building a database of putative NRPs for each BGC.

Other hurdles include lack of sufficient training data for many A-domains, which can lead to specificity mispredictions^18^ and complications in the genome mining due to fragmented assemblies (e.g. failure to capture a BGC in a single contig^44^). These challenges, in combination with those mentioned above, make it nearly impossible to accurately predict NRPs based solely on genome mining. The problem gets even more severe for NRP discovery from microbial communities.

To address these challenges, multiple peptidogenomics approaches have been developed for discovering peptidic natural products by combining genome mining and mass spectrometry (MS) information^30,45^. These approaches often use antiSMASH^16^ to find all NRPS BGCs in the input genome, use NRPSPredictor2 (ref. ^15^) to generate putative core NRPs encoded by each BGC, and attempt to match mass spectra against these putative NRPs. Kersten et al.^44^ described a peptidogenomics approach based on manually inferring amino acid sequence tags (that represent a partial sequence of an NRP) from mass spectra and matching these tags against information about the substrate specificity generated by NRPSpredictor2 (ref. ^15^). Nguyen et al.^46,47^ and Tobias et al.^31^ presented a manual approach for combining genome mining with molecular networking. In this approach, which is limited to the identification of previously unreported variants of known NRPs, molecules present in spectral families with known compounds are compared to BGCs.

Medema et al.^40^ complemented the manual approach from Kersten et al.^44^ by the NRP2Path^40^ tool for searching the sequence tags against a collection of BGCs. NRP2Path starts with a set of sequence tags manually generated for each spectrum, considers multiple assembly lines for each identified BGC, and forms a database of all possible core NRPs for this BGC. Then, NRP2Path^40^ computes a match score between each tag and each core NRP (using the specificity scores provided by NRPSpredictor2 (ref. ^15^)) and reports high-scoring matches as putative core NRPs. The success of this approach relies on inferring long sequence tags of 4–5 amino acids, which are usually absent in spectra of non-linear peptides. Such long sequence tags are often missing in NRPs with macrocyclic backbones and complex modifications, limiting the applicability of NRP2Path^44,48^. Moreover, NRP2Path is not able to identify enzymatic modifications (e.g. methylation) and PAMs in the final NRPs and is unable to predict the backbone structure of the mature NRPs (e.g. linear/cyclic/branch-cyclic).

Mohimani et al.^30^ developed an automated NRPquest approach that takes paired MS and genomic datasets as input and searches each mass spectrum against all structures generated from putative core NRPs to identify high-scoring peptide-spectrum matches (PSMs). NRPquest leverages the entire mass spectrum (instead of just the sequence tags) to provide further insights into the final structure of the identified NRPs. They proposed using modification-tolerant search of spectral datasets against the core NRPs structures, for identifying PAMs in a blind mode (that is without knowing which PAMs exist in the sample). This is similar to identifying post-translational modifications in traditional proteomics^49^. The presence of covalent modifications in peptides affects the molecular weight of the modified amino acids; therefore, the mass increment or deficit can be detected using MS data^43,49^. However, as NRPquest uses a naïve pairwise scoring of all NRP structures against all mass spectra for PAM identification, it is prohibitively slow when searching for PAMs^30^. Furthermore, NRPquest does not handle non-canonical NRPS assembly lines and it does not provide statistical significance of identified NRPs, a crucial step for large-scale analysis.

On the other hand, development of high-throughput MS-based experimental and computational natural products discovery pipelines^29^ such as the Global Natural Products Social (GNPS) molecular networking^50^, PRISM^51^, GNP^52^, RODEO^53^, Dereplicator+^54^, CSI:FingerID^55^, NAP^56^, and CycloNovo^48^ have permanently changed the field of peptide natural product discovery. The GNPS project has already generated nearly half a billion of information-rich tandem mass spectra (MS), an untapped resource for discovering bioactive molecules. However, the utility of the GNPS network is mainly limited to the identification of previously discovered molecules and their analogs. Currently, only about 5% of the GNPS spectra are annotated^50^, emphasizing the need for algorithms that can annotate such large spectral datasets.

**Fig. 1:**
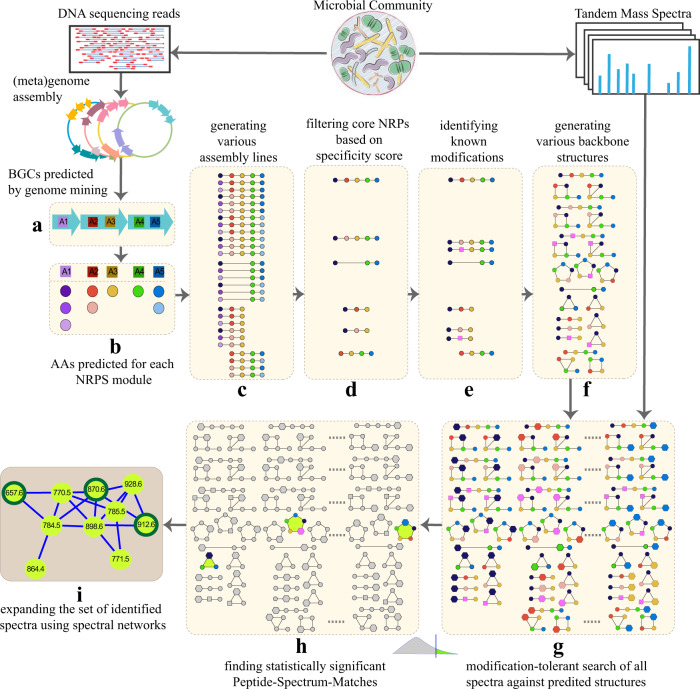
NRPminer pipeline. **a** Predicting NRPS BGCs using antiSMASH^16^. Each ORF is represented by an arrow, and each A-domain is represented by a square, **b** predicting putative amino acids for each NRP residue using NRPSpredictor2 (ref. ^15^), colored circles represents different amino acids (AAs), **c** generating multiple assembly lines by considering various combinations of ORFs and generating all putative core NRPs for each assembly line in the identified BGC (for brevity only assembly lines generated by deleting a single NRPS unit are shown; in practice, NRPminer considers loss of up to two NRPS units, as well as single and double duplication of each NRPS unit), **d** filtering the core NRPs based on their specificity scores, **e** identifying domains corresponding to known modifications and incorporating them in the selected core NRPs (modified amino acids are represented by purple squares), **f** generating linear, cyclic and branch-cyclic backbone structures for each core NRP, **g** generating a set of high-scoring PSMs using modification-tolerant VarQuest^43^ search of spectra against the database of the constructed putative NRP structures. NRPminer considers all possible mature NRPs with up to one PAM (shown as hexagons) in each NRP structure. For brevity some of the structures are not shown. **h** Computing statistical significance of PSMs and reporting the significant PSMs, and **i** expanding the set of identified spectra using spectral networks^57^. Nodes in the spectral network represent spectra and edges connect “similar” spectra (see “Methods”).

In this work, we present NRPminer a scalable modification-tolerant tool for analyzing paired MS and (meta)genomic datasets (Fig. 1). NRPminer uses the specificity scores of the amino acids appearing in core NRPs to perform an efficient search of all spectra against all core NRPs. In addition to predicting the amino acid sequence of an NRP generated by a BGC, NRPminer also analyzes various non-canonical assembly lines and efficiently predicts potential PAMs and backbone structures. We show NRPminer identifies 180 unique NRPs representing 18 distinct NRP families, including four previously unreported ones, by analyzing only four MS datasets in GNPS against their corresponding reference genomes.

## Results

### Outline of the NRPminer algorithm

Figure 1 illustrates the NRPminer algorithm. All NRPminer’s steps are described in detail in the “Methods” section. Briefly, NRPminer starts by (a) identifying the NRPS BGCs in each genome (using antiSMASH^16^) and (b) predicting the putative amino acids for each identified A-domain (using NRPSpredictor2 (ref. ^15^)). Then, it accounts for (c) different NRPS assembly lines by considering various combinations of ORFs in the BGCs. NRPminer (d) filters the set of all core NRPs based on the specificity scores of their amino acids and selects those with high scores. It, next, (e) searches each BGC to find known modification enzymes (e.g. methylation) and incorporates them in the corresponding core NRPs. Then, (f) NRPminer constructs a database of putative NRP structures by considering linear, cyclic, and branch-cyclic backbone structures for each core NRP. Afterwards, (g) it performs a modification-tolerant search of the input spectra against the constructed database of putative NRPs and computes the statistical significance of PSMs. Finally, (h) NRPminer reports the statistically significant PSMs. These identifications are then (i) expanded using spectral networks^57^ approach.

### Datasets

We analyzed four microbial isolate datasets from *Xenorhabdus* and *Photorhabdus* families (XPF), *Staphylococcus* (SkinStaph)*,* soil-dwelling Actinobacteria (SoilActi), and a collection of soil-associated bacteria within *Bacillus*, *Pseudomonas*, *Buttiauxella*, and *Rahnella* genera generated under the Tiny Earth antibiotic discovery project^58,59^ (TinyEarth); all available from GNPS/MassIVE repository. The process of growth of the isolates and MS experiments are described in the “Methods” section (under “Sample preparation and MS experiments). The spectra collected on each of these datasets are referred to as spectra_XPF_, spectra_SkinStaph_, spectra_SoilActi_, spectra_TinyEarth_, and the genomes are referred as genome_XPF_, genome_SkinStaph_, genome_SoilActi_, and genome_TinyEarth_, respectively.

### Summary of NRPminer results

Table 1 summarizes the NRPminer results for each dataset. NRPminer classifies a PSM as statistically significant if its *p* value is below the default conservative threshold 10^−15^. The number of distinct NRPs and NRP families was estimated using MS-Cluster^60^ and SpecNets^50^ using the threshold cos < 0.7 (see “Methods” section). Two peptides are considered to be variants/modifications of each other if they differ in a single modified residue due to changes by tailoring enzymes, enzyme promiscuity, or through changes in the amino acid specificity at the genetic level^47^. Known NRPs (NRP families) are identified either by Dereplicator^42^ search against the database of all known peptidic natural products^43^ (referred to as PNPdatabase) using the *p* value threshold 10^−15^, and/or by SpecNet^57^ search against the library of all annotated spectra available on GNPS^50^. NRPminer ignores any BGCs with less than three A-domains and spectra that include less than 20 peaks.

### Generating putative core NRPs

Table 1 presents the number of NRP-producing BGCs and the number of putative core NRPs generated by NRPminer for each analyzed genome in XPF (before and after filtering). For example, NRPminer identified eight NRP-producing BGCs and generated 253,027,076,774 putative core NRPs for *X. szentirmaii* DSM genome. After filtering putative core NRPs based on the sum of the specificity scores reported by NRPSpredictor2 (ref. ^15^), only 29,957 putative core NRPs were retained (see “Methods” section for the details of filtering). Therefore, filtering putative core NRPs is an essential step for making the search feasible.

**Table 1:** Summary of NRPminer search results on the XPF, SkinStaph, SoilActi, and TinyEarth datasets.

Dataset	#strains	#identified PSMs/#spectra	#distinct NRPs (families)	#known NRPs (families)	#preiviously unreported variants of known NRPs	#previuosly unreported NRPs (families)
XPF	27	3023/263,768	122 (12)	21 (9)	79	22 (3)
SkinStaph	171	23/2,657,398	3 (1)	2 (1)	1	0
SoilActi	20	206/362,421	24 (2)	7 (1)	14	3 (1)
TinyEarth	28	498/380,414	31 (3)	29 (3)	2	0

Column “#strains” shows the number of microbial strains. Column “#identified PSMs/#spectra” shows the number of PSMs identified by NRPminer and the total number of spectra. The column “#distinct NRPs (families)” shows the number of unique NRPs (unique families). The number of unique NRPs is estimated using MS-Cluster60, and the number of unique families is estimated using SpecNets50. The column “#known NRPs (families)” shows the number of known NRPs (families) among all identified NRPs (families). Column “#previously unreported variants of known NRPs” shows the number of NRPs in the known families that were not reported before. Column “#previously unreported NRPs (families)” shows the number of previously unreported NRPs (families) that are not variants of any known NRPs.

### Analysis of the paired genomic and spectral datasets

NRPminer has a one-vs-one mode (each MS dataset is searched against a single genomic dataset) and a one-vs-all mode (each MS dataset is searched against a collection of genomic datasets within a taxonomic clade). While the one-vs-all mode is slower than the one-vs-one mode, it is usually more sensitive. For example, a BGC may be fragmented (or misassembled) in the draft assembly of one strain, but a related BGC may be correctly assembled and captured within a single contig in a related well-assembled strain. If these two BGCs synthesize the same (or even similar) NRP, NRPminer may be able to match the spectra from a poorly assembled strain to a BGC from a related well-assembled strain.

**Fig. 2:**
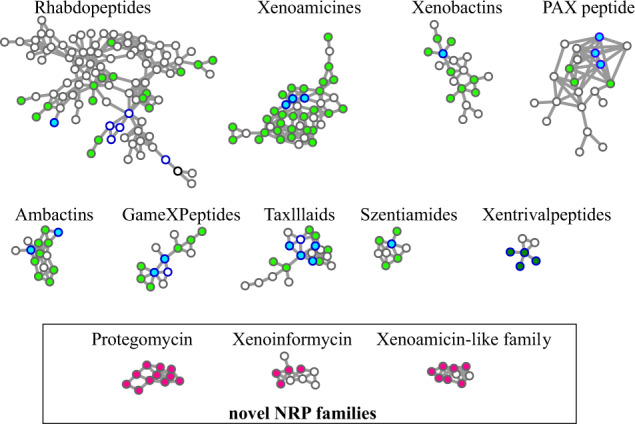
Spectral networks for nine known and three previously unreported NRP families identified by NRPminer in the XPF dataset. Each node represents a spectrum. The spectra of known NRPs (as identified by spectral library search against the library of all known compounds in GNPS) are shown with a dark blue border. A node is colored if the corresponding spectrum forms a statistically significant PSM and not colored otherwise. We distinguish between identified spectra of known NRPs with known BGCs^31^ (colored by light blue) and identified spectra of known NRPs (from xentrivalpeptide family) with previously unknown BGC (colored by dark green). Identified spectra of previously unreported NRPs from known NRP families (previously unreported NRP variants) are colored in light green. Identified spectra of NRPs from previously unreported NRP families are colored in magenta. Proteogomycins and xenoinformycin subnetworks represent previously unreported NRP families. The Xenoamicin-like subnetwork revealed a BGC family distantly related to xenoamicins (6 out 13 amino acids are identical). For simplicity only spectra at charge state +1 are used for the analysis.

For example, NRPminer search of spectra_XPF_ against genome_XPF_ generated 3023 PSMs that represent 122 NRPs from 12 NRP families. Figure 2 shows the spectral network representing 12 NRP families identified by NRPminer in the XPF dataset. SpecNet analysis against the annotated spectra in GNPS^50^ showed that 9 out of 12 identified NRP families is known (reported by Tobias et al.^31^). NRPminer identified PAX-peptides family and their corresponding BGC in *X. nematophila* ATCC 19061 in the XPF dataset even though these NRPs include lipid side chains that are not predictable via genome mining. NRPminer failed to identify only one additional known family which was reported by Tobias et al.^31^ (xefoampeptides) that has an ester bond between a hydroxy-fatty acid and the terminal amino acid with total mass exceeding the default NRPminer threshold (150 Da). Xefoampeptides are depsipeptides composed of a 3-hydroxy-fatty acid (total mass over 200 Da) and only three amino acids, resulting in a poorly fragmented spectrum that did not generate statistically significant PSMs against the putative structures generated from their corresponding core NRPs. Table 2 provides information about NRPminer-generated PSMs representing known NRP families. Among the nine known NRP families (in the XPF dataset) listed in Table 2, eight families have been connected to their BGCs in the previous studies, and for these families, the corresponding BGCs discovered by NRPminer are consistent with the literature^31^ (see Supplementary Table 2 for the list of identified BGCs). Supplementary Figure 3 presents an example of an identified NRP family, szentiamide, and its corresponding BGC in *X. szentirmaii*. For one family (xentrivalpeptides) with no known BGC, we were able to predict the putative BGC (Supplementary Fig. 4). Furthermore, NRPminer identified 79 previously unreported NRP variants across these nine known NRP families. In addition to the known NRP families, NRPminer also discovered three NRP families (protegomycins, xenoinformycins, and xenoamicin-like family) in XPF dataset that includes no previously reported NRPs.

**Table 2:** Predicted amino acids for the eight A-domains appearing on cyclic surugamides A–D assembly line SurugamideAL.

$A$_1_	$A$_2_	$A$_3_	$A$_4_	$A$_5_	$A$_6_	$A$_7_	$A$_8_
Val (100)	**Phe (100)**	Tyr (100)	Val (100)	**Ala (100)**	Val (100)	Val (100)	Met (100)
**Ile (80)**	Tyr (90)	Phe (100)	**Ile (100)**	Ser (87)	**Ile (100)**	**Ile (100)**	Apa (100)
Abu (70)	Bht (90)	**Leu (100)**	Abu (70)	Pro (75)	Abu (70)	Abu (70)	Glu (86)
				Val (75)			Arg (86)
				Cys (75)			Gln (86)
				Phe (75)			**Lys (86)**
				Gly (75)			Asp (86)
							Val (86)
							Orn (86)

*A*_*i*_ represents the set of amino acids predicted for the *i*th A-domain in ${\rm{SurugamideAL}}$. For each *A*_*i*_ at least three amino acids with the highest normalized specificity scores (listed in parentheses) are presented. Amino acids appearing in surugamide A (IFLIAIIK) are shown in bold. NRPminer considers all amino acids with the same normalized specificity score, as illustrated in the case of the fifth and the eighth A-domains.

We named each identified NRP in a previously unreported family by combining the name of that family with the nominal precursor mass of the spectrum representing that NRP (with the lowest *p* value among all spectra originating from the same NRP). In what follows, we describe the four previously unreported NRP families identified by NRPminer (protegomycin, xenoinformycin, and xenoamicin-like family in the XPF dataset and aminformatide in SoilActi), as well as the previously unreported variants in two additional NRP families (lugdunin in SkinStaph and surugamide in SoilActi).

### Discovery of protegomycin (PRT) NRP family in the XPF dataset

**Fig. 3:**
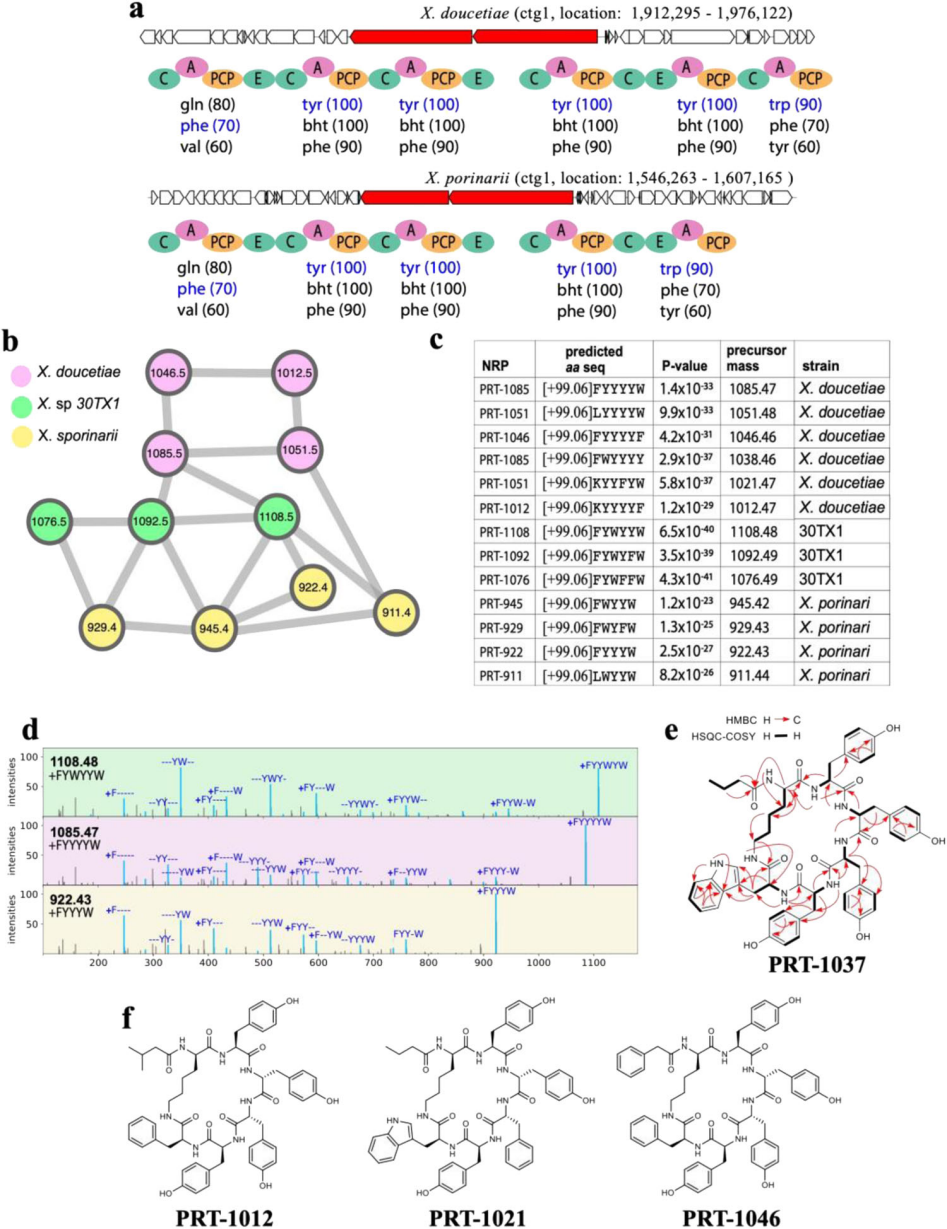
Identifying protegomycin (PRT) NRP family. **a** The BGCs generating the NRP in *X. doucetiae* (top) and *X. porinarii* (bottom) along with NRPS genes (shown in red) and A-, C-, PCP-, and E-domains in these NRPSs. The rest of the genes in the corresponding contigs is shown in white. No BGC was found in *Xenorhabdus* sp. 30TX1. Three highest-scoring amino acids for each A-domain in these BGCs (according to NRPSpredictor2 (ref. ^15^) predictions) are shown below the corresponding A-domains. Amino acids appearing in the NRPs [+99.06]FYYYYW and [+99.06]FYYYW identified by NRPminer (with the lowest *p* value) are shown in blue. **b** Spectral network formed by the spectra that originate from NRPs in the protegomycin family. **c** Sequences of the identified NRPs in the protegomycin family (with the lowest *p* value among all spectra originating from the same NRP). PRT represents protegomycin. For MS details see Supplementary Table 3. The *p* values are computed based on MCMC approach using MS-DPR^89^ with 10,000 simulations. **d** For each strain, an annotated spectrum representing the lowest *p* value is shown. The spectra were annotated based on predicted NRPs [+99.06]FYYWYW, [+99.06]FYYYYW, and [+99.06]FYYYW from top to bottom. The “+” sign represents the addition of [+99.06 Da]. Colors in parts **b** and **d** are coordinated. Supplementary Figures 6–8 show the annotated spectra for all NRPs shown in part (**c**). **e** Key HMBC and HSQC-COSY correlations in PRT-1037. **f** Structures for selected PRT derivatives produced by *X. doucetiae* including amino acid configuration as concluded from the presence of epimerization domains in the corresponding NRPSs and acyl residues as concluded from feeding experiments (Supplementary Fig. 9). Predicted structures for all identified PRT derivatives from *X. doucetiae*, *X. poinarii*, and 30TX1 are shown in Supplementary Figs. 10 and S11.

NRPminer matched 28 spectra representing 11 previously unreported cyclic NRPs to two BGCs. These spectra are from species *X. doucetiae*, *Xenorhabdus* sp. 30TX1, and *X. poinarii*. The BGCs were from *X. doucetiae* and *X. poinarii* with six and five A-domains, respectively, with one PAM (Fig. 3). Additional derivatives were found in large-scale cultivation of wild type and Δ*hfq* mutants of *X. doucetiae* (Supplementary Fig. 5 and “Methods” section under “Additional Analyses for Protegomycin Family”). No BGC was found in *Xenorhabdus* sp. 30TX1 due to highly fragmented assembly. The spectra representing the three protegomycins produced by *Xenorhabdus* sp. 30TX1 did not match any core NRP generated from its genome because the corresponding BGC was not assembled in a single contig in this genome. However, they were identified with statistically significant *p* values using the one-vs-all search when these spectra were searched against core NRPs from *X. doucetiae* genome (Fig. 3) that included an orthologous BGC in a single contig. Figure 3, Supplementary Figs. 6–11, and Supplementary Table 3 present information about protegomycin BGC and NRPs.

We further conducted nuclear magnetic resonance (NMR) spectroscopy on one of the major derivatives (Fig. 3e, f and Supplementary Figs. 12–18 and Supplementary Table 4). Our NMR results confirmed the MS results, with the distinction that NMR revealed a short chain fatty acid like phenylacetic acid (PAA) as a starting unit (incorporated by the C-starter domain), followed by a Lys that is cyclized to the terminal thioester by the C-terminal TE domain. NRPminer predicted Phe instead of the correct amino acid Lys, since NRPSpredictor2 made an error in identifying the amino acid for the corresponding A-domain (see Fig. 3a for the list of predicted amino acids). It has been shown that NRPSpredictor2 (ref. ^15^) often fails to predict Lys residues, due to lack of training data for this amino acid^15^. Furthermore, as with any other MS-based method, NRPminer was not able to distinguish between residues with the same molar mass in the structure of final NRP, such as the pair Ala and β-Ala. All other NRPminer predictions of individual amino acids were consistent with NMR.

Besides PAA, other starter acyl units are isovaleric acid (in PRT-1012; NRPminer prediction 99.06+Leu; see Fig. 3f) and butyric acid (in PRT-1037; see Fig. 3e). Supplementary Figure 9 describes labeling data and mass spectra for the identified protegomycins in *X. doucetiae*. The isolated derivatives PRT-1037 and PRT-1021 (Fig. 3e, f) were tested against various protozoa and showed a weak activity against *Trypanosoma brucei rhodesiense* (IC_50_ [mg/L] 79 and 53) and *Plasmodium falciparum* (IC_50_ [mg/L] > 50 and 33) with no toxicity against L6 rat myoblast cells (IC_50_ [mg/L] both >100).

### Discovery of xenoinformycin (XINF) NRP family in the XPF dataset

**Fig. 4:**
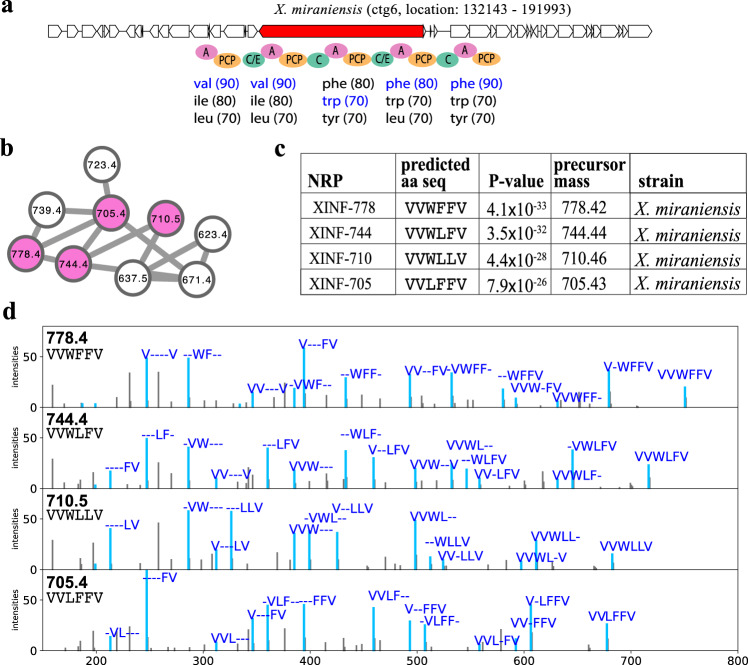
Identifying xenoinformycin (XINF) NRP family. **a** The BGC generating the NRP in *X. miraniensis* along with NRPS genes (shown in red) and the A-, C-, PCP-, and C/E-domains appearing on the corresponding NRPS. The rest of the genes in the corresponding contigs are shown in white. Three highest-scoring amino acids for each A-domain in this BGC (according to NRPSpredictor2 (ref. ^15^) predictions) are shown below the corresponding A-domains. Amino acids appearing in the NRP VVWFF identified by NRPminer (with the lowest *p* value) are shown in blue. **b** Spectral network formed by the spectra that originate from NRPs in the xenoinformycin family. A node is colored if the corresponding spectrum forms a statistically significant PSM (with *p* value threshold 10^−15^) and not colored otherwise. **c** Sequences of the identified NRPs in the xenoinformycin family (with the lowest *p* value among all spectra originating from the same NRP). XINF represents xenoinformycin. The *p* values are computed based on MCMC approach using MS-DPR^89^ with 10,000 simulations. **d** For each identified NRP, an annotated spectrum forming a PSM with the lowest *p* value is shown.

NRPminer matched four spectra representing four cyclic NRPs *X. miraniensis* dataset to a previously uncharacterized BGC in its genome (Fig. 4). NRPminer reported a modification with a total mass of 99.068 for all the four identified NRPs, which matches the valine mass. We hypothesize that one of the valine-specific adenylation domains is responsible for the activation of two consecutive valine units, suggesting an iterative use of the Val-incorporating module (similar to stuttering observed in polyketide synthases^61,62^) but this is yet to be experimentally verified. Interestingly, the predicted xenoinformycin producing NRPS XinfS is highly similar to the widespread NRPS GxpS found in *Xenorhabdus* and *Photorhabdus*, responsible for the GameXPeptide production^31,63^. While both XinfS and GxpS have five modules, XinfS has a C-domain instead of the usual C/E-domain in the last module, suggesting a different configuration of the amino acid Phe or Leu (corresponding to the second last A-domain on their NRPSs), respectively.

### Discovery of xenoamicin-like (XAM) NRP family in the XPF dataset

**Fig. 5:**
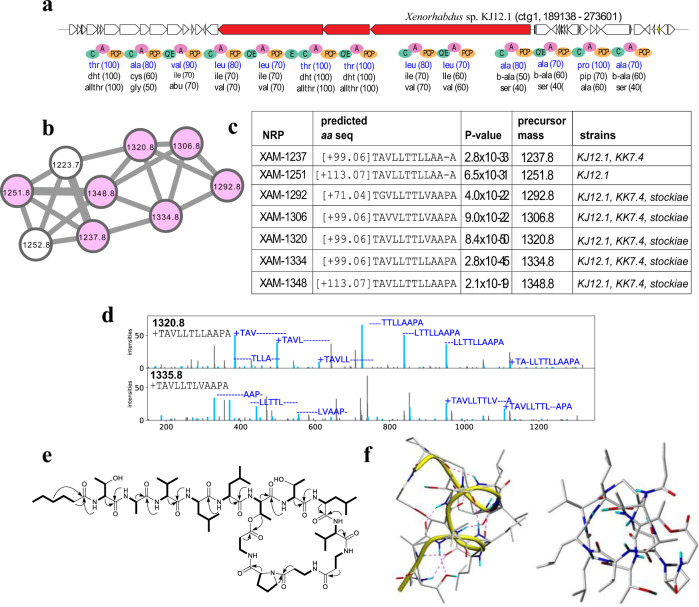
Identifying xenoamicin-like (XAM) NRP family. **a** The BGCs generating the NRP in *Xenorhabdus* sp. KJ12 along with NRPS genes (shown in red) and A-, C-, PCP-, and E-domains in these NRPSs. The rest of the genes in the corresponding contigs are shown in white. Three highest-scoring amino acids for each A-domain in these BGCs (according to NRPSpredictor2 (ref. ^15^) predictions) are shown below the corresponding A-domains. Amino acids appearing in the NRP [+99.06]TAVLLTTLLAAPA identified by NRPminer (with the lowest *p* value) are shown in blue. **b** Spectral network formed by the spectra that originate from NRPs in the XAM family. **c** Sequences of the identified NRPs in this family (with the lowest *p* value among all spectra originating from the same NRP). The *p* values are computed based on MCMC approach using MS-DPR^89^ with 10,000 simulations. **d** For each strain, an annotated spectrum representing the lowest *p* value is shown. The spectra were annotated based on predicted NRPs [+99.06]TAVLLTTLLAAPA and [+99.06] TAVLLTTLVAAPA from top to bottom. The “+” sign represents the addition of [+99.06]. Supplementary Figures 23 and S24 show the annotated spectra for the other NRPs shown in part (**c**). **e** NMR-based correlations of XAM-1320 (*m*/*z* 1320.8 [M+H]^+^) produced by *Xenorhabdus* KJ12.1 (Supplementary Table 5 and Supplementary Figs. 25–29). HSQC-TOCSY (bold lines) and key ROESY correlations (arrows) are shown. **f** 3D structure of XAM-1320 derived from 121 ROE-derived distance constraints (Supplementary Table 6), molecular dynamics, and energy minimization. Peptide backbone is visualized with a yellow bar (left). Predicted hydrogen bonds stabilizing the β-helix are shown as dashed lines. View from above at the pore formed by XAM-1320 (right). NRPminer identified this NRP with *p* value 8.4 × 10^−50^.

NRPminer discovered an NRP family that includes eight distinct NRPs, along with their BGC (Fig. 5). While the matched BGC for this family is evolutionary related to the xenoamicin BGC^64^ and both BGCs include 13 A-domains, 7 out of 13 amino acids in XAM differ from the corresponding amino acids in xenoamicin A (Supplementary Fig. 19). We named this previously unreported class of xenoamicins class III. Interestingly, the occurrence of XAM-1237 and XAM-1251 suggest a loss of Pro in their structure indicating another possibility of NRP diversification, namely module skipping as previously observed in other NRPSs^61,65,66^. We confirmed the sum formula of XAM-1320 (*m*/*z* 1320.793 [M + H^+^]; C_63_H_109_N_13_O_17_) and XAM-1334 (*m*/*z* 1334.810 [M + H^+^]; C_64_H_111_N_13_O_17_) by feeding (Supplementary Figs. 20 and 21) and MS–MS experiments (Supplementary Fig. 22 and Methods section under “Additional analysis for xenoamicin-like family”) and were also able to isolate the major derivative XAM-1320 from *Xenorhabdus* sp. KJ12.1 and to elucidate its structure by NMR including its 3D solution structure (Supplementary Tables 5 and 6 and Supplementary Figs. 25–S29) that confirms its β-helical structure from the alternating D/L configurations (confirmed by the advanced Marfey’s analysis; Supplementary Fig. 23 and “Methods” section) throughout the peptide chain from the presence of C/E domains, except for the C-terminal part shown in Fig. 5. XAM-1320 was also tested against protozoa and showed a good activity against *T. brucei rhodesiense* (IC_50_ [mg/L] 3.9) but much lower activity against *Trypanosoma cruzi*, *Plasmodium falciparum* and rat L6 cells (IC_50_ [mg/L] 25.5, 56.2, and 46.0, respectively). Supplementary Figure 24 provides information about the isolation and structure elucidation of XAM-1320, XAM-1278, XAM-1292, and XAM-1348 that differed in the starter acyl unit and the following amino acid (Ala or Gly).

### Discovery of aminformatide NRP family produced by *Amycolatopsis* sp. aa4 in the SoilActi dataset

**Fig. 6:**
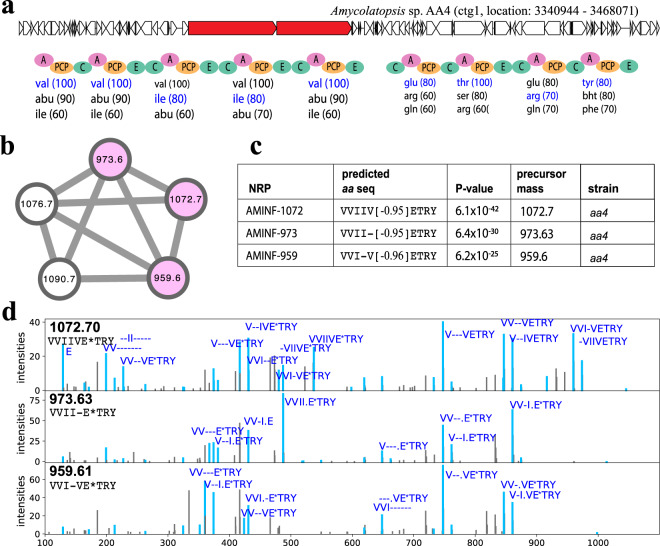
Identifying aminformatide (AMINF) NRP family discovered by NRPminer in the *SoilActi* dataset. **a** The BGC generating the core NRP in *Amycolatopsis* sp. AA4 along with NRPS genes (shown in red) and the A-, C-, PCP, and E-domains appearing in the corresponding NRPS. The rest of the genes in the corresponding contigs are shown in white. Three highest-scoring amino acids for each A-domain in this BGC (according to NRPSpredictor2 (ref. ^15^) predictions) are shown below the corresponding A-domains. Amino acids appearing in the NRP VVIVETRY identified by NRPminer (with the lowest *p* value) are shown in blue. **b** Spectral network formed by spectra that originate from the AMINF NRPs. A node is colored if the corresponding spectrum forms a statistically significant PSM and not colored otherwise. The *p* values are computed based on MCMC approach using MS-DPR^89^ with 10,000 simulations. **c** Sequences of the NRPs identified by NRPminer in the aminformatide family (with the lowest *p* value among all PSMs originating from the same NRP). NRPminer predicted a PAM with loss of ~0.96 Da on E, represented by E*. AMINF represents aminformatide. **d** For each identified NRP, an annotated spectrum representing the lowest *p* value is shown.

Supplementary Table 7 presents the number of NRP-producing BGCs and the number of putative core NRPs generated by NRPminer for each analyzed genome in XPF (before and after filtering). NRPminer identified 11 PSMs (representing three NRPs) when searching the SoilActi spectral dataset against *Amycolatopsis* sp. aa4 genome (Fig. 6). Previously, another NRP family, siderophore amychelin, and its corresponding BGC was reported from this organism^67^. Using the NRPSpreidctor2 (ref. ^15^)-predicted amino acids NRPminer predicted a modification of ~0.95 Da on the Glu in aminoformatide-1072 VVII[E-1.0]TRY. Since NRPSpredictor2 is the least sensitive in recognizing Lys (as compared to other amino acids)^15^, we hypothesize that this amino acid is in fact a Lys as we have seen in the case of protegomycins (with Lys), but this is yet to be determined.

### Identifying lugdunin NRP family in the SkinStaph dataset

**Fig. 7:**
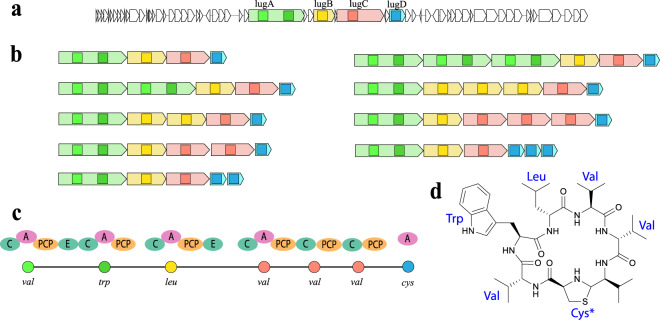
Lugdunin BGC and the assembly lines formed by NRPminer using the OrfDup option. **a** Lugdunin BGC with the four ORFs shown in different colors. The squares represent the A-domains. **b** Assembly lines formed by duplication of a single NRPS subunit (corresponding to each ORF) zero, one, and two times are pictured. NRPminer explores all assembly lines generated by duplicating each ORF up to two times when the “OrfDup” option is selected. **c** The NRPS assembly lined (with A-, C-, PCP-, and E-domains pictured) appearing in the NRPS that synthesizes lugdunin, where one Val-specific A-domain loads three amino acids (*valines*) to the growing peptide. Amino acids corresponding to lugdunin structure are shown below each A-domain. Circles represent amino acids (different amino acids are shown by different colors). **d** Cyclic structure of lugdunin with the amino acids highlighted in blue. The “Cys*” represent Cys-derived thiazolidine in lugdunin structure.

Antibiotics lugdunins^7^ represent the only NRP family reported in the human commensal microbiota. NRPminer matched nine spectra representing three NRPs from a single family in the spectra_SkinStaph_ dataset against *Staphylococcus lugdunensin* genome. In addition to the two known cyclic variants of lugdunin, NRPminer also discovered a previously unreported lugdunin variant with precursor mass 801.52 (Supplementary Fig. 30). Due to a +18.01 Da mass difference, NRPminer predicted a linear structure for this variant that represents the linear version of the known one. Since NRPminer predicts sequence VWLVVVt for the linear lugdunin, with the breakage between valine and Cys-derived thiazolidine, we hypothesize that this is a naturally occurring linear derivative in the lugdunin family. Lugdunins, synthesized by a non-canonical assembly line, were predicted using the non-canonical assembly line feature of NRPminer (Fig. 7).

### Identifying lipopeptides in the TinyEarth dataset

**Fig. 8:**
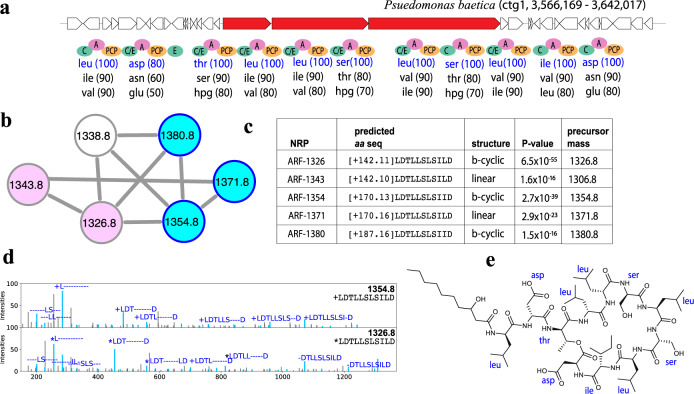
Arthrofactin (ARF) NRP family. **a** The BGCs generating the NRP in *Pseudomonas baetica* sp. 04-6(1) along with the NRPS genes (shown in red) and A-, C-, C/E-, PCP-, and E-domains in these NRPSs. The rest of the genes in the corresponding contigs are shown in white. Three highest-scoring amino acids for each A-domain in these BGCs (according to NRPSpredictor2 (ref. ^15^) predictions) are shown below the corresponding A-domains. Amino acids appearing in the known NRP ARF-1354 with amino acid sequence [+170.13]LDTLLSLSILD are shown in blue. **b** Spectral network formed by the spectra that originate from NRPs in the ARF family. The known arthrofactins are shown in blue, while the purples nodes represent the previously unreported variants identified by NRPminer. All identified athrofactins share the same core NRP LDTLLSLSILD. **c** Sequences of the identified NRPs in this family (with the lowest *p* value among all spectra originating from the same NRP). Column “structure” shows if the predicted structure for the identified NRPs is linear or branch-cyclic (shown by b-cyclic). The *p* values are computed based on MCMC approach using MS-DPR^89^ with 10,000 simulations. **d** Two annotated spectra representing the PSMs (with the lowest *p* values among spectra originating from the same NRPs) corresponding to ARF-1354 and 1326. The two spectra were annotated based on predicted NRPs [+170.13]LDTLLSLSILD (PSM *p* value 2.7 × 10^−39^) and [+142.11]LDTLLSLSILD (PSM *p* value 6.5 $\times$ 10^−55^), from top to bottom. The “+” and “*” signs represent the addition of [+170.13] and [+142.11], respectively. **e** The 2D structure of known arthrofactin ARF-1354 (ref. ^80^). NRPminer identified this NRP with *p* value 2.7 × 10^−39^.

Our NRPminer analysis of the TinyEarth dataset generated 498 PSMs representing 31 NRPs from three families, using the 200 Da threshold for PAM identification. Supplementary Table 9 provides information about the NRPminer-generated PSMs representing these three NRP families. *Bacillus* derived surfactins^68^ and plipastatin^69^ are bioactive lipopeptide with wide variety of activities. Surfactins are reported to have anti-viral^70,71^, anti-tumor^72^, anti-fungal^73^, and anti-microbial^74^ functions^75–78^ and plipastatins have known anti-fungal activities^79^. In the analysis of *Bacillus amyloliquefaciens* sp. GZYCT-4-2, NRPminer correctly reported all known surfactins (17 NRPs) and plipastatins (9 NRPs) identified in this dataset (PSMs listed in Supplementary Table 10). Moreover, NRPminer search of spectra_TinyEarth_ against putative NRP structures generated from *Pseudomonas baetica* sp. 04-6(1) genome identified 63 PSMs representing the arthrofactins (ARF) NRP family (Fig. 8). NRPminer identified the known branch-cyclic arthrofactins^80^ that only differ in the fatty acid tail (namely ARF-1354 and ARF-1380) and a known linear arthrofactin ARF-1372 (the linear version of ARF-1354). Furthermore, it identified two previously unreported arthrofactin variants: ARF-1326 (predicted to only differ in its side chain from the known branch-cyclic ARF-1354 shown in Fig. 8e) and ARF-1343 (predicted to be the linear version of the putative ARF-1326). NRPminer missed one known NRP family identified in spectra_TinyEarth_ (xantholysins^81^) since the xantholysin BGC was split among multiple contigs in the *Pseudomonas plecoglossicida* sp. YNA158 genome assembly.

### Identifying surugamides in the SoilActi dataset

NRPminer identified 183 spectra representing 25 NRPs when searching spectra_SoilActi_ against *S. albus* J10174 genome, hence extending the set of known surugamide variants from 8 to 21 (Supplementary Table 8 and Supplementary Fig. 2). Spectral network analysis revealed that these spectra originated from two NRP families. VarQuest search of this spectral dataset against PNPdatabase^43^ identified only 14 of these 21 NRPs. The remarkable diversity of surugamide NRPs, which range in length from 5 to 10 amino acids, is explained by the non-canonical assembly lines^13,43^. Using the “orfDel” option when analyzing surugamide BGC, with four ORFs (see Fig. S31), NRPminer generated 11 assembly lines. Supplementary Table 12 presents the number of core NRPs generated from the assembly line formed by SurA and SurD genes, based on their scores; 1104 core NRPs are retained out of 45,927 possible core NRPs generated from this assembly line. In total, 14,345 core NRPs from the original 3,927,949,830 core NRPs of the 11 assembly lines of surugamide BGC are retained. In addition to the surugamides synthesized by the SurA-SurD pair, NRPminer also discovered Surugamide G synthesized by the SurB-SurC pair (Supplementary Fig. 2d). In comparison with surugamide F from *Streptomyces albus*^32^, this NPR lacks the N-terminal tryptophan. Surugamide F was not identified in the spectral dataset from *Streptomyces albus*.

## Discussion

We developed the scalable and modification-tolerant NRPminer tool for automated NRP discovery by integrating genomics and metabolomics data. We used NRPminer to match multiple publicly available spectral datasets against 241 genomes from RefSeq^82^ and genome online database (GOLD)^83^. NRPminer identified 55 known NRPs (13 families) whose BGCs have been identified previously, without having any prior knowledge of them (Figs. 2 and 7, Supplementary Fig. 2, S3, and S25, and Supplementary Table 2 and S8). Furthermore, NRPminer identified the BGC for an orphan NRP family (xentrivalpeptides) with previously unknown BGC. In addition to the known NRPs, NRPminer reported 121 previously unreported NRPs from a diverse set of microbial organisms. Remarkably, NRPminer identified four NRP families, representing 25 previously unreported NRPs with no known variants, three families in the XPF dataset (Figs. 3–5) and one in the SoilActi dataset (Fig. 6), illustrating that it can match large spectral datasets against multiple bacterial genomes for discovering NRPs that evaded identification using previous methods. We further validated two of the previously unreported families predicted by NRPminer using NMR and demonstrated their anti-parasite activities.

Existing peptidogenomics approaches are too slow (and often memory-intensive) to conduct searches of large MS datasets against many genomes. Moreover, these approaches are limited to NRPs synthesized by canonical assembly lines and without PAMs, which limits the power of these methods for discovering NRPs. NRPminer is the first peptidogenomics tool that efficiently filters core NRPs based on their specificity scores without losing sensitivity and enables searching millions of spectra against thousands of microbial genomes. Furthermore, NRPminer can identify NRPs with non-canonical assembly lines of different types (e.g., surugamides, xenoinformycin, and lugdunin) and PAMs (e.g. surfactins, arthrofactins, plipastatins, protegomycins, and PAX peptides).

Majority of the spectral datasets in GNPS are currently not accompanied by genomics/metagenomics data. To address this limitation, NRPminer can search a spectral dataset against all genomes from RefSeq^82^ or GOLD databases^83^ within a user-defined taxonomic clade. This one-vs-all mode enables analysis of spectral datasets that are not paired with genomic/metagenomic data by searching them against multiple genomes. This mode, which relies on the scalability of NRPminer, enabled NRPminer to identify the lugdunin family (by searching the SkinStaph spectral dataset) even though the paired genome sequence from the same strain was not available.

In contrast to the previous peptidogenomics approaches, NRPminer is robust against errors in specificity prediction in genome mining tools and can efficiently identify mature NRPs with PAMs. This feature was crucial for discovering protegomycins that include a PAM (lipid chain) and a mis-prediction (Phe instead of Lys), as well as for identifying the lipopeptide biosurfactant in the TinyEarth dataset. While NRPminer is a powerful tool for discovering NRPs it can only succeed if the genome mining algorithms successfully identify an NRP-encoding BGC and predict the correct amino acids for nearly all A-domains. One of the bottlenecks of genome mining methods for NRP discovery is the lack of training data for many non-standard amino acids from under-explored taxonomic clades. We anticipate that more NRPs will be discovered using automated methods, and these discoveries will increase the number of A-domain with known specificity, which in turn will pave the path toward the development of more accurate machine learning techniques for A-domains specificity prediction.

In case of metagenomic datasets, NRPminer’s one-vs-all function allows for searching the spectral dataset against all the metagenomic assemblies generated from the same sample. However, the success of genome mining crucially depends on capturing the entire BGCs in a single contig during genome assembly. NRPS BGCs are long (average length ~60 kb^45^) and repetitive (made up of multiple highly similar domains), making it difficult to assemble them into a single contig. Meleshko et al.^45^, recently developed the biosyntheticSPAdes tool for BGC reconstruction in short-read isolate assemblies, but at the same time acknowledged that short-reads metagenome assemblies are not adequate for full-length BGC identification. Even with biosyntheticSPAdes^45^, it remains difficult to capture long and repetitive BGCs within a single contig. With recent advances in long-read sequencing technologies, more contiguous microbial genome assemblies are becoming available^84,85^, increasing the power of NRPminer.

Another challenge in applications of NRPminer to complex microbiome data is that, with the current state of MS technology, many spectra originate from host molecules (in the case of host-associated microbiomes) or environmental contaminations. For example, the majority of spectra collected on human skin microbiome correspond to deodorants, shampoos, and other beauty products, rather than microbial products^86^. The advent of sensitive MS data acquisition techniques could enable capturing low abundant microbial products from complex environmental and host-oriented samples.

NRPminer only considers methylation and epimerization tailoring enzymes in the BGCs and does not recognize any other modification enzymes that modify NRPs, such as glycosylation and acylation^87^. These modifications can only be predicted as blind modifications using the modification-tolerant search of their corresponding spectral datasets against the input genomes.

Currently, NRPminer identifies ~1% of spectra of isolated microbes as NRPs. However, ~99% of spectra in these datasets remain unidentified, representing the dark matter of metabolomics. These spectra could represent primary metabolites (e.g. amino acids), other classes of secondary metabolites (e.g. RiPPs, polyketides, lipids, terpenes), media contaminations, and lower intensity/quality spectra that are difficult to identify. Thus, further advances in experimental and computational MS are needed toward a comprehensive illumination of the dark matter of metabolomics.

## Methods

### Outline of the NRPminer algorithm

NRPminer expands on the existing tools for automated NRP discovery^30,40^ by utilizing algorithms that enable high-throughput analysis and handle non-canonical assembly lines and PAMs. Below we describe various steps of the NRPminer pipeline:

(a) *Predicting NRPS BGCs in (meta)genome sequences by genome mining*. NRPminer uses Biopython^88^ and antiSMASH^17^ to identify the NRP-producing BGCs in the assembled genome. Given a genome (or a set of contigs), antiSMASH uses HMMs to find NRP-producing BGCs. The NRPminer software package also includes biosyntheticSPAdes^45^, a specialized short-read BGC assembler.

(b) *Predicting putative amino acids for each A-domain in the identified BGCs*. NRPminer uses NRPSpredictor2 (ref. ^15^) to predict putative amino acids for each position in an NRP. Given an A-domain, NRPSpredictor2 uses support vector machines (trained on a set of A-domains with known specificities) to predict the amino acids that are likely to be recruited by this A-domain. NRPSpredictor2 provides a specificity for each predicted amino acid that is based on the similarity between the analyzed A-domain and the previously characterized A-domains^16,18^. NRPminer uses NRPSpredictor2 (ref. ^15^) predictions to calculate the specificity scores for each predicted amino acid (see “Methods” section under “Specificity Scores of Putative Amino Acids)”.

(c) *Generating multiple NRPS assembly lines*. NRPminer generates multiple NRPS assembly lines by allowing for the option to either delete an entire ORFs, referred to as “orfDel” (Fig. 1c) or duplicate A-domains encoded by an ORF, referred to as “*orfDup*” (Fig. 1b). In the default “orfDel” setting, NRPminer considers all assembly lines formed by deleting up to two ORFs. With “orfDup” option, NRPminer generates non-canonical assembly lines that tandemly duplicate all A-domains appearing in a single ORF.

We represent an NRPS assembly line as a sequence of sets of amino acids, ${\mathscr{A}}$_1_*,…*,${\mathscr{A}}$_*k*_ where each ${\mathscr{A}}$_*i*_ represents the set of amino acids predicted for the *i*th A-domain of this assembly line along with their specificity scores. Given an NRPS assembly line with *k* A-domains and the corresponding sets ${\mathscr{A}}$_1_*,…*,${\mathscr{A}}$_*k*_, the set of all possible core NRPs for this assembly line is given by the cartesian product ${\mathscr{A}}$_1_$\times$*…*$\times {\mathscr{A}}$_*k*_. See “Methods” section under “Generating Assembly-lines Using NRPminer” for more information.

(d) *Filtering the core NRPs based on their specificity scores*. Supplementary Table 1 and Supplementary Table 7 illustrate that some BGC-rich genomes give rise to trillions of putative core NRPs. NRPminer uses the specificity scores of amino acids in each core NRP to select a smaller set of core NRPs for downstream analyses. Given an assembly line ${\mathscr{A}}$_1_*,…*,${\mathscr{A}}$_*k*_, for each amino acid $a\in {\mathscr{A}}$_*i*_ (*i* = 1*,…,k*), NRPminer first divides the specificity score of *a* by the maximum specificity score observed across all amino acids in ${\mathscr{A}}$_*i*_ (see “Methods” section under “Filtering the Core NRPs Based on their Specificity Scores)”; we refer to the integer value of the percentage of this number as the “normalized specificity score” of *a*. We define the score of a core NRP to be the sum of the normalized scores of its amino acids.

NRPminer uses a dynamic programming algorithm to efficiently find *N* highest-scoring core NRPs for further analyses (the default value is *N* = 1000), which enables peptidogenomics analysis of BGCs with many A-domains. The “Methods” section provides more information.

(e) *Identifying domains corresponding to known modifications and incorporating them in the core NRPs*. NRPminer searches each BGC for methylation domains (PF08242) and accounts for the possible methylations on corresponding residues for all resulting core NRPs (corresponding to +14.01 Da mass shift). NRPminer also searches for epimerization domains in each BGC (as well as dual condensation-epimerization domains) that provide information about the structure of the final NRP (d- or l-amino acids).

(f) *Generating linear, cyclic, and branch-cyclic backbone structures for each core NRP*. NRPminer generates linear and cyclic structures for all core NRPs. Similar to NRPquest^30^, whenever NRPminer finds a cytochrome P450 domain, it also generates branched-cyclic NRPs by considering a side-chain bond between any pair of residues in the peptide.

(g) *Modification-tolerant search of spectra against the constructed backbone structures*. Similar to PSMs in proteomics, a PSM in peptidogenomics is scored based on similarities between the theoretical spectrum of the peptide and the mass spectrum^43^ (see “Methods” section under “Forming Peptide-Spectrum-Matches (PSMs) and Calculating PSM score)”. The standard search of a spectrum against a peptide database refers to finding a peptide in the database that forms a highest-scoring PSM with this spectrum. Similarly, the modification-tolerant search of a spectrum against the peptide database refers to finding a variant of a peptide in the database that forms a highest-scoring PSM with this spectrum. In the case of NRPs, it is crucial to conduct modification-tolerant search in a blind mode in order to account for unanticipated PAMs in the mature NRP.

Existing peptidogenomics methods utilize a brute-force approach for modification-tolerant search, by creating a database of all possible unanticipated modifications^30^. For example, given a spectrum and a core NRP structure with *n* amino acids, these methods consider a modification of mass *δ* on all possible amino acids in the NRP, where *δ* is the mass difference between the spectrum and the NRP. Gurevich et al.^43^ developed the VarQuest tool for modification-tolerant search of large spectral datasets against databases of peptidic natural products that is two orders of magnitude faster than the brute-force approach. NRPminer utilizes VarQuest for identification of PAMs with masses up to MaxMass with the default value MaxMass=150 Da (see “Methods” section for more informatoin). This approach also allows NRPminer to identify loss or addition of an amino acid (for amino acids with molecular mass up to MaxMass Da). Note that, similar to identification of PAMs in linear proteomics^30^, MS-based methods for NRP discovery are limited to finding modification masses and cannot provide information about the exact chemistry of the identified modifications.

NRPminer has the one-vs-one mode for searching a spectral dataset against the genome corresponding to its producer. Additionally, NRPminer features the one-vs-all mode that a spectral dataset is searched against all genomes in the corresponding taxonomic clade (or any given set of genomes). One-vs-all is useful in cases when an entire BGC is not assembled in a single contig in the producer’s genome, but well-assembled in a related genome.

In scoring PSMs, NRPminer has a user-adjustable threshold for the accuracy of precursor and products ions, thus improving the accuracy of PSM scoring in the case of modification-tolerant search of high-resolution spectral datasets. This feature improves on NRPquest whose applications are largely limited to low-resolution spectra.

(h) *Computing statistical significance of PSMs*. NRPminer uses MS-DPR^89^ to compute *p* values of the identified PSMs. Given a PSM, MS-DPR computes the probability (*p* value) that a random peptide has a score greater than or equal to the PSM score (see “Methods” section under “Computing P-values and Peptide-Spectrum-Matches”). The default *p* value threshold (10^−15^) is chosen based on the previous studies where the *p* value cut-off 10^−15^ was necessary for reaching a false discovery rate (FDR) below 1% against NRPs^42,43^. Furthermore, NRPminer filters the PSMs based on the FDR values reported by VarQuest (default threshold 1%). The user can change the *p* value and FDR thresholds (using “—*p* value” and “—fdr” handles) depending on their study. *E*-values are also calculated by multiplying *p* values with the number of spectra and NRPs computed.

(i) *Expanding the set of identified NRPs using spectral networks*. Spectral datasets often contain multiple spectra originating from the same compound. NRPminer clusters similar spectra using MS-Cluster^60^ and estimates the number of distinct NRPs as the number of clusters. It further constructs the spectral network^50,57^ of all identified spectra and estimates the number of distinct NRP families as the number of connected components in this network.

Spectral networks reveal the spectra of related peptides without knowing their amino acid sequences^57^. Nodes in a spectral network correspond to spectra, while edges connect spectral pairs, i.e. spectra of peptides differing by a single modification or a mutation. Ideally, each connected component of a spectral network corresponds to a single NRP family^57^ representing a set of similar NRPs. In this study, we only report an identified NRP family if at least one NRP in the family is identified with a PSM *p* value at least 10^−20^. NRPminer utilizes spectral networks for expanding the set of identified NRPs.

### Sample preparation and MS experiments

*General experimental procedures*. ^1^H, ^13^C, HSQC, HMBC, HSQC-COSY, HSQC-TOCSY, and ROESY spectra were measured on Bruker AV500, AV600, and AV900 spectrometers, using DMSO-*d*_6_ and CDCl_3_ as solvent. Coupling constants are expressed in Hz and chemical shifts are given on a ppm scale. HRESIMS was performed on an UltiMate 3000 system (Thermo Fisher) coupled to an Impact II qTof mass spectrometer (Bruker Daltonik GmbH). Preparative HPLC was performed on an Agilent 1260 HPLC/MS system with a ZORBAX StableBond 300 C18 (21.2 mm × 250 mm, 7.0 µm, Agilent). Semi-preparative HPLC was performed on an Agilent 1260 HPLC/MS system with a ZORBAX StableBond 300 C18 (9.4 mm × 250 mm, 5.0 µm, Agilent).

Below we describe sample preparation and mass spectra generation for all analyzed datasets in more details.

*XPF***:** A total of 27 strains from soil nematode symbiont *Xenorhabdus* and *Photorhabdus* families were grown in lysogeny broth and agar and were extracted with methanol as described previously (Supplementary Table 1). Briefly, the crude extracts were diluted 1:25 (vol/vol) with methanol and analyzed by UPLC-ESI coupled with Impact II qTof mass spectrometer. MS dataset spectra_XPF_^31^ contains 27 spectral sub-datasets representing each sample for a total of 263,768 spectra across all strains (GNPS-accession #: MSV000081063). The genome_XPF_ dataset contains 27 draft genomes generated by DNA sequencing from the same samples as reported by Tobias et al.^31^ (available from RefSeq^82^). See the sections below for detailed information about experiments regarding protegomycin and xenoamicin-like families, respectively.

*SkinStaph*: A total of 171 *Staphylococcus* strains isolated from skin of healthy individuals were grown in 500 mL Tryptic Soy Broth (TSB) liquid medium in Nunc 2.0 mL DeepWell plates (Thermo Catalog# 278743) by Zhou et al.^90^. An aliquot of each culture was used to measure optical density. Cultures that effectively grew were transferred to a new deep well plate. Cultures were placed in a −80 °C freezer for 10 min and then allowed to thaw at room temperature three times, to lyse bacterial cells. Two hundred microliters of the supernatant collected from cell cultures were filtered using a Phree Phospholipid Removal kit (Phenomenex). Sample clean up was performed following the manufacturer’s protocol described here (https://phenomenex.blob.core.windows.net/documents/c1ac3a84-e363-416e-9f26-f809c67cf020.pdf). Briefly, the Phree kit plate was conditioned using 50% MeOH; bacterial supernatant were then added to the conditioned wells followed by sample clean up using 100% MeOH (a 4:1 v/v ratio of MeOH:bacterial supernatant). The plate was centrifuged 5 min at 500*g* and the clean up extracts were lyophilized using a FreeZone 4.5 L Benchtop Freeze Dryer with Centrivap Concentrator (Labconco). Wells were resuspended in 200 µL of resuspension solvent (80% MeOH spiked with 1.0 µM Amitriptyline), vortexed for 1 min, and shaken at 2000 r.p.m. for 15 min at 4 °C. One hundred and fifty microliters of the supernatant was transferred into a 96-well plate and maintained at 20 °C prior to liquid chromatography tandem mass spectrometry (LC-MS/MS) analysis. Bacterial extracts were analyzed using a ThermoScientific UltiMate 3000 UPLC system for liquid chromatography and a Maxis Q-TOF (Quadrupole-Time-of-Flight) mass spectrometer (Bruker Daltonics), controlled by the Otof Control and Hystar software packages (Bruker Daltonics) and equipped with ESI source. Untargeted metabolomics data were collected using a previously validated UPLC-MS/MS method^91,92^. The spectra_SkinStaph_ dataset contains 2,657,398 spectra from bacterial extracts of 171 *Staphylococcus* strains (GNPS- accession #: MSV000083956). The genome_SkinStaph_ dataset contains draft genomes of these species (available from RefSeq).

*SoilActi*: A total of 20 strains of soil-dwelling *Actinobacteria* were grown on A1, MS, and R5 agar, extracted sequentially with ethyl acetate, butanol, methanol, and analyzed on Agilent 6530 Accurate-Mass QTOF spectrometer coupled with Agilent 1260 LC System. The spectra_SoilAct_ dataset contains 362,421 spectra generated from extracts of these 20 Actinobacteria strains (GNPS-accession #: MSV000078604 (ref. ^93^)) includes 20 sub-datasets representing each strain. The genome_SoilActi_ dataset contains draft genomes of these strains (available via RefSeq).

*TinyEarth*: A total of 23 bacterial strains extracted from the soil in Wisconsin were grown in microscale liquid cultures and extracted using solid phase extraction with in methanol. These samples were and analyzed by LC-MS/MS on a Thermo Fisher Q-Exactive mass spectrometer coupled with a Thermo Fisher Vanquish UPLC system. The spectra_TinyEarth_ dataset contains 380,414 spectra generated from extracts of these 23 strains (GNPS-accession #: MSV000084951) includes 23 sub-datasets representing each strain (4 *Bacillus*, 16 *Pseudomonas*, 1 *Buttiauxella*, and 1 *Citrobacter*). The genome_TinyEarth_ dataset contains draft genomes of these strains (available via Gold OnLine Database^83^ under study ID Gs0135839).

### Additional analyses for protegomycin family

*X*. *doucetiae*-Δ*hfq* was constructed as described before^94^. Exchange of the natural promoter against the inducible P_BAD_ was performed as described^95^. Briefly, the first 598 base pairs of *prtA* were amplified with primer pEB_317-fw TTTGGGCTAACAGGAGGCTAGCATATGAGAATACCTGAAGGTTCG and PEB_318-rv TCTGCAGAGCTCGAGCATGCACATCGTAATGAAACGAGTTCAGG (Supplementary Table 11). The resulting fragment was cloned via hot fusion cloning into pCEP-km. The resulting construct pCEP *prtA*-km was transformed into *E*. *coli* S17-1 λpir resulting in *E*. *coli* pCEP_*prtA*. Conjugation of this strain with *X*. *doucetiae* wt or *X*. *doucetiae*-Δ*hfq* was followed by integration of pCEP_*prtA*-km into the acceptors genome via homologous recombination^94,95^. In *X*. *doucetiae*-Δ*hfq*-P_*BAD*_-*prtA* the production of protegomycin was induced by adding 0.2% l-arabinose into the fresh inoculated medium^94^.

For large-scale production of protegomycin, 6 × 1 L LB medium was inoculated with *X*. *doucetiae*-Δ*hfq*_P_*BAD*_-*prtA* preculture 0.02%. Two percent Amberlite^®^ XAD-16 adsorber resin was added and the production was induced with 0.2% l-arabinose. The cultures were constantly shaked at 130 r.p.m. at 30 °C. After 72 h the XAD beads were harvested and protegomycins extracted using 3 L of methanol. The solvent was evaporated, and the crude extract was used for isolation and analysis of protegomycin derivatives. Part of the crude extraction was purified by preparative HPLC with a gradient mobile from 5 to 95% ACN in H_2_O (v/v) in 30 min followed by semi-preparative HPLC (ACN–H_2_O, 35–45% in 30 min, v/v) to yield PRT-1037 (24.4 mg).

For structure elucidation and determination of incorporated C- and N-atoms and amino acids into protegomycins, cultivation of *X*. *doucetiae*-Δ*hfq*_P_*BAD*_-*prtA* and *X*. *doucetiae*_ P_*BAD*_-*prtA*, induced with 0.2% l-arabinose was performed in 5 mL LB (^12^C), ^13^C-, and ^15^N-isogro^®^ medium (Sigma-Aldrich). The cultures were supplemented with 2% Amberlite^®^ XAD-16 adsorber resin. To analyze the incorporated amino acids, induced mutants were grown in LB medium supplemented with selected ^13^C-labeled amino acids with a concentration of 2 mM. After 48 h cultivation at 30 °C, constantly shaking at 200 r.p.m., Amberlite^®^ XAD-16 beads were harvested and extracted with 5 mL MeOH for 45 min. Samples were taken from the filtered extracts and centrifuged for 15 min at 17,000*g* for further HPLC-MS analysis (Dionex Ultimate 3000 coupled to a Bruker AmaZon X ion trap). Generated HPLC-MS data were interpreted as described previously^94,96^.

### Additional analyses for Xenoamicin-like family

*Cultivation of strains*: *Xenorhabdus* KJ12.1 was routinely cultivated in Luria-Bertani (LB) medium (pH 7.0) at 30 °C and 200 r.p.m. on a rotary shaker and on LB agar plates at 30 °C. Inverse feeding experiments were applied in either ISOGRO^® 13^C medium, ISOGRO^® 15^N medium. Fifty microliters ISOGRO^®^ medium was prepared with ISOGRO^®^ powder (0.5 g), K_2_HPO_4_ (1.8 g/L), KH_2_PO_4_ (1.4 g/L), MgSO_4_ (1 g/L), and CaCl_2_ (0.01 g/L) solved in water. Feeding experiments in ISOGRO^® 13^C medium supplemented with ^12^C amino acids was inoculated with ISOGRO^®^ washed overnight cultures.

Production cultures were grown in LB media containing 2% Amberlite^®^ XAD-16 resin inoculated with 1% overnight culture. Promotor exchange mutants were induced with 0.2% arabinose at the beginning of the cultivation. Resin beads and bacterial cells were harvested by centrifugation after 72 h cultivation time, washed twice with one culture volume methanol. The crude extracts were analyzed by means of MALDI-MS and HPLC-MS (Bruker AmaZon).

*HPLC-based purification*: XAM-1320 was isolated by a two-step chromatography. Strain KJ12.1 was cultivated in a BIOSTAT A plus fermenter (Sartorius) equipped with a 2-L vessel in 1.5 L of LB broth at 30 °C for 12 h. For the inoculation, 1% overnight preculture was used and 2% XAD-16 were added. Additionally, 10 g of glucose and 5 mL Antifoam 204 (Sigma-Aldrich) were added. The fermentation was performed with an aeration of 2.25 vvm, constant stirring at 300 rpm and at pH 7, stabilized by the addition of 0.1 N phosphoric acid or 0.1 N sodium hydroxide. The XAD resin was washed with methanol to get the extract after evaporation. Xenoamicin III A was isolated by a two-step chromatography. In the first step the extract was fractionated with a 5–95% water/acetonitrile gradient over 15 min on a Luna C_18_ 10 μm 50 × 50 mm column (Phenomenex). In the second step XAM-1320 was isolated with a 40–60% water–acetonitrile gradient over 19 min on Luna C_18_ 5 μm 30 × 75 mm column (Phenomenex).

*MS analysis***:** MS analysis was carried out by using an Ultimate 3000 LC system (Dionex) coupled to an AmaZon X electronspray ionization mass spectrometer (Bruker Daltonics). Separation was done on a C18 column (ACQITY UPLC BEH, 1.7 mm, 2.1 × 50 mm, flow rate 0.4 mL/min, Waters). Acetonitrile/water containing 0.1% formic acid was used as a mobile phase. The gradient started with 5% acetonitrile continuous over 2 min. Over 0.5 min under a linear gradient acetonitrile reaches 40%. Following an equilibration phase over 1.5 min with 40% acetonitrile takes place. For separation a linear gradient from 40–95% acetonitrile over 10.5 min was used. The gradient ends up with 95% acetonitrile continuous over 1.5 min. Collision-induced dissociation (CID) was performed on ion trap in the AmaZon X in positive mode. HR-ESI-HPLC-MS data were obtained on a LC-coupled Impact II ESI-TOF spectrometer (Bruker Daltonics).

*Advanced Marfey’s method*: The advanced Marfey’s method to determine the configurations of the amino acid residues was performed as described previously^64^.

### Calculating specificity scores of putative amino acids

During NRP synthetase, the A-domains recognize and activate the specific amino acid that will be appended to the growing peptide chain by other NRPS enzymes. Conti et al.^97^ showed that some residues at certain positions on each A-domain are critical for substrate activation and bonding; they reported 10 such positions. Stachelhaus et al.^98^ showed that for each A-domain AD, the residues at these decisive 10 positions can be extracted to form a specificity-conferring code called non-ribosomal code of AD. They demonstrated that the specificity of an uncharacterized A-domain can be inferred based on the sequence similarity of its non-ribosomal code to those of the A-domains with known specificities^98^.

Given an input A-domain AD, NRPSpredictor2 (ref. ^15^) first compares the sequence of the non-ribosomal code of AD to those of the already characterized A-domains in the NRPSpredictor2 (ref. ^15^) database. Afterwards, for each amino acid *a*, NRPSpredictor2 (ref. ^15^) reports the Stachelhaus score of (specificity of) *a* for A-domain AD, that is (the integer value of) the percentage of sequence identity between the non-ribosomal code of AD and that of the most similar A-domain within NRPSpredictor2 (ref. ^15^) search space that encodes for *a*.

Furthermore, Rausch et al.^99^ expanded the set of specificity-conferring positions on A-domains to 34 residue positions and proposed a predictive model trained on residues at these 34 positions (instead of just the 10 included in Stachelhaus code) to provide further specificity predictions^15^. Given an A-domain, they used a Support Vector Machine (SVM) method trained on previously annotated A-domains. For each input A-domain, this approach^99^ predicts three sets of amino acids in three different hierarchical levels based on the physio-chemical properties of the predicted amino acids: large clusters^99^ (each large cluster is at most eight amino acids), small clusters^99^ (each small cluster is at most three amino acids), and single amino acid prediction (the single amino acid most likely to be activated by the given A-domain), as described by Rausch et al.^99^ For a given A-domain AD, we use the terms large cluster, small cluster, and single prediction of AD to describe the sets of amino acids predicted at each of these hierarchical levels. While Rausch et al.^99^ demonstrated that their approach reports better specificity predictions for less commonly observed A-domains, they also showed that integrating their score with the sequence similarity approach described by Stachelhaus et al.^98^ results in the highest accuracy^99^.

Similar to the approach used by NRP2Path^40^, NRPminer combines the two predictions provided by NRPSpredictor2 (ref. ^15^). Given an A-domain AD and an amino acid *a*, NRPminer defines the SVM score of *a* for AD to be 100 if *a* matches the single amino acid prediction, 90 if *a* appears in the small cluster predictions, and 80 if *a* appears in the large cluster. If *a* does not appear in any of these sets, NRPminer defines the SVM score of *a* for AD to be 0. The total number of amino acids per A-domain with SVM score above 0 is at most 12 (considering all three sets of amino acids). For a given A-domain AD, NRPminer only considers amino acids with a predicted Stachelhaus score>50 and a predicted SVM score>0 for AD. Finally, NRPminer defines the specificity (or NRPSpredictor2) score of *a* for AD as the mean of Stachelhaus and SVM scores of *a* for AD.

### Generating NRPS assembly lines using NRPminer

Given a BGC, an assembly line refers to a sequence of NRPS modules in this BGC that together assemble the core NRP. NRPminer represents an assembly line as the sequence of A-domains appearing in its NRP modules and allows a user to explore various assembly lines using OrfDel and OrfDup options. Each portion of an NRPS that is encoded by a single ORF is an *NRPS subunit*. With OrfDel option, NRPminer considers skipping up to two entire NRPS subunits. Figure S31b illustrates the assembly lines generated from surugamide BGC by deleting A-domains appearing on zero, one, and two NRPS subunits, out of the four NRPS subunits encoded by the four ORFs appearing in this BGC. For example, for surugamide BGC with four ORFs (shown in yellow in Fig. S31a), with “orfDel” option, NRPminer generates six NRP assembly lines formed by two ORFs (Fig. S31b), four assembly lines formed by three ORFs, and one canonical assembly line formed by all four ORFs. Figure S31c illustrates that for surugamide NRPS assembly line formed by SurA and SurD genes, ${\mathscr{A}}$_1_ = {val, ile, abu}, ${\mathscr{A}}$_2_ = {phe, tyr, bht}, etc.

Using the OrfDup option, NRPminer also considers assembly lines that are generated by multiple incorporation of A-domains appearing on a single NRPS subunit. For example, Supplementary Fig. 7 shows the lugdunin BGC with four ORFs encoding for five A-domains. This figure illustrates that using OrfDup option, NRPminer forms nine assembly lines: one representing the canonical assembly line (each NRPS subunit appears once), four assembly lines that are generated by duplicating the A-domains appearing in one NRPS subunit once (one subunit appearing two times in tandem), and four non-canonical assembly lines by duplicating them twice (one subunit appearing three times in tandem). NRPminer considers all assembly lines made up of at least three and at most 20 NRPS modules.

### Filtering the core NRPs based on their specificity scores

Given an NRPS assembly line *A* = *A*_*1*_*,…,A*_*n*_, where *A*_*i*_ is the set of amino acids predicted for the *i*th A-domain of *A*, for every *a ∈A*_*i*_ (*i* = 1,…,*n*), let SpecificityScore(*Ai*) (a) be the specificity score of *a* for the *i*th A-domain of *A* as described in Supplementary Note 3. Then, for each integer *1* *≤* *i* *≤* *n* and *a* *∈* *A*_*i*_, we define normalized specificity score of *a* for *i*th A-domain of *A*, denoted by *S*_*A*_ *(i,a)*, to be the nearest integer to the following value:

$$\frac{{\rm{Specificity}}\,{{\rm{Score}}}_{{A}_{i}}(a)}{\mathop{\max }\limits_{b\in {A}_{i}}{\rm{Specificity}}\,{{\rm{Score}}}_{{A}_{i}}(b)}\times 100$$

We use this scoring function (instead of SpecificityScore) to reduce the bias towards the more frequently observed A-domains that usually result in higher specificity scores compared to the less commonly observed ones, which do not have closely related A-domains in NRPSpredictor2 training datasets^15^. Consider the assembly line of cyclic surugamides A–D shown in Fig. S31c (corresponding to *SurA*-*SurD* gene pairs in surugamide BGC) which is made up of eight A-domains, we refer to this assembly line by SurugamideAL. Table 2 presents the values of *S*_SurugamideAL_ for integers 1 ≤ *i* ≤8 and (at least) the three amino acids with the highest normalized specificity scores for each A-domain in this assembly line.

Given *A* = A_*1*_,*…,A*_*n*_ we call the set of all core NRPs generated by the cartesian product *A*_*1*_×*…*×*A*_*n*_ as the core NRPs of A. For each core NRP of *A, a*_*1*_*a*_*2*_*…a*_*n*_, we define the adenylation score of *a*_*1*_*a*_*2*_*…a*_*n*_, denoted by Score_*A*_*(a*_*1*_*a*_*2*_*…a*_*n*_*)*, to be the sum of the normalized specificity scores of all of its amino acids:

$${\mathrm{{Score}}_A}(a_1a_2\ldots a_n)=\,\mathop{\sum}_{i=1}^{n}S_A(i,a_i)$$

Therefore, given assembly line SurugamideAL and core NRP, *P*=IAIIKIFL (the core NRP corresponding to surugamide A), Score_SurugamideAL_(*P*) = 80 + 100 + 100 + 100 + 100 + 100 + 100 + 86 = 766. Note that, for any assembly line *A*, the maximum value of Score_*A*_ denoted by maxScore_*A*_=${\sum}_{i=1}^{n}{\max}_{{a}_{i}\in {A}_{i}}{S}_{{\boldsymbol{A}}}(i,{a}_{i})=100n$.

For many organisms, the total number of possible core NRPs is prohibitively large, making it infeasible to conduct search against massive spectral repositories. Currently, even the fastest state-of-the-art spectral search methods are slow for searching millions of input spectra against databases with over 10^5^ peptides in a modification-tolerant manner as the runtime grows exceedingly large when the database size grows^43^. Supplementary Tables S2 and S7 shows that for 24 (22) out for 27 organisms in XPF dataset and 9 (7) out of 20 organisms in SoilActi dataset, the total number of core NRPs exceed 10^5^ (10^6^). Therefore, to enable scalable peptidogenomics for NRP discovery, for each constructed assembly line NRPminer, selects a set of candidate core NRPs. To do so, NRPminer starts by finding the number of core NRPs of *A* according to their adenylation scores (Problem 1) and then it uses these numbers for generating all core NRPs of *A* with adenylation scores higher than a threshold (Problem 2).

**Problem 1**. Given *A* = *A*_*1*_*,…,A*_*n*_ and a positive integer *s*, find the number of all core NRPs of *A* with adenylation score equal to *s*.

Let $k=\mathop{\max }\limits_{i\in \{1,{\mathrm{..}}.,n\}}(|{A}_{i}|)$ where |${A}_{i}$| shows the number of amino acids in *A*_*i*_. For any positive integers *i* and *s* satisfying,1 ≤ *i* *≤* *n* and *s* ≤ maxScore_*A*_, let numCoreNRPs_A_ (*i, s*) denote the number of core NRPs, of assembly line *A*_1_,...,A_*i*_ with ${{\rm{Score}}}_{{A}_{1},{\mathrm{..}}.,{A}_{i}}$ equal to *s*. Let numCoreNRPs_A_ (0,*s*) = 0 for any positive integer *s*, and numCoreNRPs_A_ (*i, s*) = 0 for any integer *s* *<* 0, across all possible values of *i*. Then, for any positive integers *i* and *s* satisfying 1 ≤ *i* ≤ *n* and 0 < *s* ≤ maxScore_*A*_, we have

1 $${{\mathrm{numCoreNRPs}}}_{A}(i,s)={\mathop{\sum} \limits_{{{a}_{i}}\in {{A}_{i}}}}{{\mathrm{numCoreNRPs}}}_{A}(i-1,s-{S}_{A}(i,{a}_{i}))$$

Using recursive formula (1), NRPminer calculates numCoreNRPs_A_ using parametric dynamic programming in a bottom-up manner: NRPminer first, computes numCoreNRPs*A*(*1,s*), for all positive integers *s≤*maxScore_*A*_. then proceeds to numCoreNRPs*A*(2,s) for all such *s*, and so on, computing numCoreNRPs_*A*_(*n*,*s*) for all such 0 < *s*. Using this approach, for each value of *i* and *s*, NRPminer computes numCoreNRPs_A_ (*i,s*) by summing over at most *k* values. Therefore, NRPminer calculates all values of numCoreNRPs_*A*_ with time complexity *O(k* *×* *n* *×* maxScore_A_*)*.

Given a positive integer *N* < 10^5^, let *scor*_(A,N)_ be the greatest integer *s*′ ≤ maxScore_*A*_ such that, *N* ≤ Σ_*s*'≤*s*≤maxScore_numCoreNRPs_*A*_ (*n,s*).

Then, we define

2 $${{\rm{thresholdScore}}}_{A}(N)=\left\{\begin{array}{l}{{\rm{score}}}_{{\rm{N}}}\,\,\,{\text{if}}\;{{\rm{score}}}_{N} \,<\, {{\rm{score}}}_{{10}^{5}}\\ {{\rm{score}}}_{N}-1\,\,\,\,\,\,\,{\text{if}}\;{{\rm{score}}}_{N}={{\rm{score}}}_{{10}^{5}}\end{array}\right.$$

NRPminer selects, candidateCoreNRPs_A_(*N*), defined as the set of all core NRPs of *A*, with adenylation score at least thresholdScore_*A*_ (*N*). NRPminer selects core NRPs candidateCoreNRPs_*A*_(*N*) for downstream spectral analyses. Using this approach, NRPminer is guaranteed to be scalable as at most 10^5^ candidate core NRPS are explored per assembly line.

Table 3 presents the values of numCoreNRPs_SurugamideAL_(8*,s*) for various values of *s*. Note that, this table presents the number of core NRP only for a single assembly line, SurugamideAL, corresponding to cyclic surugamides (surugamide A–D). In total, 14,345 core NRPs were retained from the original 3,927,949,830 core NRPs of the 11 assembly lines of surugamide’s BGC.

**Table 3:** Number of core NRPs of SurugamideAL (assembly line corresponding to cyclic surugamides A–D) according to their adenylation scores.

*s*	800	790	788	786	780	778	776	774	772	Total
${\mathrm{numCoreNRPs}}_{\mathrm{SurugamideAL}}(8,{{s}})$	24	48	24	192	24	48	384	192	168	1104

Only values of *s* with non-zero number of cores and corresponding to the top 1000 high-scoring core NRPs are shown^103^.

**Problem 2**. Given an assembly line *A* and a positive integer *N*, generate candidateCoreNRPs_*A*_(*N*), defined as all core NRPs of *A* with adenylation scores at least thresholdScore_*A*_(*N*).

NRPminer follows a graph-theoretic approach to quickly generate candidateCoreNRPs_*A*_(*N*) by using the computed values of numCoreNRPs. Let *G*(*A*) be the acyclic directed graph with nodes corresponding to pairs of positive integers *i* ≤ *n* and *s* *≤* maxScore_*A*_, such that numCoreNRPs_*A*_(*i,s*) > 0, denoted by ${v}_{i,s}$. For every node ${v}_{i,s}$ (*i* = 1,…,*n*) and every $a\in {A}_{i}$ such that numCoreNRPs_*A*_(*i−1,s−S*_*A*_*(i,a)*) > 0, there exists a directed edge from ${v}_{i-1,s-{S}_{A}(i,a)}$ to ${v}_{i,s}$. Let Source be ${v}_{0,0}$ and let Sink be the set of all nodes ${v}_{n,s}$ such that thresholdScore_*A*_*(N)* $\le s$. We call each directed path in *G*(*A*) from Source to the nodes in Sink as a candidate path of *G(A)*.

Each candidate path of *G*(*A*) corresponds to a distinct core NRP of *A* with adenylation score at least thresholdScore_*A*_(*N*) and vice versa. Therefore, the problem of finding all core NRPs of *A* with adenylation score at least thresholdScore_*A*_(*N*) corresponds to the problem of finding all candidate paths of *G*(*A*). While enumerating all paths with *n* nodes in a directed acyclic graph can grow exponentially large (as there can be exponentially many such paths), but due to our choice of thresholdScore_*A*_(*N*), the number of candidate paths of *G*(*A*) is bound by 10^5^ (or *N* if ${{\mathrm{score}}_{N}}={{\mathrm{score}}}_{{10}^{5}}$). NRPminer uses the default value *N* = 1000. Moreover, *n* *≤* 20 (only assembly lines made up of up to 20 A-domains are considered) and *k* $\le$ 12.

### Forming PSMs and calculating PSM scores

PSMs and their PSM scores are described by Gurevich et al.^43^. Given a peptide *P* (with any backbone structure), we define Mass(*P*) as the sum of masses of all amino acids present in P. Furthermore, we define the graph of *P* as a graph with nodes corresponding to amino acids in *P* and edges corresponding to *generalized peptide bonds* as described in Mohimani et al.^100^. Then, we define theoretical spectrum of P (as opposed to the experimental spectrum) is the set of masses of all fragments generated by removing pairs of bonds corresponding to two-cuts in graph of *P* or by removing single bonds corresponding to the bridges in the graph of *P* as described by Mohimani et al.^100^. Each mass in this set is called a theoretical peak. Then, given the spectrum *S*, if precursor mass of *S* and Mass(*P*) are within a threshold Δ Da (where the default value of Δ is 0.02), we define the score of *P* against *S*, shown by SPCScore(*P*,*S*), as the number of peaks in theoretical spectrum of *P* that are within *ε* Da of a peak in *S* (where the default value of *ε* is 0.02). NRPminer only considers high-resolution data.

If (*A*_1_, …, *A*_*n*_) is the list of amino acid masses in a peptide *P*, we define Variant(*P*,*i*,*δ*) as (*A*_1_,…, *A*_i_ + *δ*, …, *A*_*n*_), where *P* and Variant(*P*,*i*,*δ*) have the same topology and *A*_*i*_ + *δ* ≥ 0. VariableScore(*P*,*S*) is defined as

$$({\mathrm{{SPCScore}}}({\mathrm{{Variant}}}(P,i,\omega ),S))$$

where *ω* is Mass(*P*) − Mass(*S*) and *i* varies from 1 to *n* (*n* stands for the number of amino acids in the peptide *P*)^43^. We define a variant of peptide *P* derived from a spectrum *S* as Variant(*P*,*i*,*ω*) of peptide *P*, which maximizes SPCScore(Variant(*P*,*i*,*ω*),*S*) across all positions *i* in *P*. For simplicity, we refer to this variant as Variant(*P,S*). Given *P* and *S*, VarQuest^43^ uses a heuristic approach to efficiently find Variant(*P,S*).

NRPminer uses VarQuest^43^ to perform modification-tolerant search of the input spectral datasets against the constructed peptide structures generated from selected core NRPs (see the NRPminer step “generating linear, cyclic, and branch-cyclic backbone structures for each core NRP” in Fig. 2 and “Method section”). Given a positive number MaxMass representing the maximum allowed modification mass (default value of MaxMass = 150), for each constructed structure *P* and input spectrum *S*, if |Mass*(P)−*Mass*(S)*|$\le$MaxMass, NRPminer uses VarQuest^43^ to find the Variant*(P, S)*. In this context, Variant*(P,S)* represents the mature NRP with a single PAM on *P* that resulted in the mass difference |Mass*(S)*−Mass*(P)*|. Similar idea has been applied to identification of post-translational modifications in traditional proteomics^49,101^.

### Computing *P* values of PSMs

NRPminer uses the MS-DPR^89^ to compute the statistical significance (*p* value) of each identified PSM. Given ${\mathrm{{PSM}}}(P,S)$ where *P* is a peptide with length *n* and *S* is a spectrum, MS-DPR estimates the probability that a random peptide, say $P^{\prime}$, with length $n$, has ${\mathrm{{SPCScore}}}(P^{\prime},S)\ge {\mathrm{{SPCScore}}}(P,S)$. We refer to this probability as *p* value of ${\mathrm{{PSM}}}(P,S)$. Monte Carlo approach can estimate the *p* value by generating a population of random peptides with length $n$, and scoring them against the spectrum $S$.

In case of MS-based experiments for identifying NRPs^102^, we are often interested in PSMs with *p* value < 10^−12^ (the *p* values corresponding to high-scoring PSMs)^102^. But naive Monte Carlo approach is infeasible for evaluating such rare events as the number of trials necessary for exploring such low *p* value is too large to practically explore. To resolve this issue, MS-DPR^89^ uses multilevel splitting technique for estimating the probability of rare event (i.e. high-scoring PSMs). MS-DPR^89^ constructs a Markov Chain over the scores of all peptides with length $n$ and then uses multilevel splitting to steer toward peptides that are more likely to form high PSM scores against $S$. Using this approach, MS-DPR^89^ can efficiently estimate an extreme tail of the scores of all possible peptides against $S$ which is then used to compute the *p* value of the ${\mathrm{{PSM}}}(P,S)$.

### Reporting summary

Further information on research design is available in the Nature Research Reporting Summary linked to this article.

## Supplementary information

Supplementary Information

Reporting Summary

## Data Availability

All described datasets are available through the corresponding public repositories. XPF, SkinStaph, SoilActi, and TinyEarth datasets are available via MSV000081063, MSV000083956, MSV000078604, and MSV00084951 GNPS-accessions, respectively.

## Code availability

NRPminer is available as both a stand-alone tool (https://github.com/mohimanilab/NRPminer) and as a web application via GNPS in silico toolbox. We used NPDtools, antiSMASH 3.5.0 and Biopython 1.78.
