# Publisher Correction: Integrating genomics and metabolomics for scalable non-ribosomal peptide discovery

**DOI:** 10.1038/s41467-021-24441-w

**Published:** 2021-07-08

**Authors:** Bahar Behsaz, Edna Bode, Alexey Gurevich, Yan-Ni Shi, Florian Grundmann, Deepa Acharya, Andrés Mauricio Caraballo-Rodríguez, Amina Bouslimani, Morgan Panitchpakdi, Annabell Linck, Changhui Guan, Julia Oh, Pieter C. Dorrestein, Helge B. Bode, Pavel A. Pevzner, Hosein Mohimani

**Affiliations:** 1 Bioinformatics and Systems Biology Program, University of California San Diego, La Jolla, CA USA; 2 Center for Microbiome Innovation, University of California at San Diego, La Jolla, CA USA; 3 Computational Biology Department, School of Computer Science, Carnegie Mellon University, Pittsburgh, PA USA; 4 Molecular Biotechnology, Department of Biosciences, Goethe University Frankfurt, Frankfurt am Main, Germany; 5 Center for Algorithmic Biotechnology, Institute of Translational Biomedicine, St. Petersburg State University, St Petersburg, Russia; 6 Tiny Earth Chemistry Hub, University of Wisconsin–Madison, Madison, WI USA; 7 Collaborative Mass Spectrometry Innovation Center, Skaggs School of Pharmacy and Pharmaceutical Sciences, University of California San Diego, La Jolla, CA USA; 8 The Jackson Laboratory of Medical Genomics, Farmington, CT USA; 9 Buchmann Institute for Molecular Life Sciences (BMLS), Goethe University Frankfurt & Senckenberg Research Institute, Frankfurt am Main, Germany; 10 Max-Planck-Institute for Terrestrial Microbiology, Department for Natural Products in Organismic Interactions, Marburg, Germany; 11 Department of Computer Science and Engineering, University of California San Diego, La Jolla, CA USA

**Keywords:** Data mining, Software, Natural products

Correction to: *Nature Communications* 10.1038/s41467-021-23502-4, published online 28 May 2021.

The original version of this Article omitted Bahar Behsaz and Edna Bode as equally contributing authors.

The original version of this Article contained an error in the author affiliations.

Pavel A. Pevzner was incorrectly associated with Computational Biology Department, School of Computer Science, Carnegie Mellon University, Pittsburgh, PA, USA.

This has now been corrected in both the PDF and HTML versions of the Article.

