# Supplementary material for: Integrating genomics and metabolomics for scalable non-ribosomal peptide discovery
Source: Nat Commun. 2021 May 28;12:3225. doi: 10.1038/s41467-021-23502-4 (PMC8163882; doi:10.1038/s41467-021-23502-4)
Supplement: Supplementary file 1 — Supplementary Information [file 41467_2021_23502_MOESM1_ESM.pdf]

Supplementary Information

Integrating Genomics and Metabolomics for Scalable Non-Ribosomal Peptide Discovery

Bahar Behsaz*[1,2,11], Edna Bode*[3], Alexey Gurevich[4], Yan-Ni Shi[3], Florian Grundmann[3], Deepa Archarya[5], Andrés Mauricio Caraballo-Rodríguez[6], Amina Bouslimani[6], Morgan Panitchpakdi[6], Annabell Linck[3], Changhui Guan[7], Julia Oh[7], Pieter C. Dorrestein[2,6], Helge B. Bode[3,8,9,+], Pavel A. Pevzner[2,10,+], Hosein Mohimani[11,+]

[1] Bioinformatics and Systems Biology Program, University of California San Diego, La Jolla, USA

[2] Center for Microbiome Innovation, University of California at San Diego, La Jolla, USA

[3] Molecular Biotechnology, Department of Biosciences, Goethe University Frankfurt, Frankfurt am Main, Germany

[4] Center for Algorithmic Biotechnology, Institute of Translational Biomedicine, St. Petersburg State University, St Petersburg, Russia

[5] Tiny Earth Chemistry Hub, University of Wisconsin–Madison, Madison, USA

[6] Collaborative Mass Spectrometry Innovation Center, Skaggs School of Pharmacy and Pharmaceutical Sciences, University of California San Diego, La Jolla, USA

[7] The Jackson Laboratory of Medical Genomics, Farmington, USA

[8] Buchmann Institute for Molecular Life Sciences (BMLS), Goethe University Frankfurt & Senckenberg Research Institute, Frankfurt am Main, Germany

[9] Max-Planck-Institute for Terrestrial Microbiology, Department for Natural Products in Organismic Interactions, Marburg, Germany

[10] Department of Computer Science and Engineering, University of California San Diego, La Jolla, USA

[11] Computational Biology Department, School of Computer Science, Carnegie Mellon University, Pittsburgh, USA.

+ Correspondence: helge.bode@mpi-marburg.mpg.de, ppevzner@ucsd.edu, hoseinm@andrew.cmu.edu.

Supplementary Figures 1-32

Supplementary Tables 1-13

Supplementary Figures

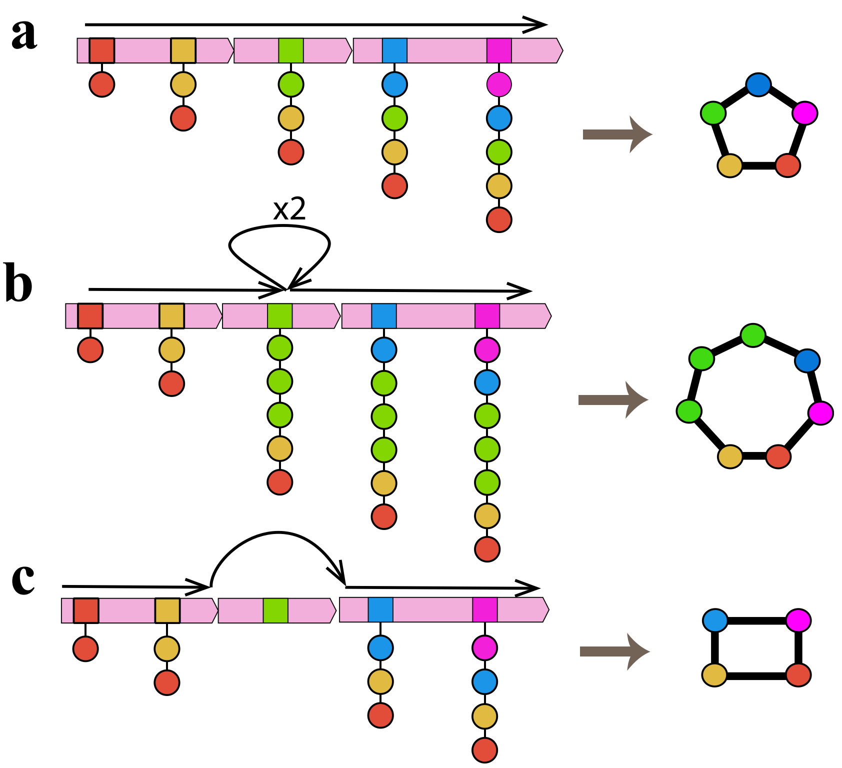

Supplementary Figure 1. Schematic examples of canonical and non-canonical NRPS assembly lines. Squares represent A-domains and circles represent amino acids (different amino acids are shown by different colors). Each amino acid is colored by the same color as the corresponding A-domain. In each panel, the final NRP is represented by its amino acids with amide bonds shown with black lines. (**a**) A canonical assembly line where each A-domain adds one amino acid to the growing structure. (**b**) A non-canonical assembly line where a single A-domain (on one ORF) loads a series of three amino acids (the loop shows the repeat of A-domain on the assembly line) to the growing structure also referred to as stuttering in polyketide synthases[1,2]. (**c**) A non-canonical assembly line where the A-domain appearing on one ORF is skipped in the final NRP.

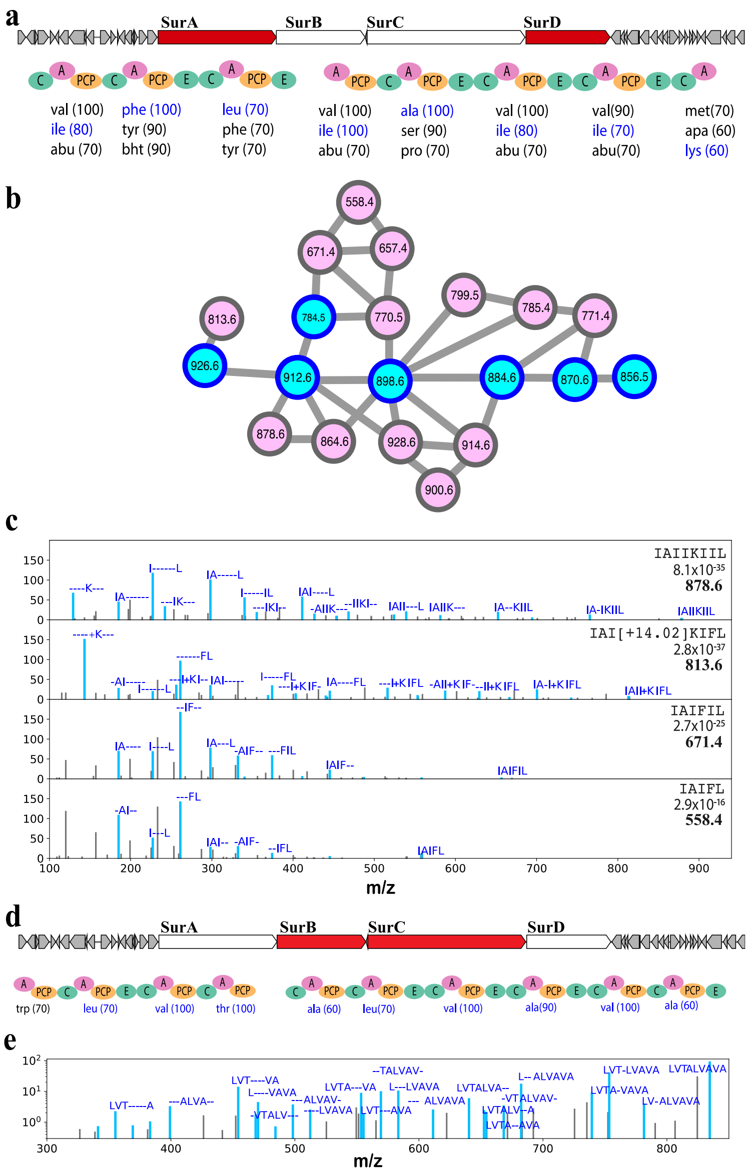

Supplementary Figure 2. Known and novel surugamide variants identified by NRPminer in the SoilActi dataset. Suragamide BGC contains four successive genes, namely SurA, SurB, SurC, and SurD with five, four, six, and three A-domains, respectively. SurA and SurD synthesize cyclic surugamides A-D using a non-canonical assembly line, while SurB and SurC synthesize a linear surugamide F. (**a**) Surugamide BGC from *S. albus* with SurA and SurD highlighted in red, while SurB and SurC are shown in white. In the middle, A-, C-, PCP-, and E-domains appearing in the corresponding NRPS are shown. Three highest-scoring amino acids for each A-domain in this NRPS (according to NRPSpredictor2[3] predictions) are shown below the corresponding A-domains. Amino acids appearing in surugamide A (IFLIAIIK) are shown in blue. (**b**) Spectral network formed by spectra that originated from cyclic surugamides (corresponding to the NRPS shown in part **a**) including the seven known cyclic surugamides. The known cyclic surugamides are shown in blue, while the purples nodes represent the novel cyclic variants identified by NRPminer. (**c**) NRPminer predicted novel cyclic surugamides with eight, seven, six, and five amino acids. For each length, the annotated spectrum representing the lowest p-value (among all PSMs corresponding to the identified novel surugamides with that length) is

presented. Amino acid sequence, p-value, and precursor mass of each PSM is shown in the top right corner. The p-values are computed based on MCMC approach using MS-DPR[4] with 10000 simulations. Annotated peaks are shown in blue. The spectra were annotated based on predicted NRPs IAIIKIIL, IAIKIFL, IAIFIL, IAIFL, from top to bottom. The "+" sign represents the addition of [+14.02Da]. Supplementary Table 8 shows the predicted amino acids and p-values for all NRPs represented by the nodes in part **b**. (**d**) Surugamide BGC from *S. albus* with SurB and SurC highlighted in red, while SurA and SurD are shown in white. In the middle, A-, C-, PCP-, and E-domains appearing in the corresponding NRPS are shown. The highest-scoring amino acids for each A-domain in this NRPS (according to NRPSpredictor2[3] predictions) are shown below the corresponding A-domains. Amino acids appearing in the novel surugamide G (LVTALVAVA) are shown in blue. The amino acid shown in black did not appear in the predicted surugamide G. (**e**) Annotated spectrum representing the novel surugamide G (synthetized by the NRPS shown in part **d**) with the lowest p-value among all spectra representing this NRP (p-value=5.0×10^{-46}). Annotated peaks are shown in blue.

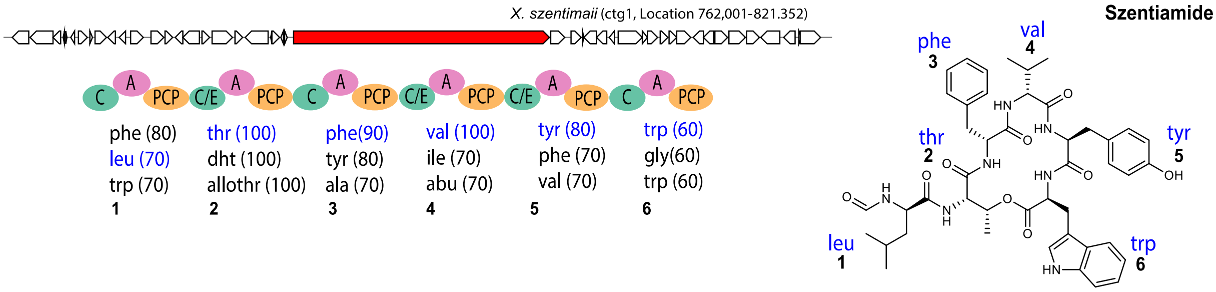

Supplementary Figure 3. Szentiamide biosynthetic gene clusters. (**Left**) szentiamide BGC in *Xenorhabdus szentirmaii* DSM 16338 with NRPS genes (shown in red) which is consistent with the previous study[5]. Three highest scoring NRPSpredictor2[3] amino acid predictions for each A-domain in these BGC are shown. Amino acids corresponding to the correct structure are shown in blue. NRPminer identified this NRP with p-value 7.0×10^{-31}. The p-values are computed based on MCMC approach using MS-DPR[4] with 10000 simulations. (**Right**) The structure of the szentiamide is shown with amino acids highlighted in blue.

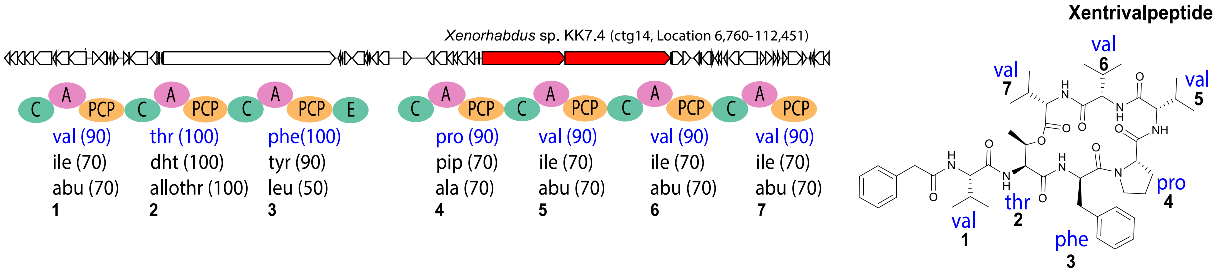

Supplementary Figure 4. Predicted xentrivalpeptide biosynthetic gene clusters. (**Left**) The BGC in *Xenorhabdus* sp. KK7.4 predicted to encode xentrivalpeptide with NRPS genes (shown in red). Three highest scoring NRPSpredictor2[3] amino acid predictions for each A-domain in these BGCs are shown. Amino acids corresponding to the correct structure are shown in blue. NRPminer identified this NRP with p-value 6.4×10^{-37}. The p-values are computed based on MCMC approach using MS-DPR[4] with 10000 simulations. (**Right**) The structure of xentrivalpeptide is shown with amino acids highlighted in blue.

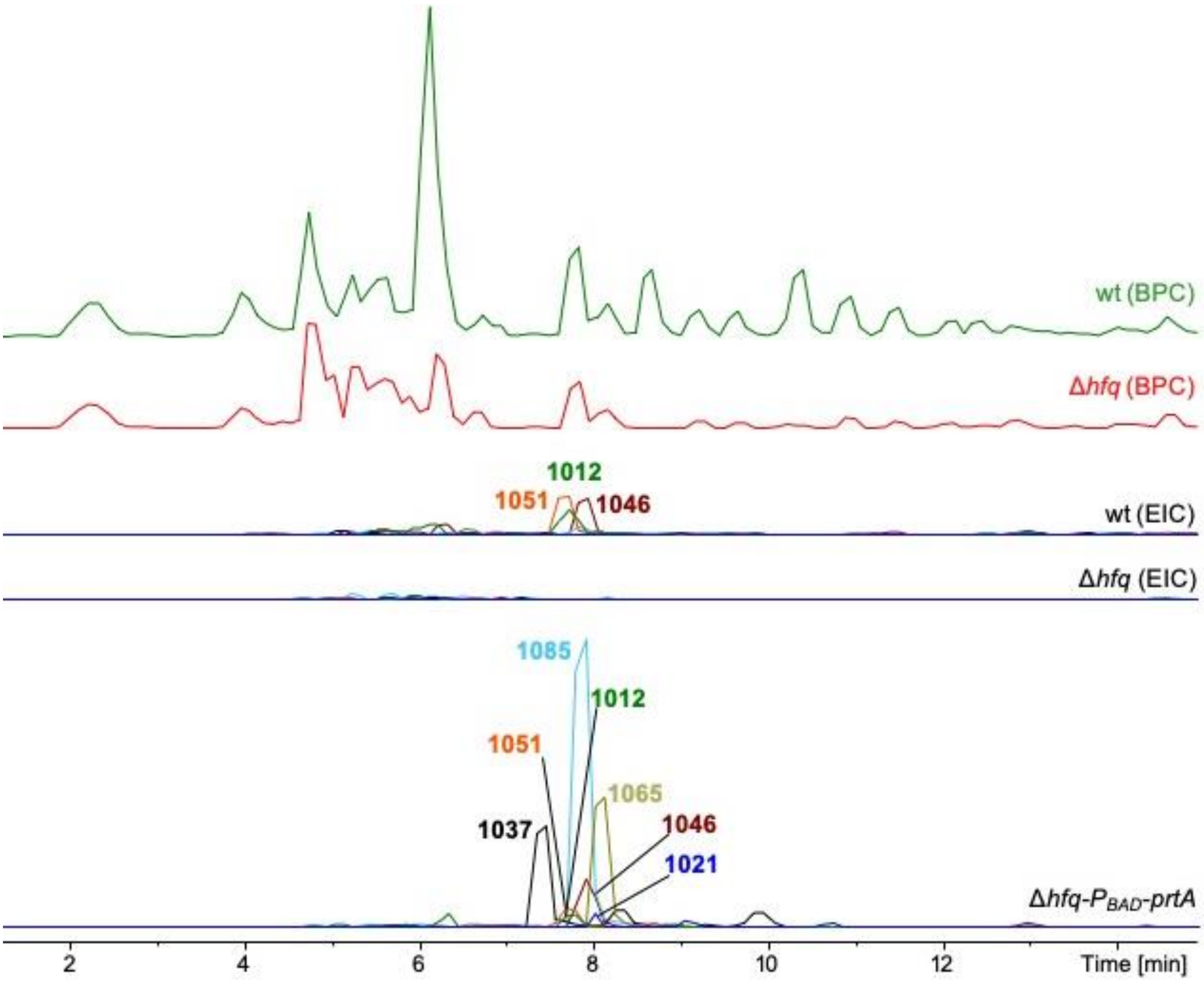

Supplementary Figure 5. (Top) Base peak chromatogram (BPC) of *X. doucetiae* wt (green) and *X. doucetiae*-Δ*hfq* (red) crude extracts**. (Bottom)** Extracted ion chromatograms (EIC) of PRT derivatives from the extract of induced *X. doucetiae*-Δ*hfq*-P$_{BAD}$-*prtA*.

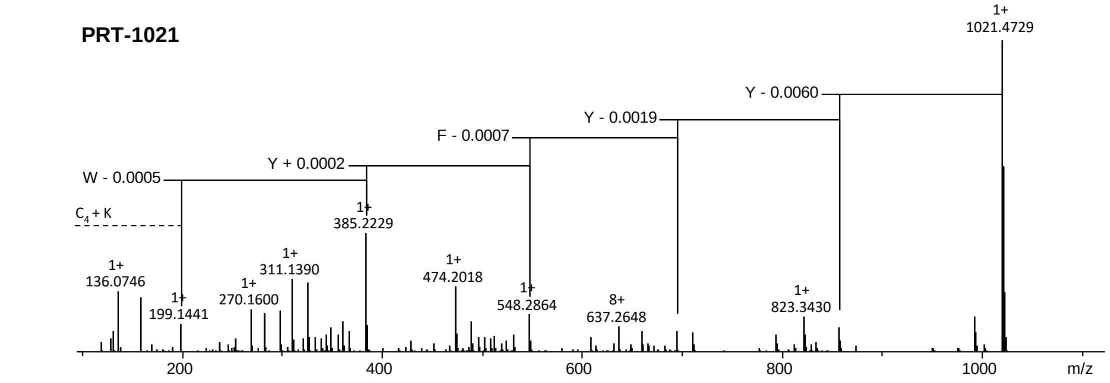
PRT-1021
1+ 1021.4729
Y - 0.0060
Y - 0.0019
F - 0.0007
Y + 0.0002
W - 0.0005
C4 + K
1+ 385.2229
1+ 311.1390
1+ 136.0746
1+ 270.1600
1+ 199.1441
1+ 474.2018
1+ 548.2864
8+ 637.2648
1+ 823.3430
200
400
600
800
1000
m/z

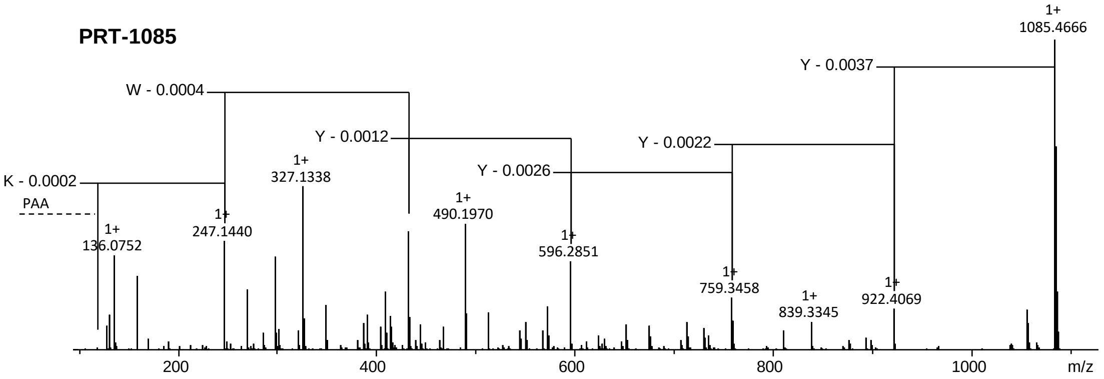
PRT-1085
1+ 1085.4666
Y - 0.0037
W - 0.0004
Y - 0.0012
Y - 0.0022
K - 0.0002
Y - 0.0026
PAA
1+ 327.1338
1+ 490.1970
1+ 136.0752
1+ 247.1440
1+ 596.2851
1+ 759.3458
1+ 839.3345
1+ 922.4069
200
400
600
800
1000
m/z

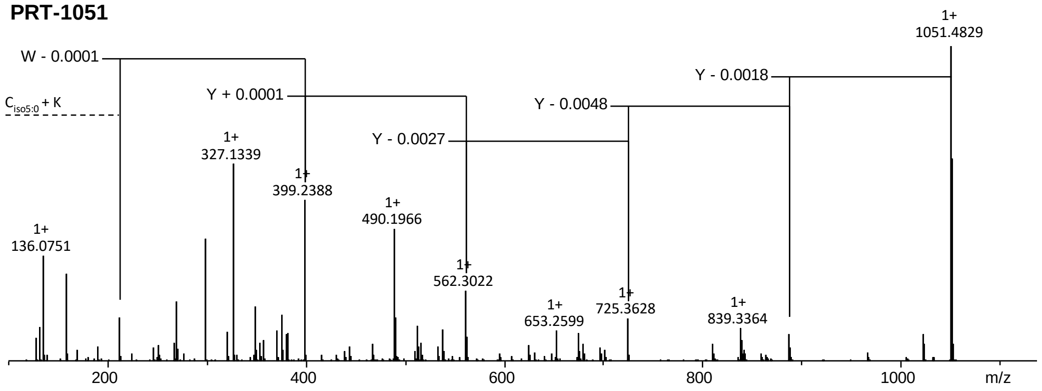
PRT-1051
1+ 1051.4829
W - 0.0001
Y - 0.0018
Y + 0.0001
Y - 0.0048
Ciso5:0 + K
Y - 0.0027
1+ 327.1339
1+ 399.2388
1+ 490.1966
1+ 136.0751
1+ 562.3022
1+ 653.2599
1+ 725.3628
1+ 839.3364
200
400
600
800
1000
m/z

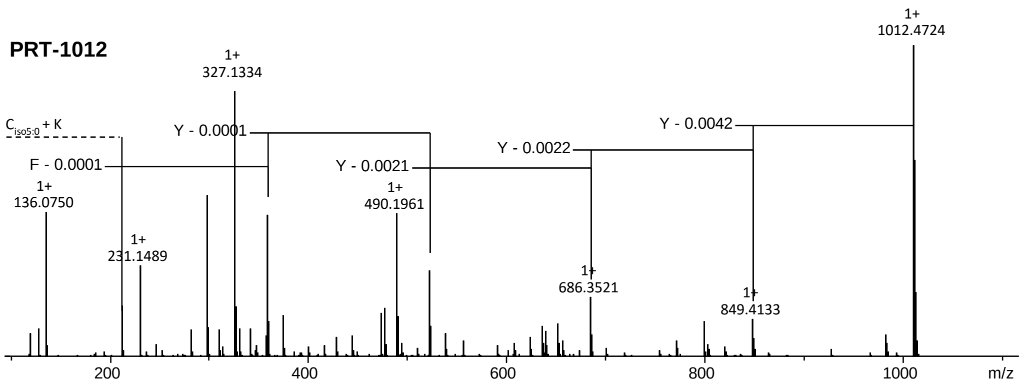
PRT-1012
1+ 1012.4724
1+ 327.1334
Ciso5:0 + K
Y - 0.0001
Y - 0.0042
Y - 0.0022
F - 0.0001
Y - 0.0021
1+ 136.0750
1+ 490.1961
1+ 231.1489
1+ 686.3521
1+ 849.4133
200
400
600
800
1000
m/z

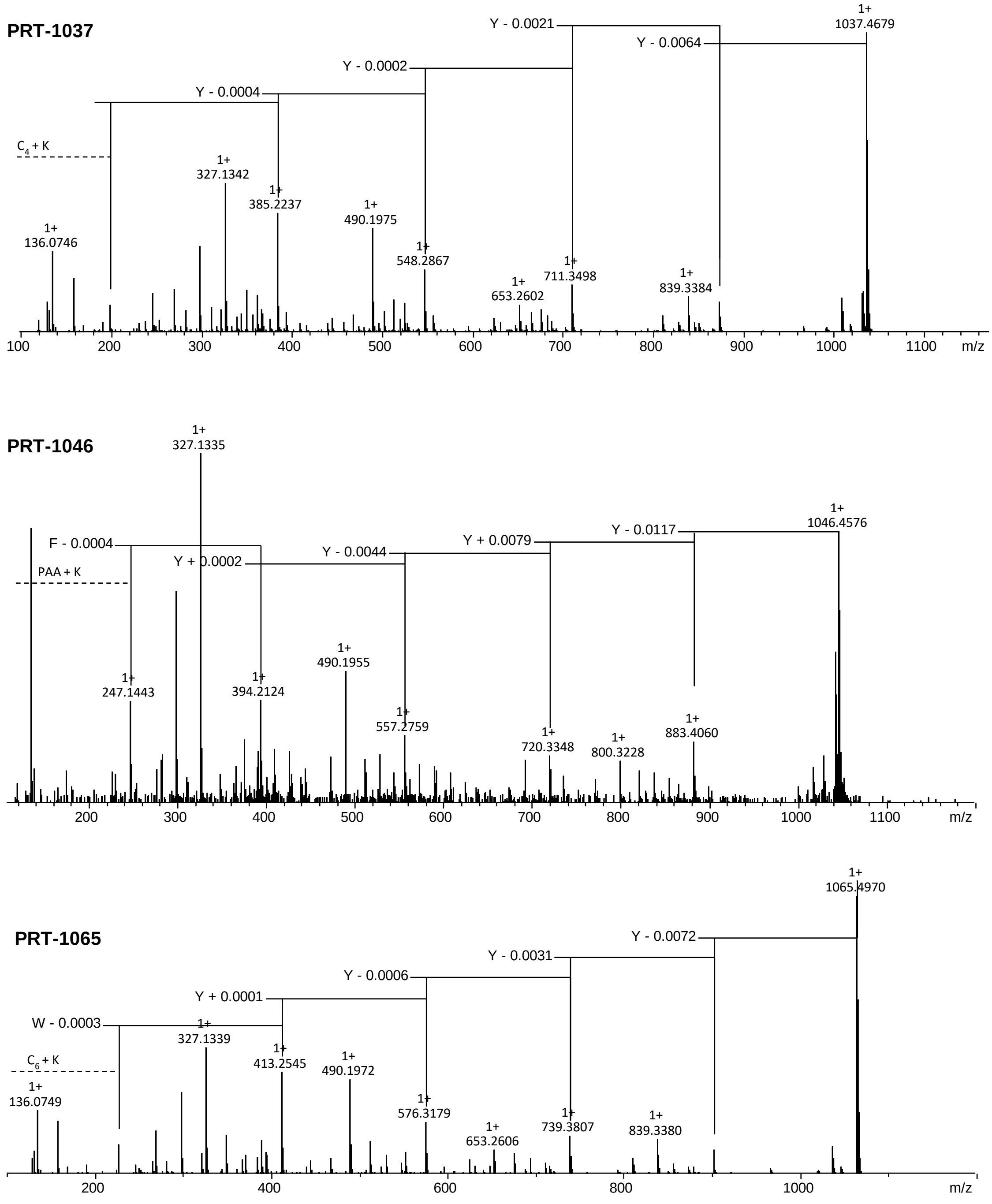

Supplementary Figure 6. Fragmentation pattern (MS/MS of the molecular ions) of selected PRT derivatives from *X. doucetiae* observed by HPLC-MS analysis.

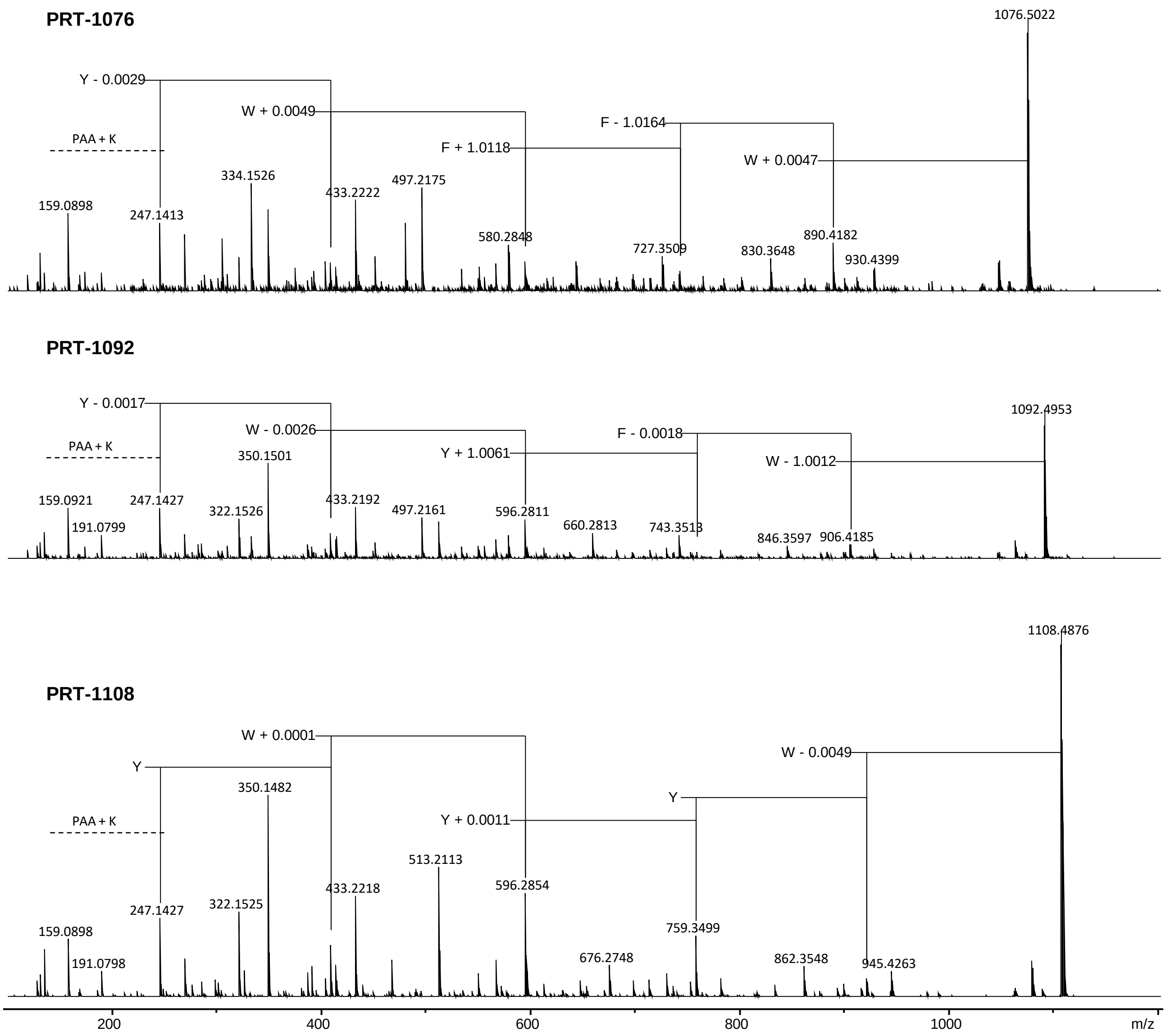

Supplementary Figure 7. Fragmentation pattern (MS/MS of the molecular ions) of selected PRT derivatives from *Xenorhabdus* sp. 30TX1 observed by HPLC-MS analysis.

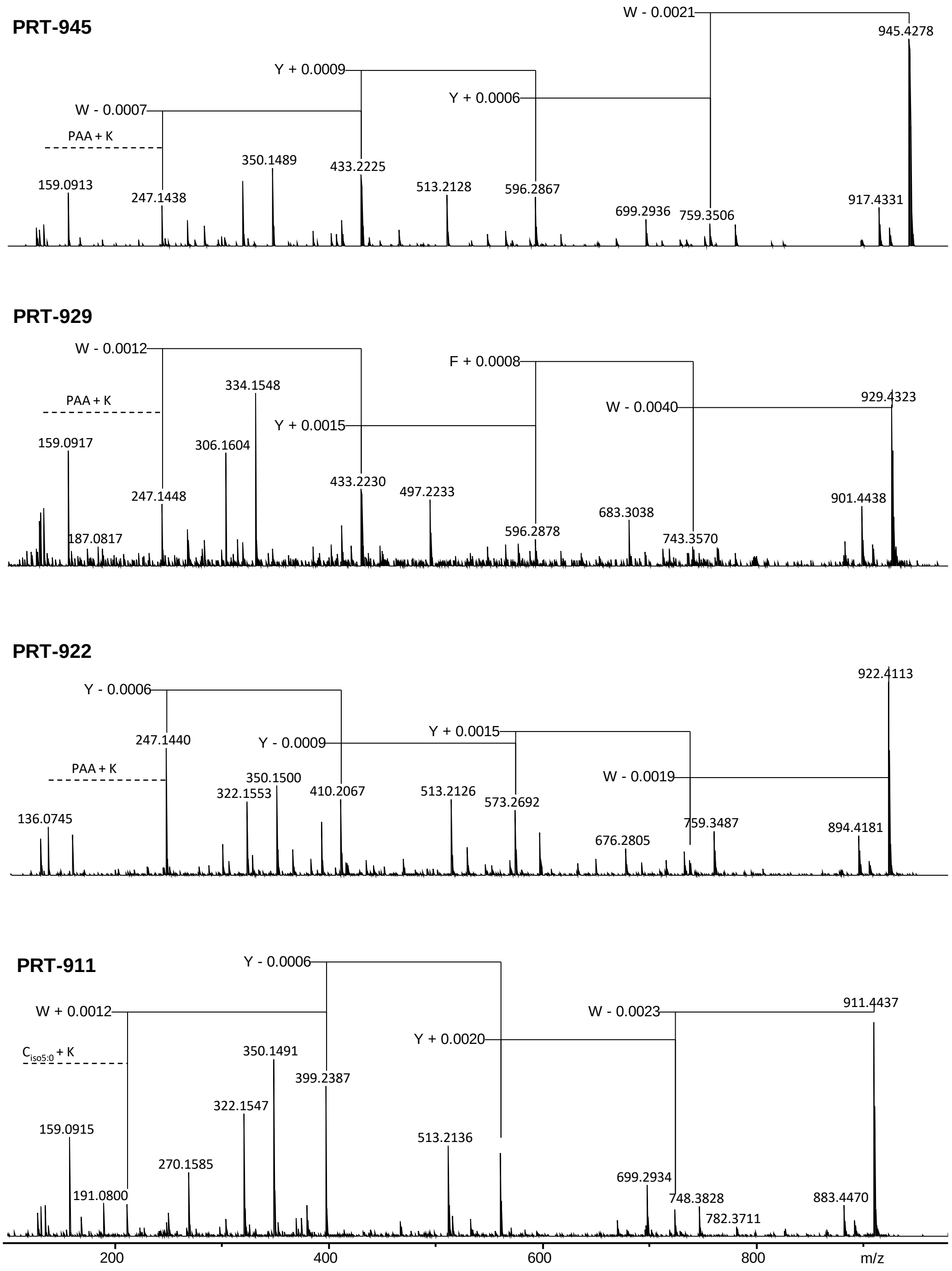

Supplementary Figure 8. **Fragmentation pattern (MS/MS of the molecular ions) of selected PRT derivatives from *X. poinarii* observed by HPLC-MS analysis.**

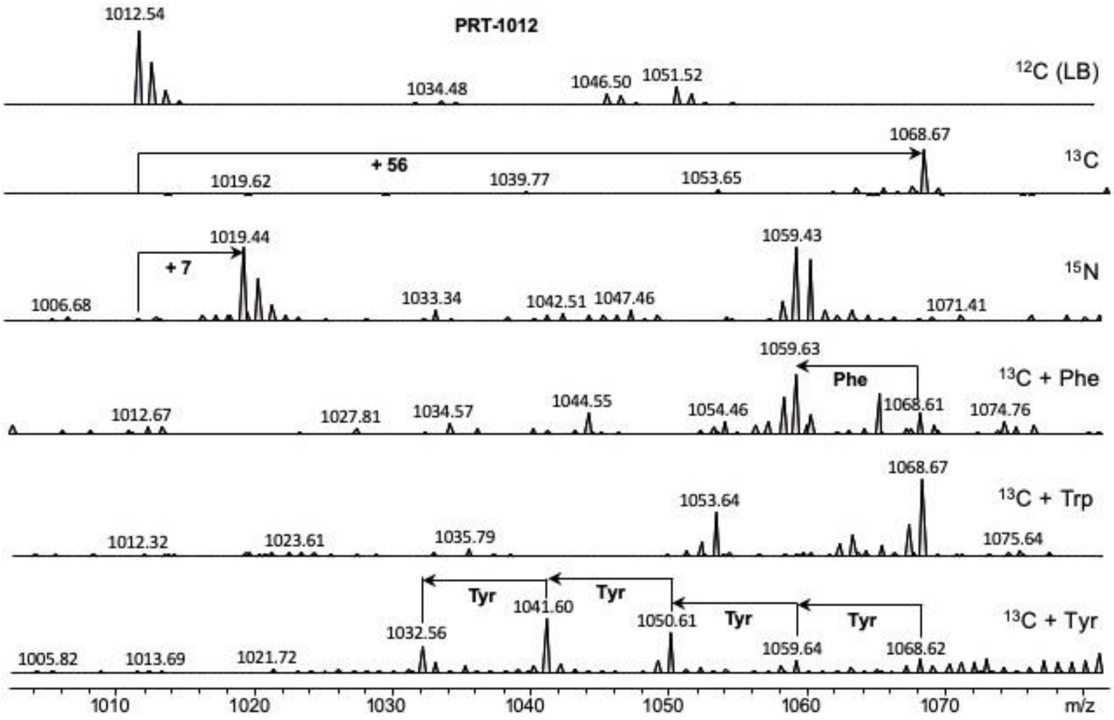
PRT-1012
1012.54
1034.48
1046.50
1051.52
12C (LB)
1068.67
+ 56
1019.62
1039.77
1053.65
13C
1019.44
+ 7
1006.68
1033.34
1042.51
1047.46
1059.43
1071.41
15N
1059.63
Phe
1012.67
1027.81
1034.57
1044.55
1054.46
1068.61
1074.76
13C + Phe
1068.67
1053.64
1012.32
1023.61
1035.79
1075.64
13C + Trp
Tyr
Tyr
Tyr
Tyr
1041.60
1032.56
1050.61
1059.64
1068.62
1005.82
1013.69
1021.72
13C + Tyr
1010
1020
1030
1040
1050
1060
1070
m/z

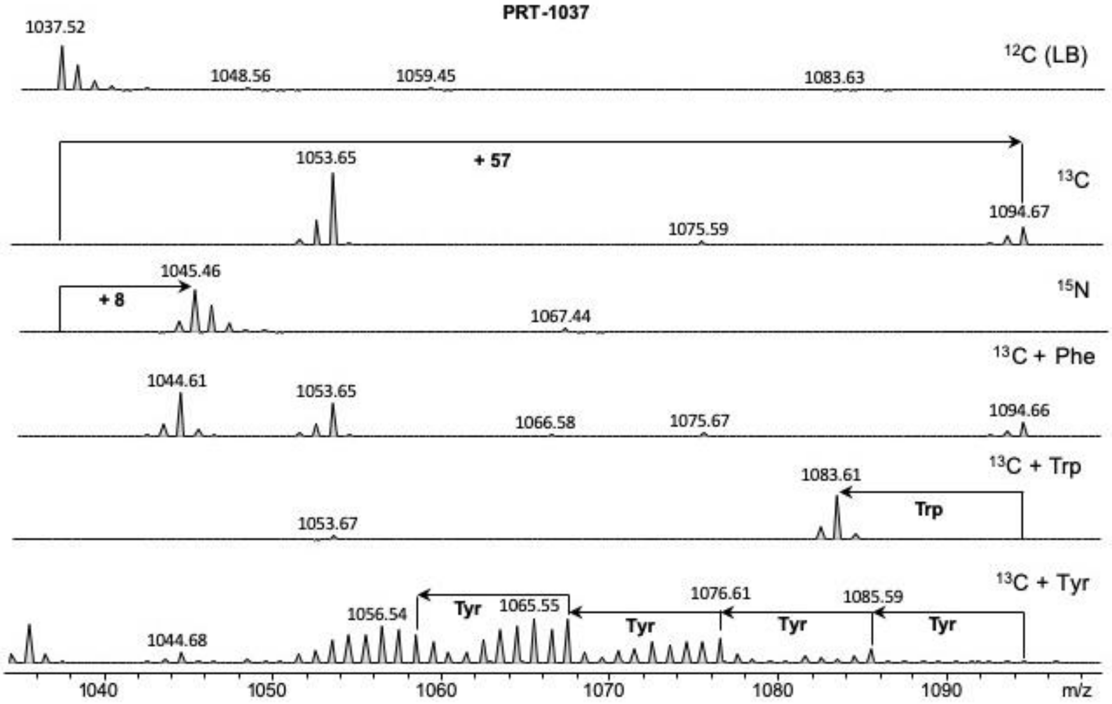
PRT-1037
1037.52
1048.56
1059.45
1083.63
12C (LB)
+ 57
1053.65
13C
1094.67
1075.59
1045.46
+ 8
1067.44
15N
13C + Phe
1044.61
1053.65
1066.58
1075.67
1094.66
13C + Trp
1083.61
Trp
1053.67
13C + Tyr
1076.61
1085.59
1056.54
Tyr
1065.55
Tyr
Tyr
Tyr
1044.68
1040
1050
1060
1070
1080
1090
m/z

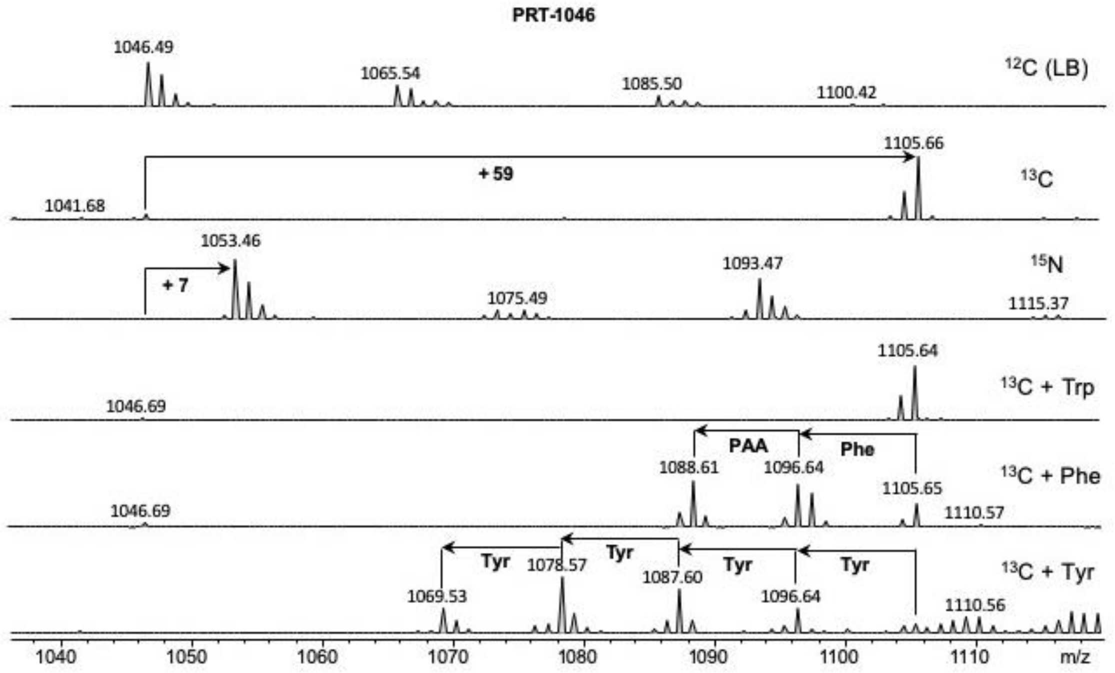

Supplementary Figure 9. MS analysis of selected PRT derivatives after cultivation in ^{12}C (LB), ^{13}C- and ^{15}N- medium. Analysis of the incorporation of non-labelled Phe, Trp, Tyr and Leu added to fully labeled ^{13}C medium.

A

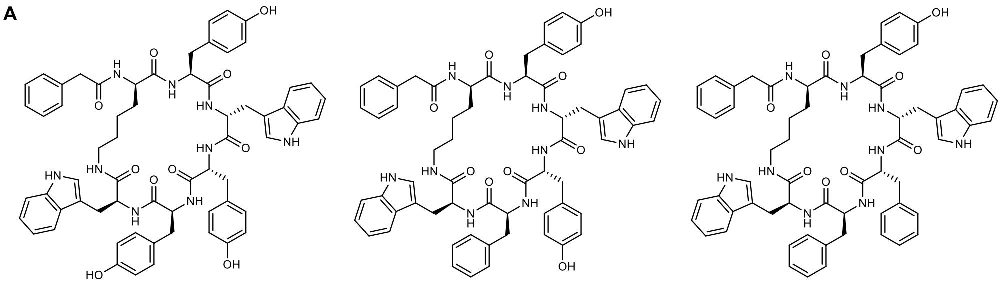

PRT-1108 PRT-1092 PRT-1076

B

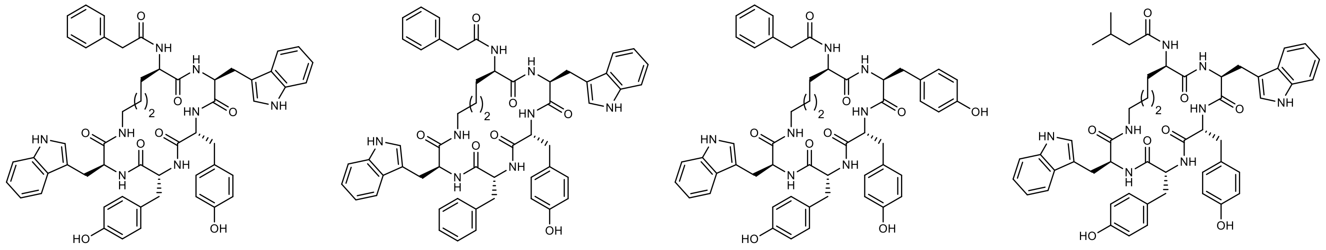

PRT-945 PRT-929 PRT-922 PRT-911

Supplementary Figure 10. (A) Predicted structures of PRT derivatives produced by *Xenorhabdus* sp. 30TX1 including amino acid configuration as found in *X. doucetiae*. **(B)** Predicted structures for PRT derivatives produced by *X. poinarii* including amino acid configuration as concluded from the presence of epimerization domains in the corresponding NRPS PrtAB.

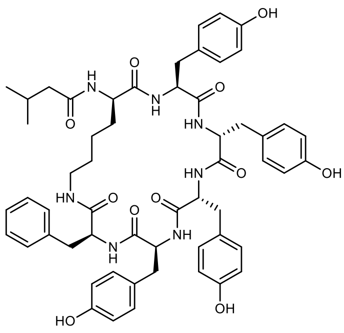

PRT-1012

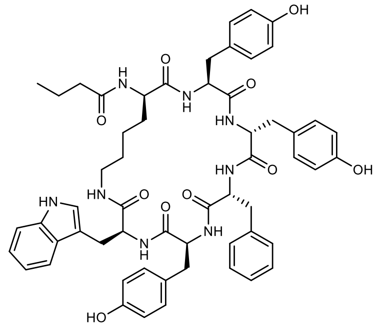

PRT-1021

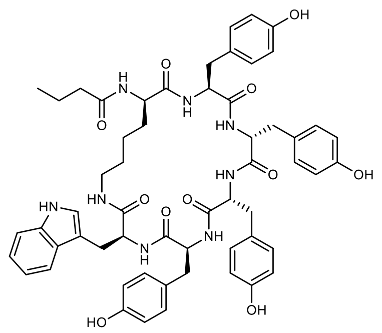

PRT-1037

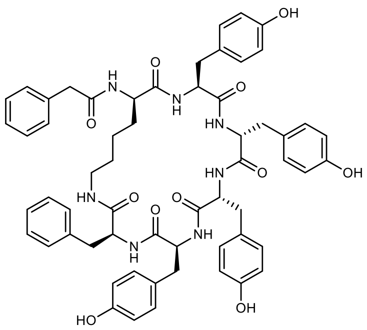

PRT-1046

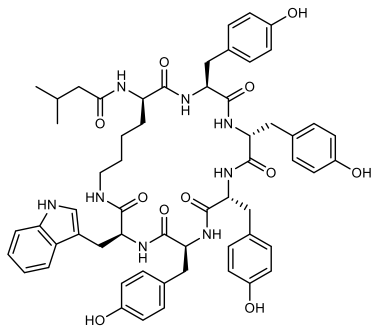

PRT-1051

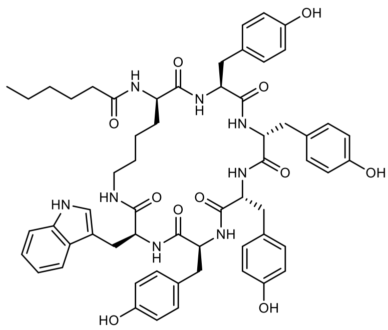

PRT-1065

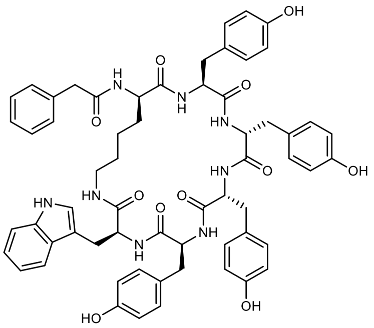

PRT-1085

Supplementary Figure 11. Structures for PRT derivatives produced by *X. doucetiae* including amino acid configuration as concluded from the presence of epimerization domains in the corresponding NRPSs PrtAB.

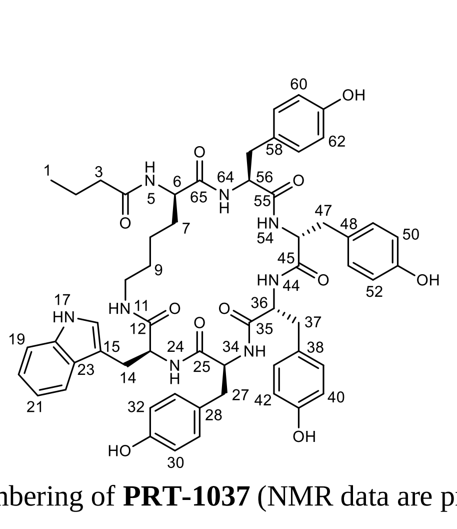

Supplementary Figure 12. Numbering of **PRT-1037** (NMR data are provided in Supplementary Table 2).

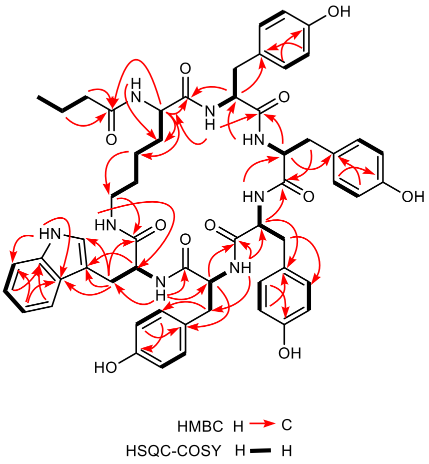

Supplementary Figure 13. Key HMBC and HSQC-COSY correlations **PRT-1037**.

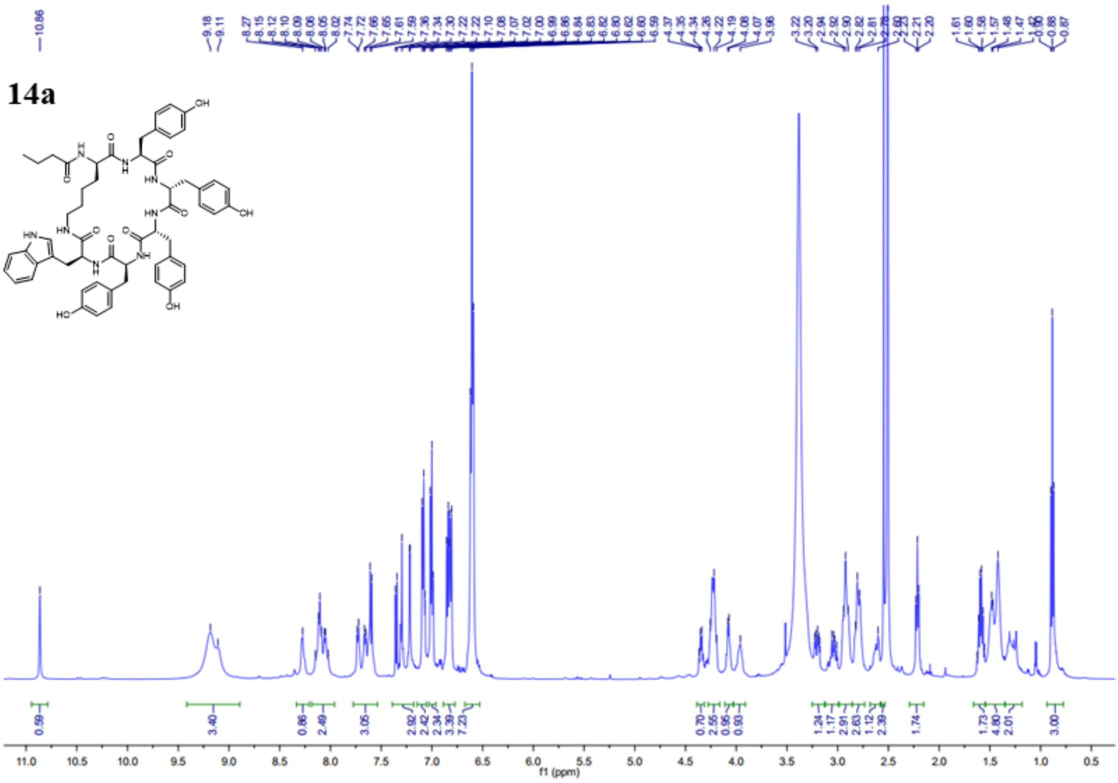

14b

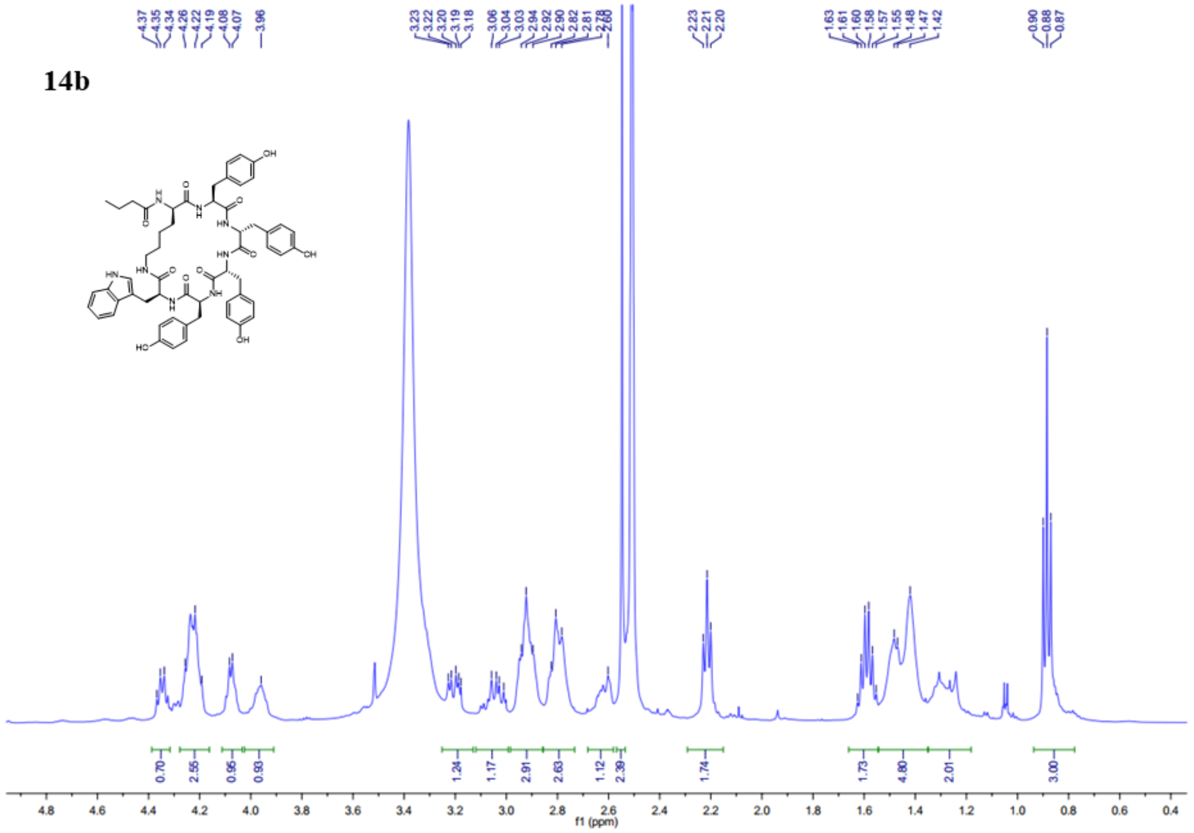

14c

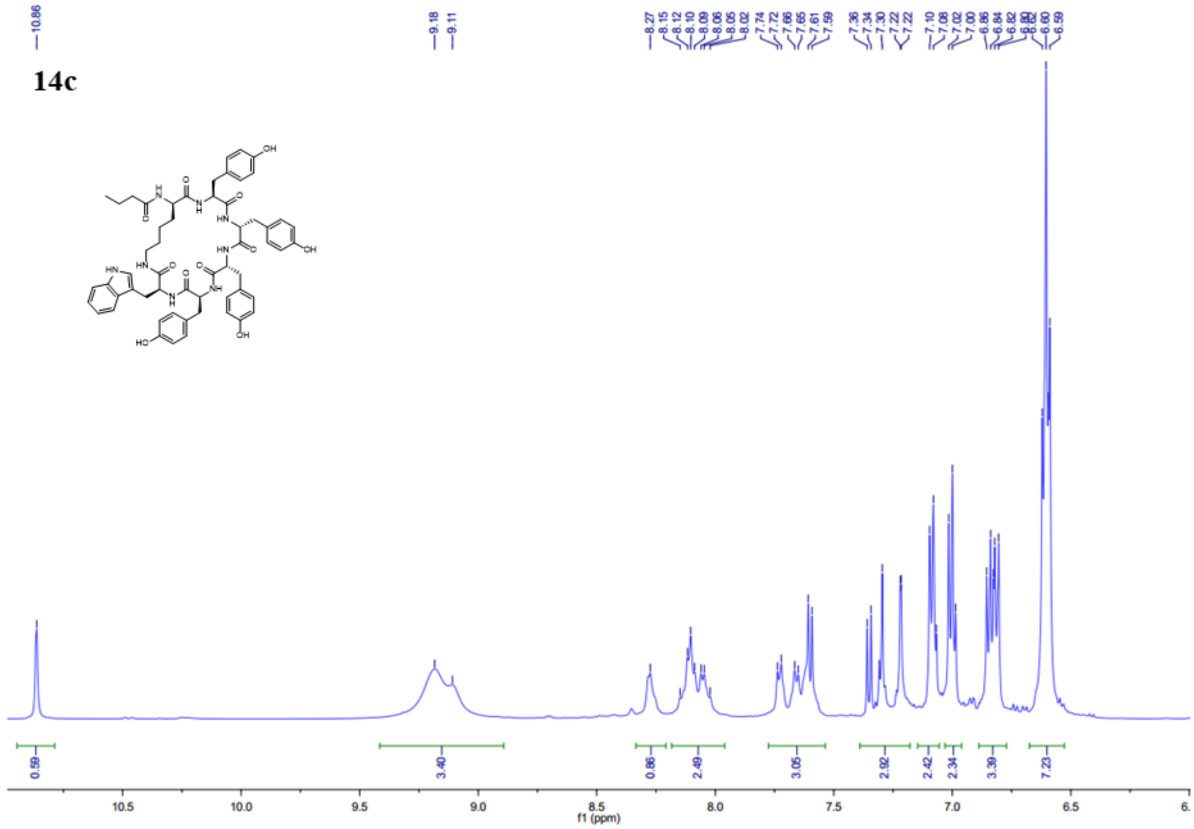

Supplementary Figure 14 (a-c). ^{1}H NMR (500 MHz) spectrum of compound **PRT-1037** in DMSO-d_6.

15a

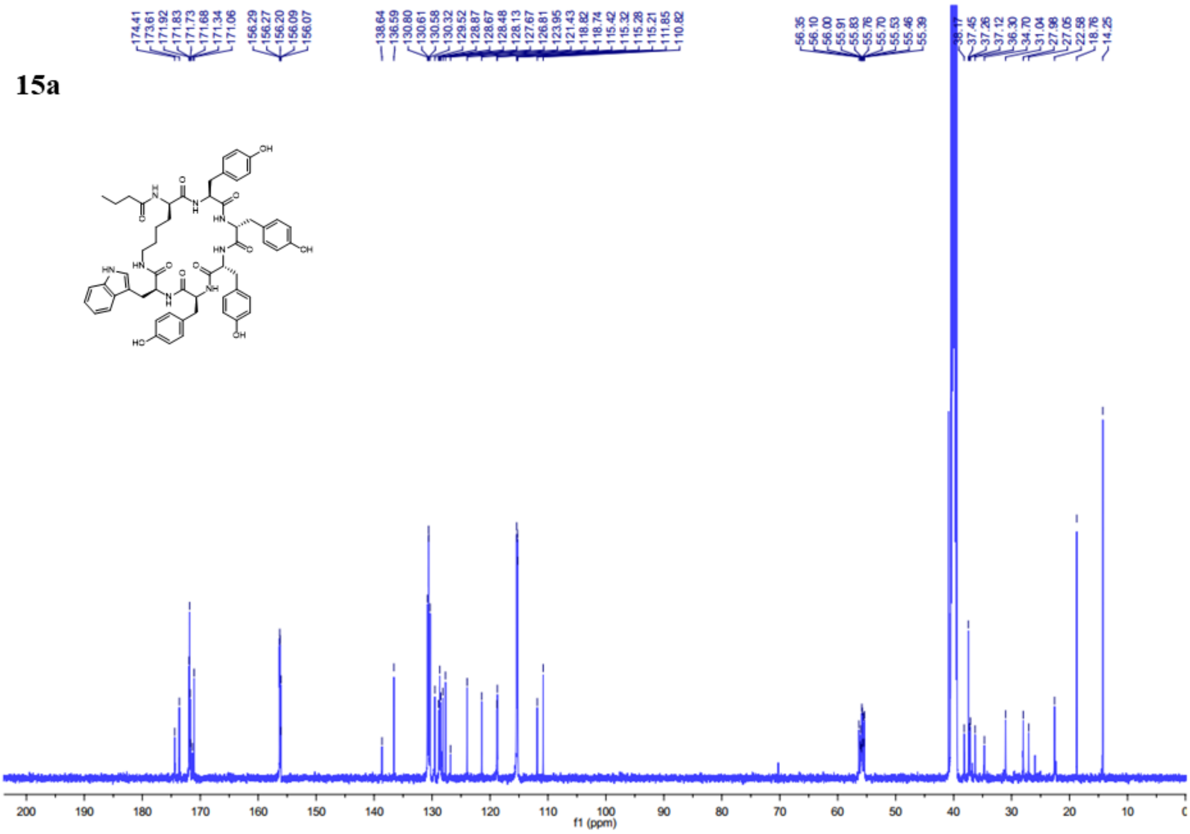

15b

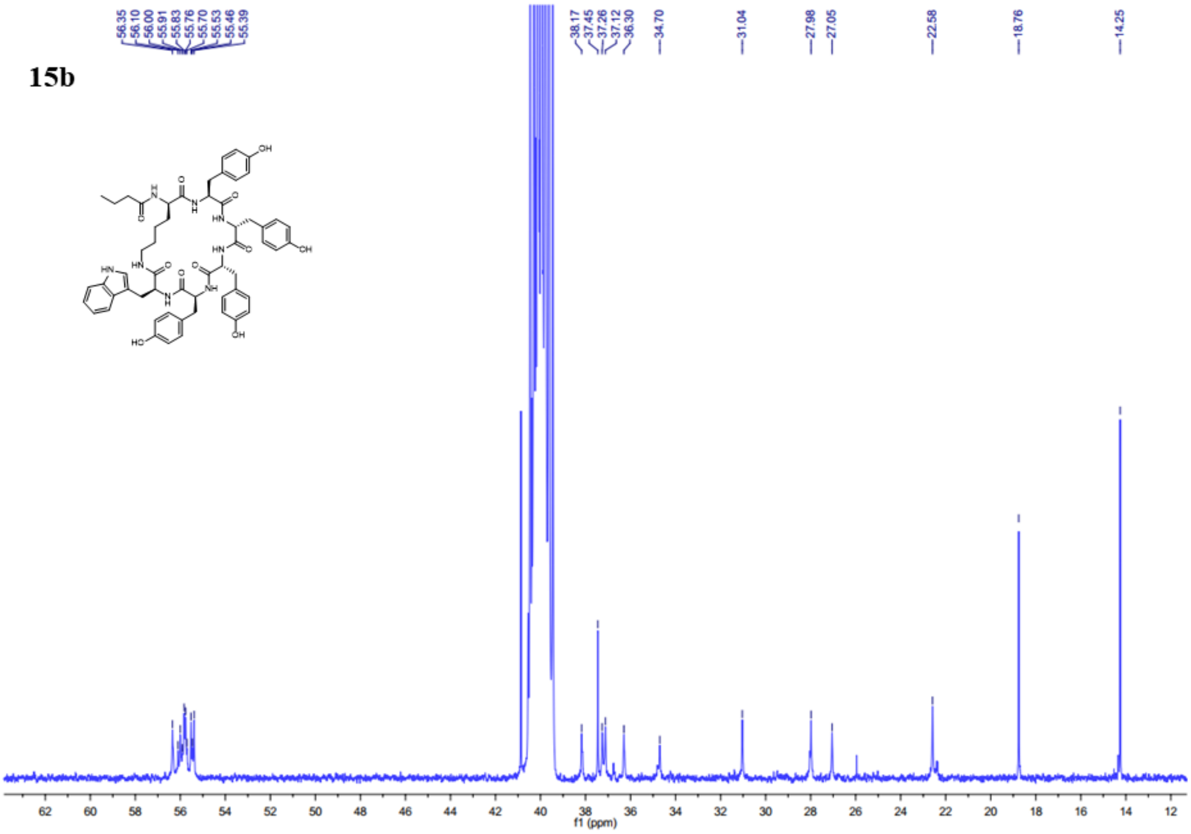

15c

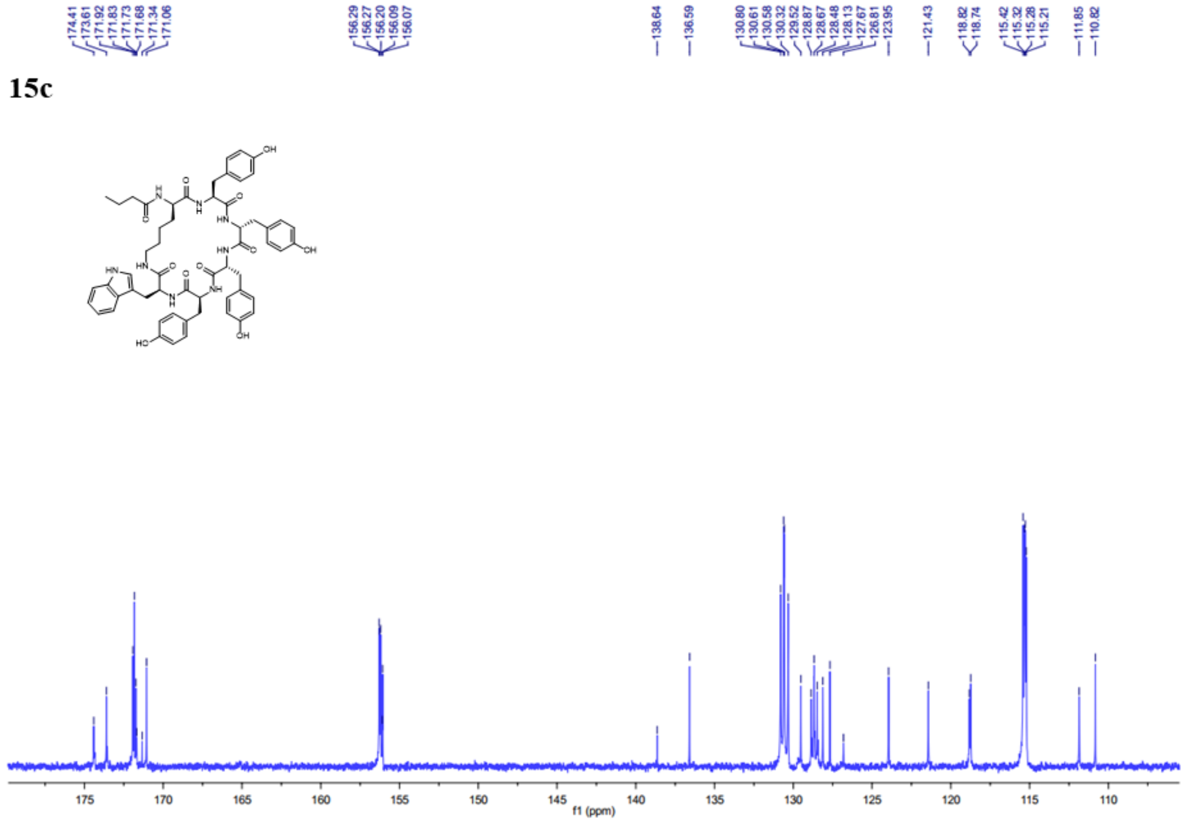

Supplementary Figure 15 (a-c). ^{13}C NMR (125 MHz) spectrum of compound **PRT-1037** in DMSO-d_6.

.

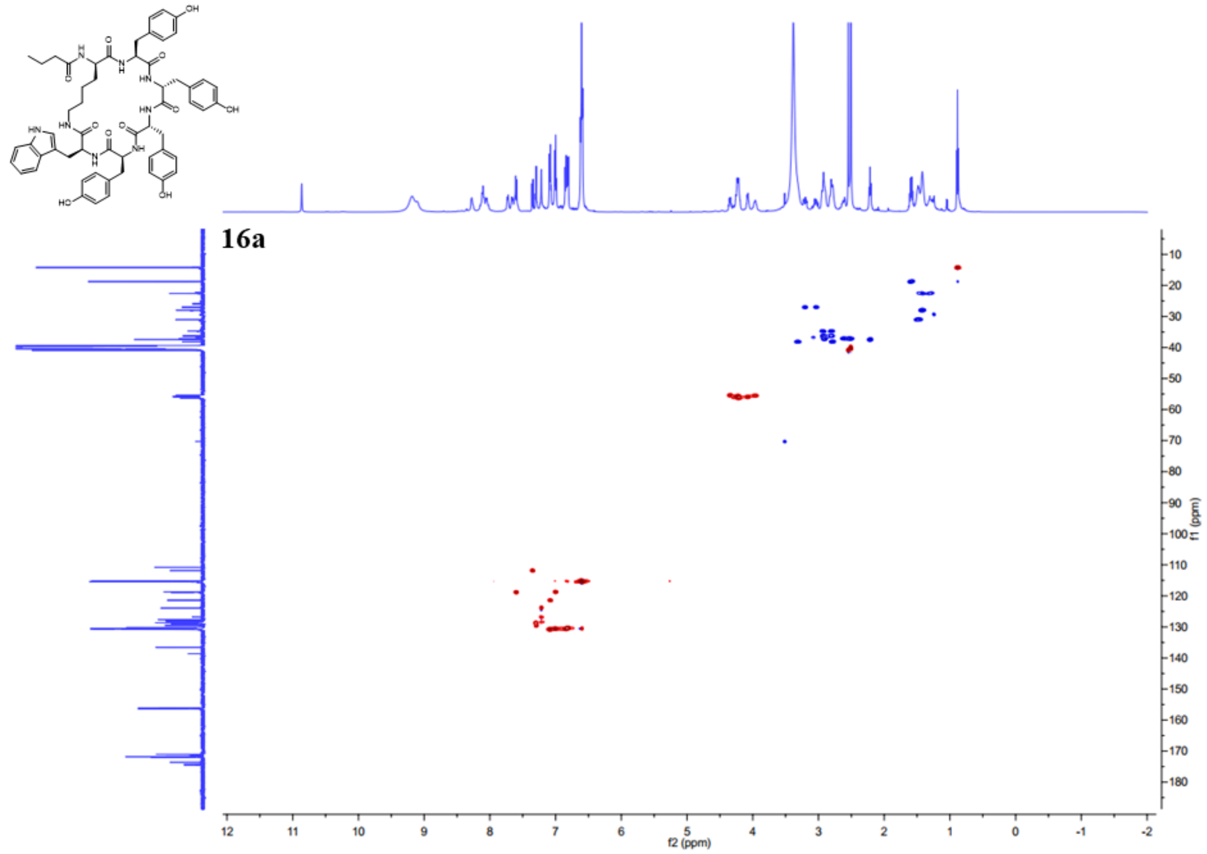

16a

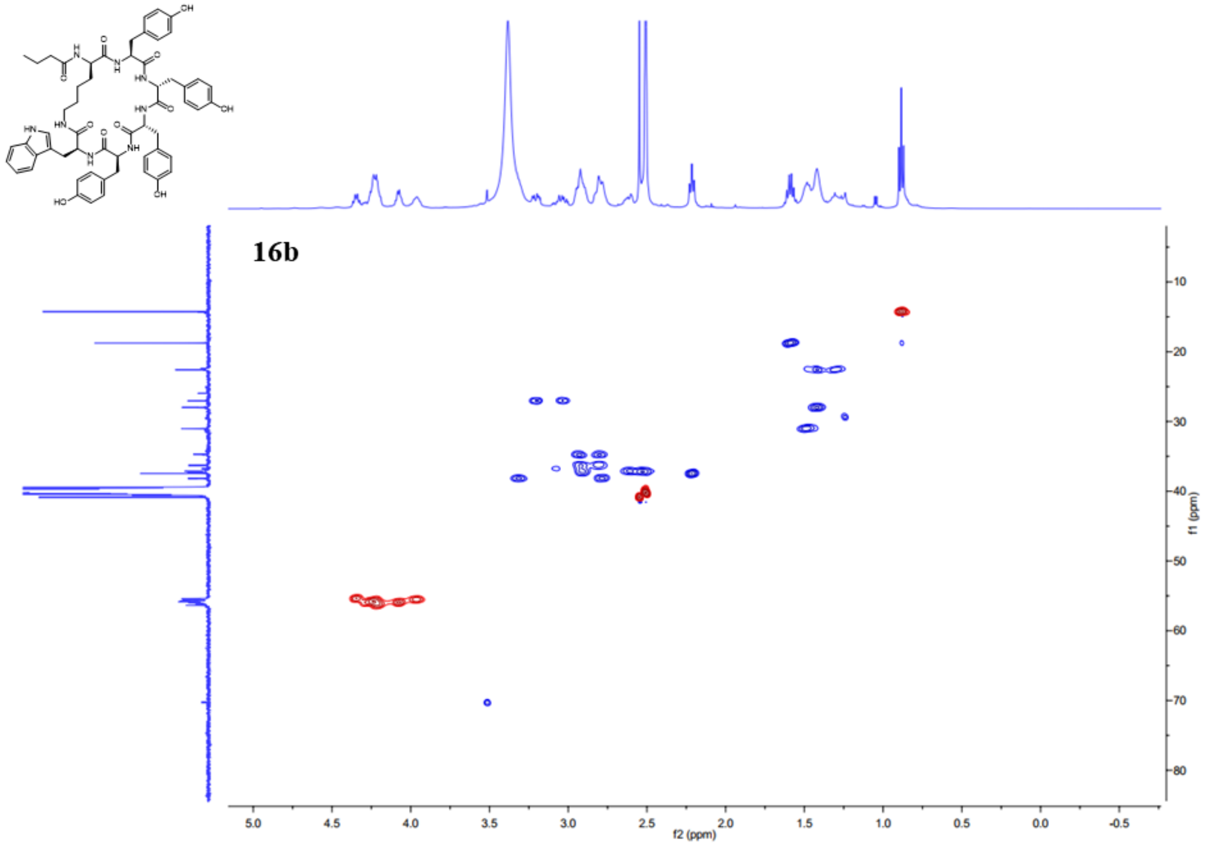

16b

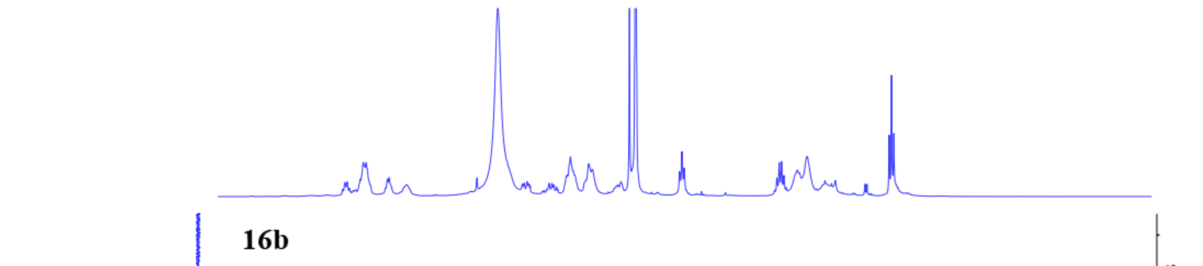

16b

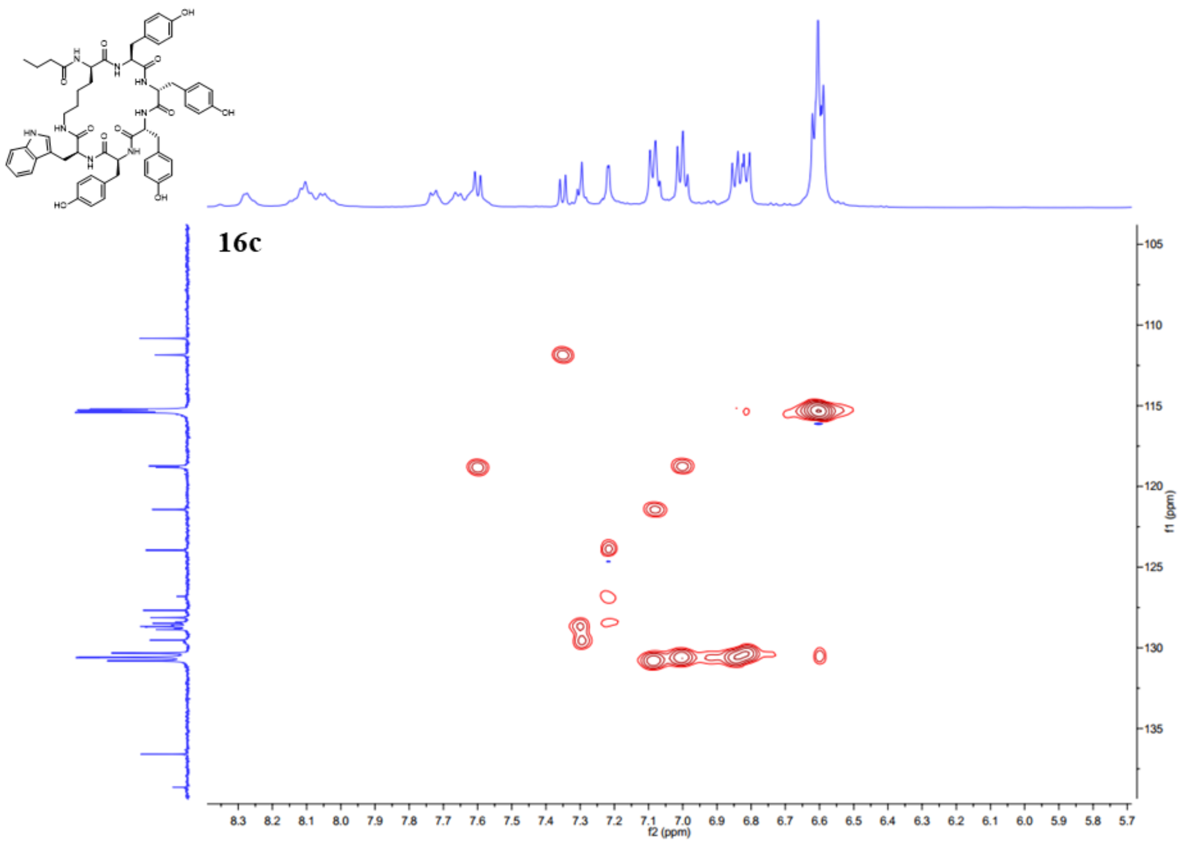

Supplementary Figure 16 (a-c). HSQC (500 MHz) spectrum of compound **PRT-1037** in DMSO-d_6.

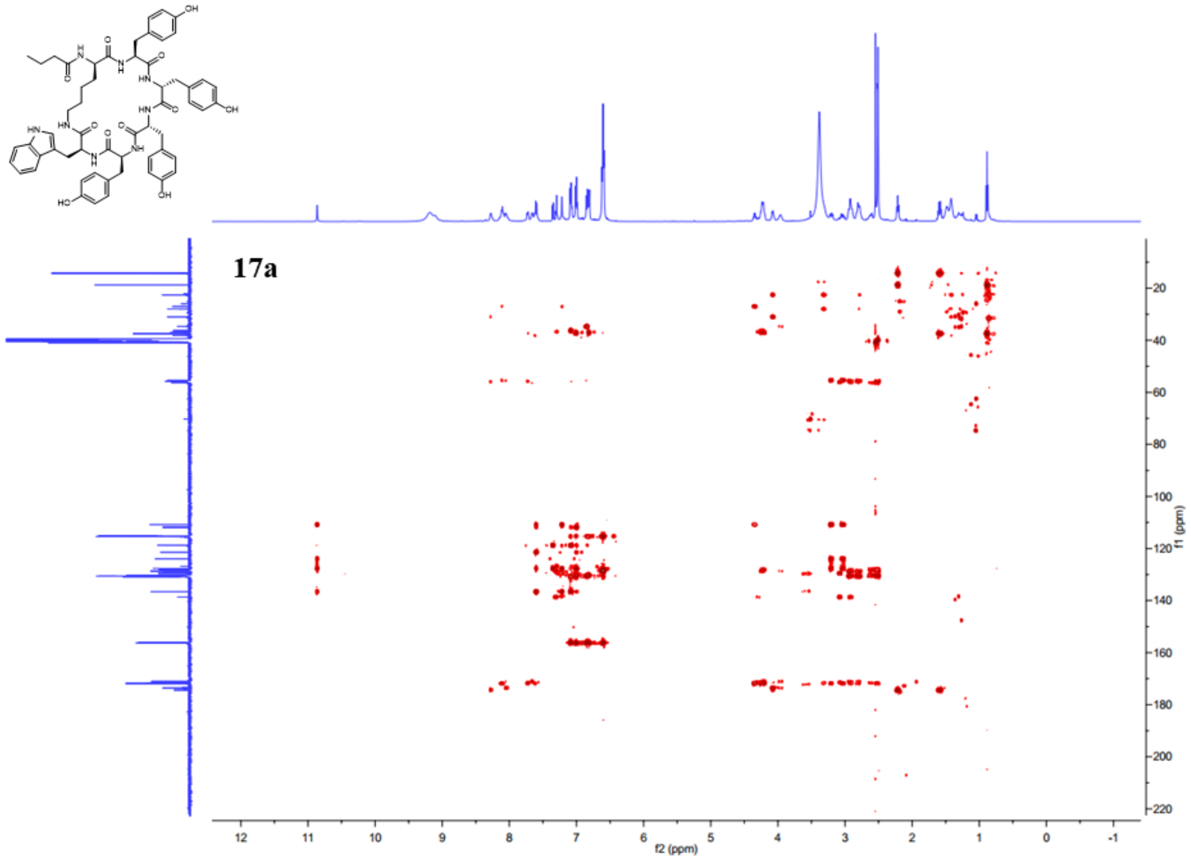
17a
f2 (ppm)
f1 (ppm)

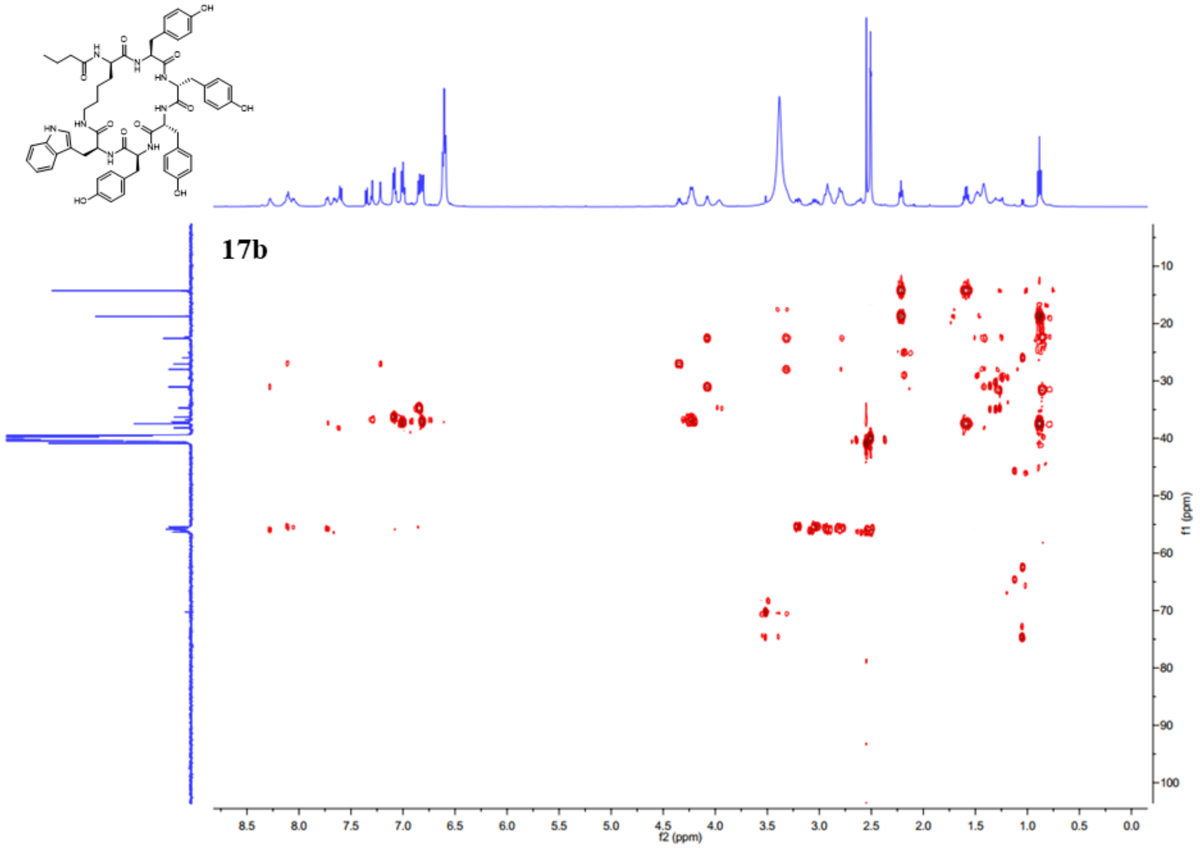
17b
f2 (ppm)
f1 (ppm)

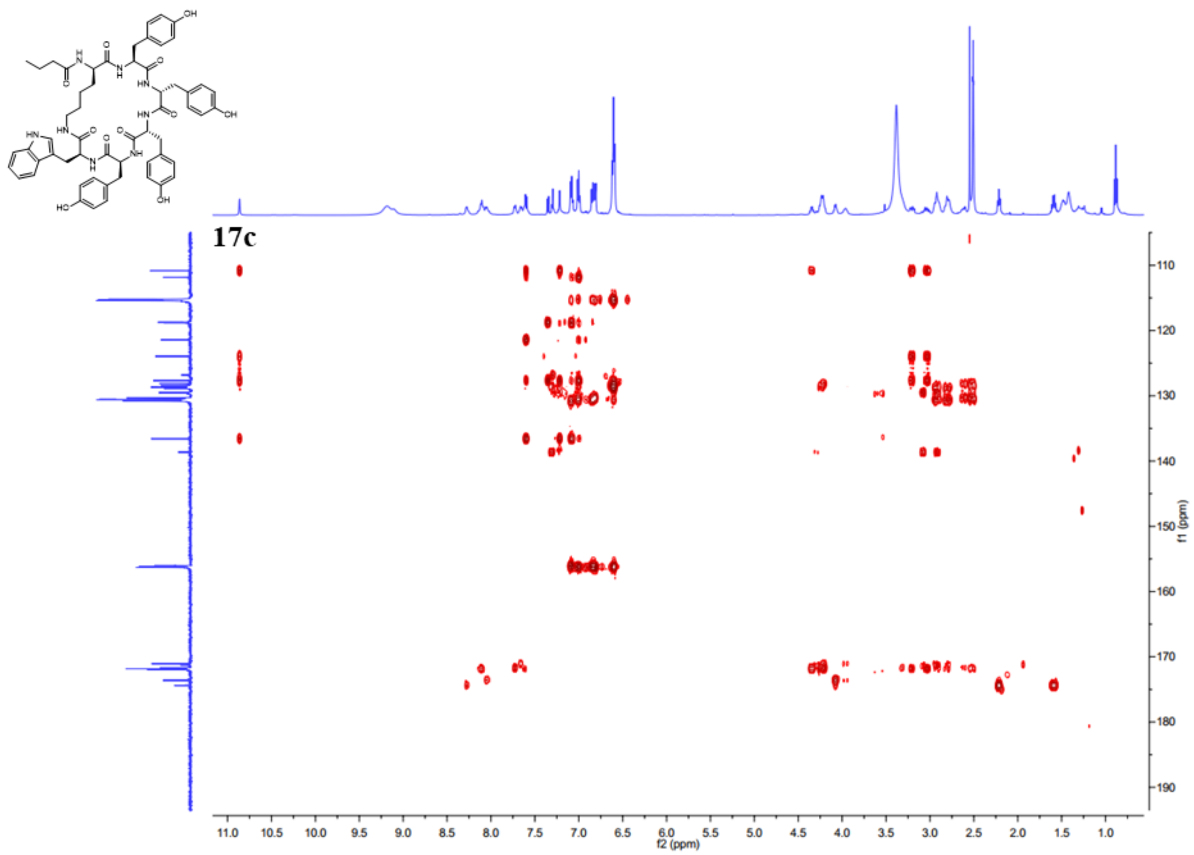

Supplementary Figure 17 (a-c). HMBC (500 MHz) spectrum of compound **PRT-1037** in DMSO-d_6.

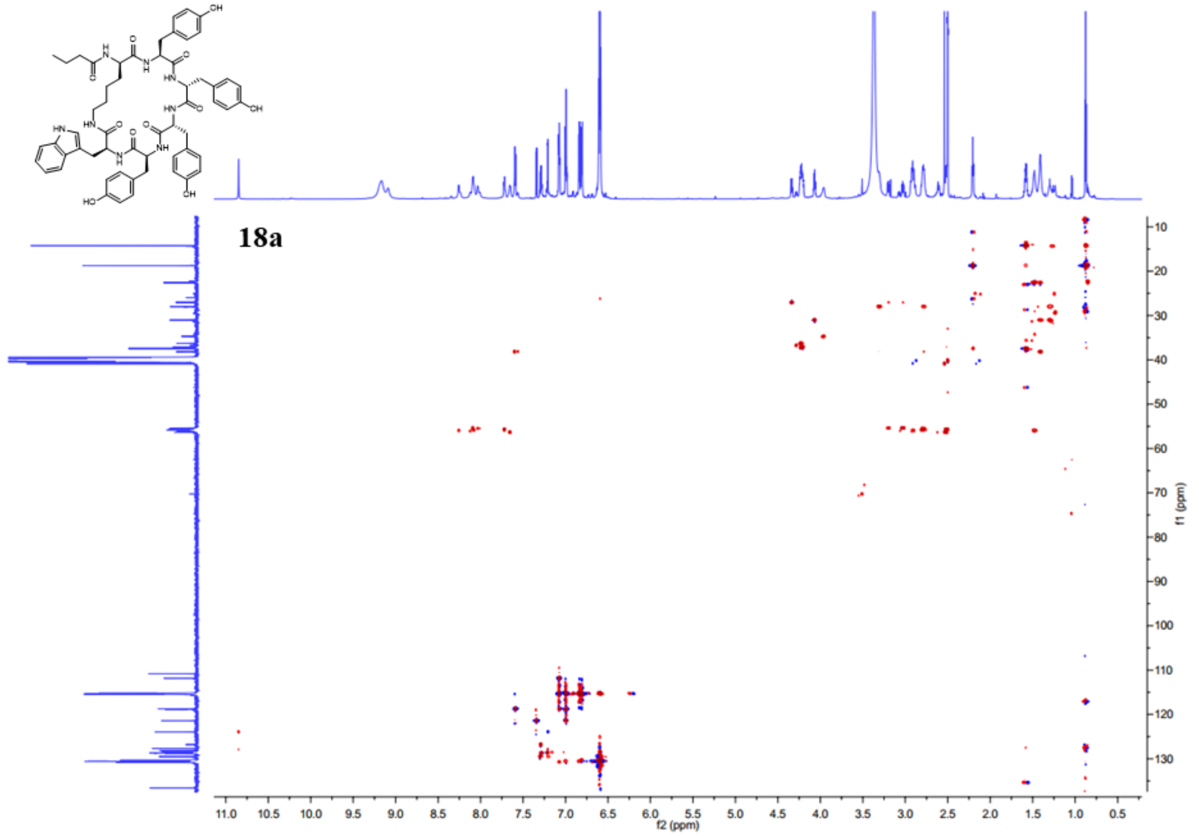
18a
f2 (ppm)
f1 (ppm)

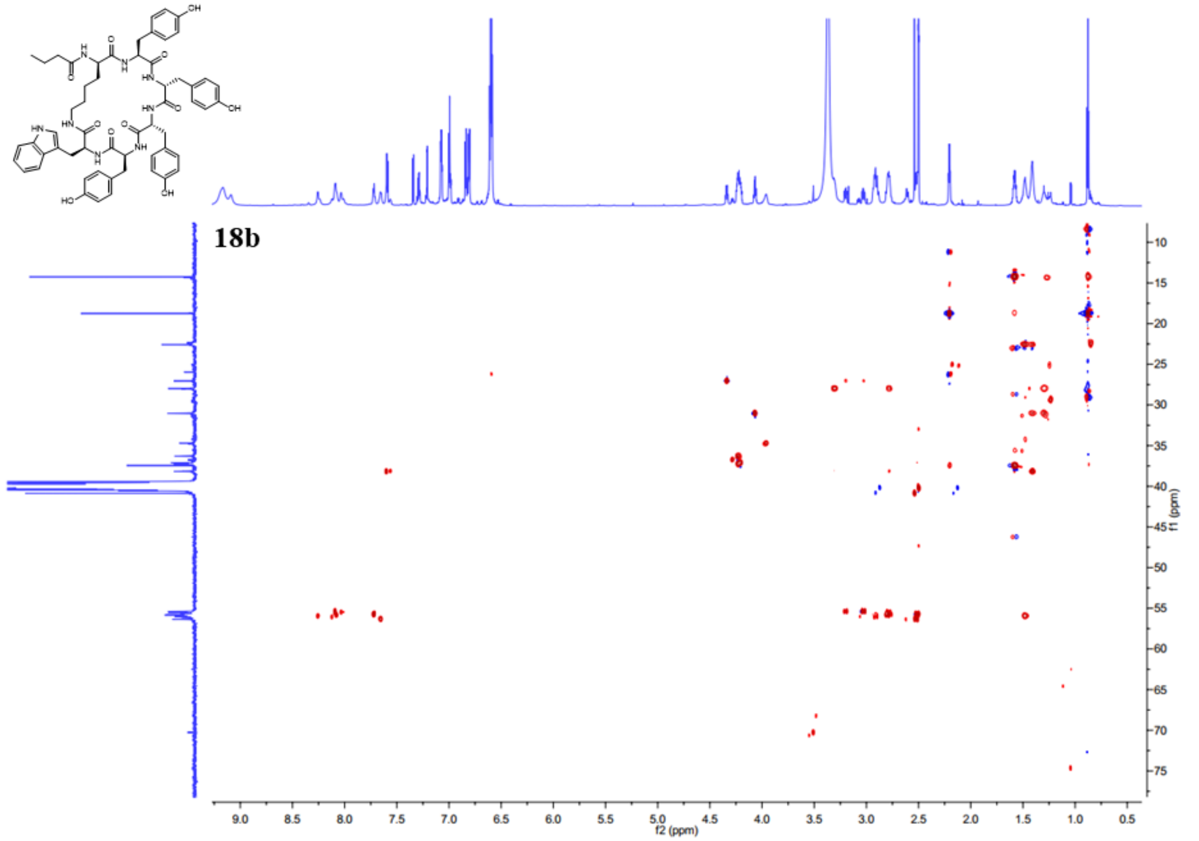
18b
f2 (ppm)
f1 (ppm)

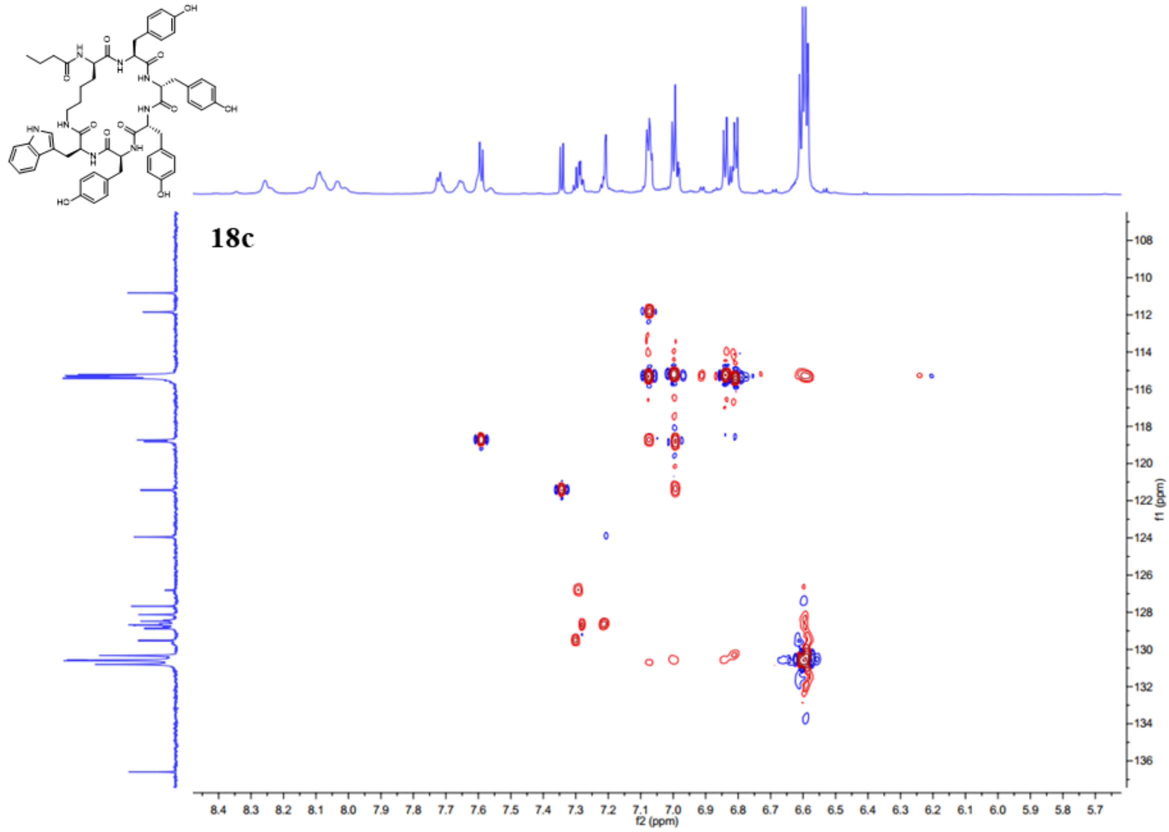

Supplementary Figure 18 (a-c). HSQC-COSY (900 MHz) spectrum of compound **PRT-1037** in DMSO-d_6.

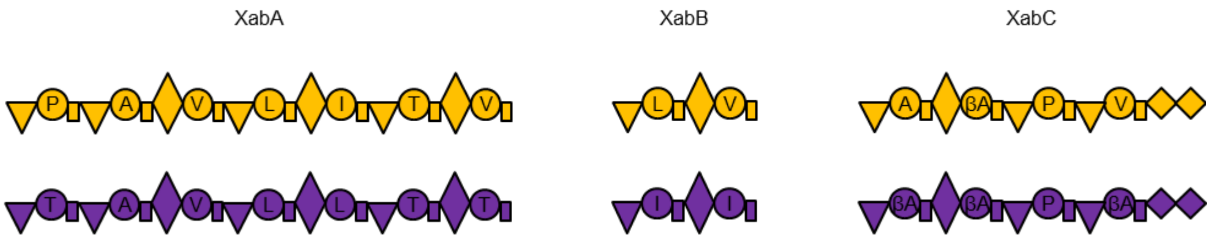

Supplementary Figure 19. General NRPS structure of xenoamicin XabABC in *X. doucetiae* (yellow) and *Xenorhabdus* KJ12.1 (violet). Amino acid specificities are displayed for all A-domains. For domain assignment the following symbols are used: A (large circles), T (rectangle), C (triangle), C/E (diamond), TE-TE (two C-terminal small diamonds).

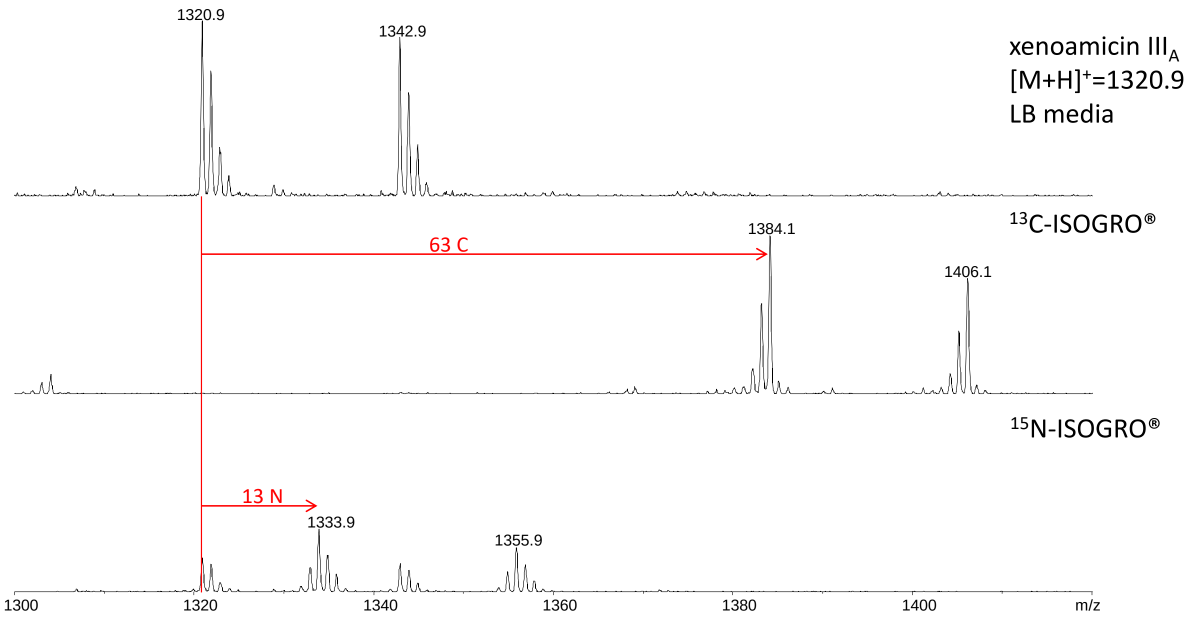

Supplementary Figure 20. Determination of the number of carbon and nitrogen atoms in **XAM-1320** by cultivation of *Xenorhabdus* KJ12.1 in LB medium, ^{13}C labelled or ^{15}N labelled ISOGRO® medium and the following mass shift detected by mass spectrometry.

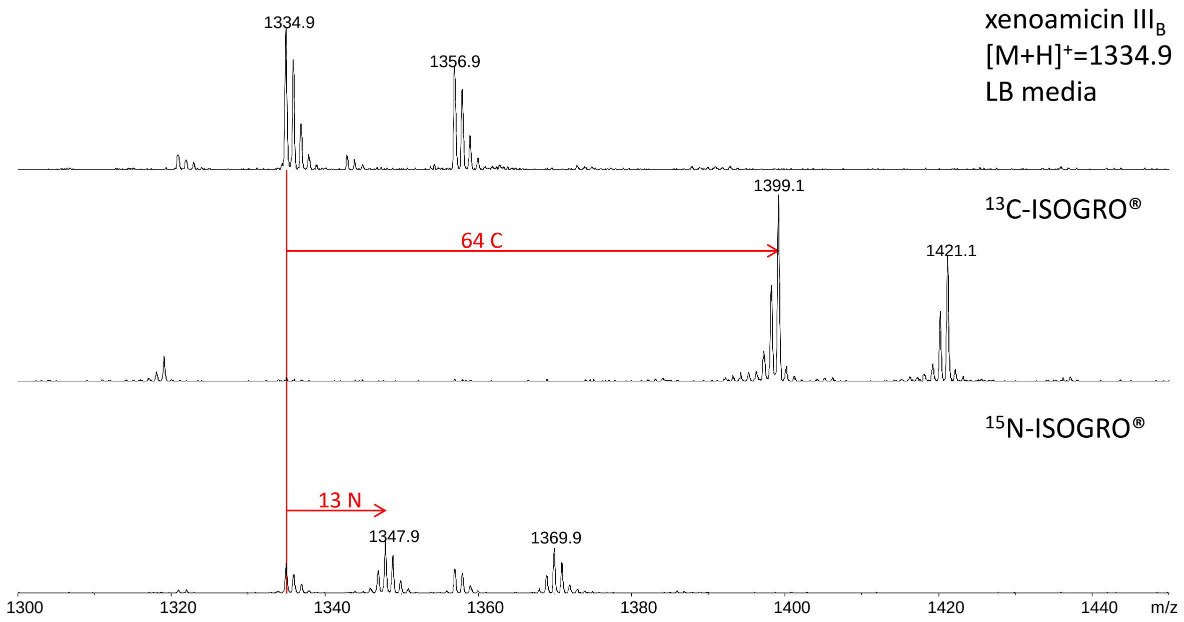

Supplementary Figure 21. Determination of the number of carbon and nitrogen atoms in **XAM-1334** by cultivation of *Xenorhabdus* KJ12.1 in LB medium, ^{13}C labelled or ^{15}N labelled ISOGRO® medium and the following mass shift detected by mass spectrometry.

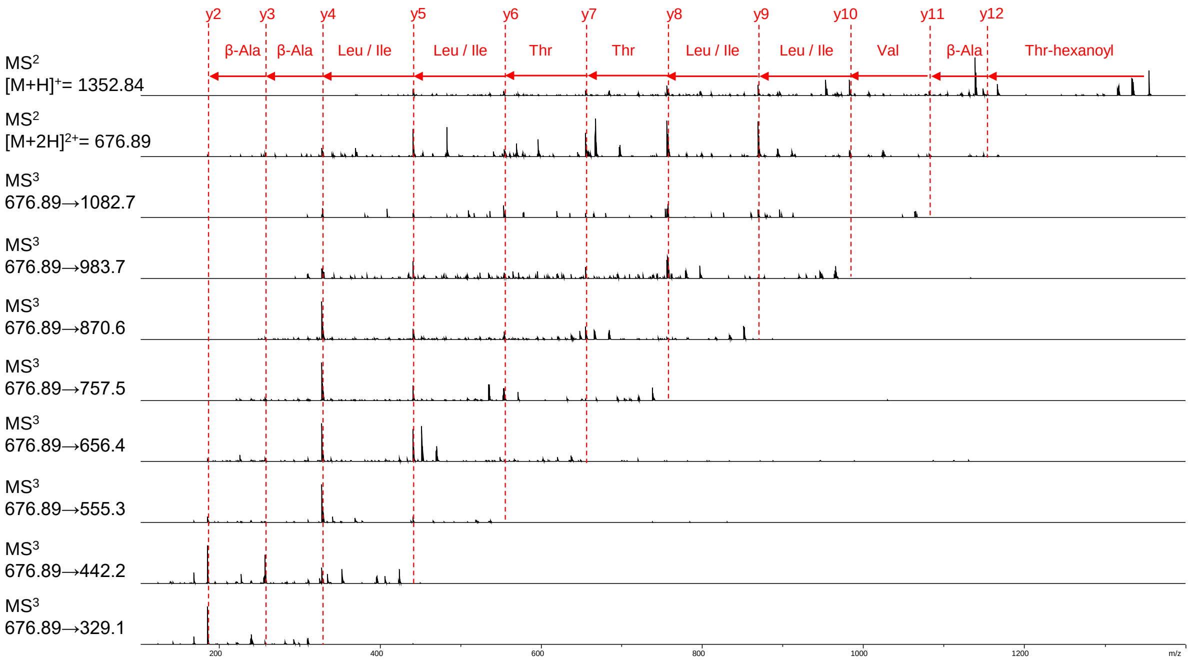

Supplementary Figure 22. MS^2 and MS^3 spectra of linearized XAM-1334. The complete serial of y-ions could be assigned in MS^3 spectra from the double charged xenoamicin ion (m/z = 676.9 [M+2H]$^{2+}$).

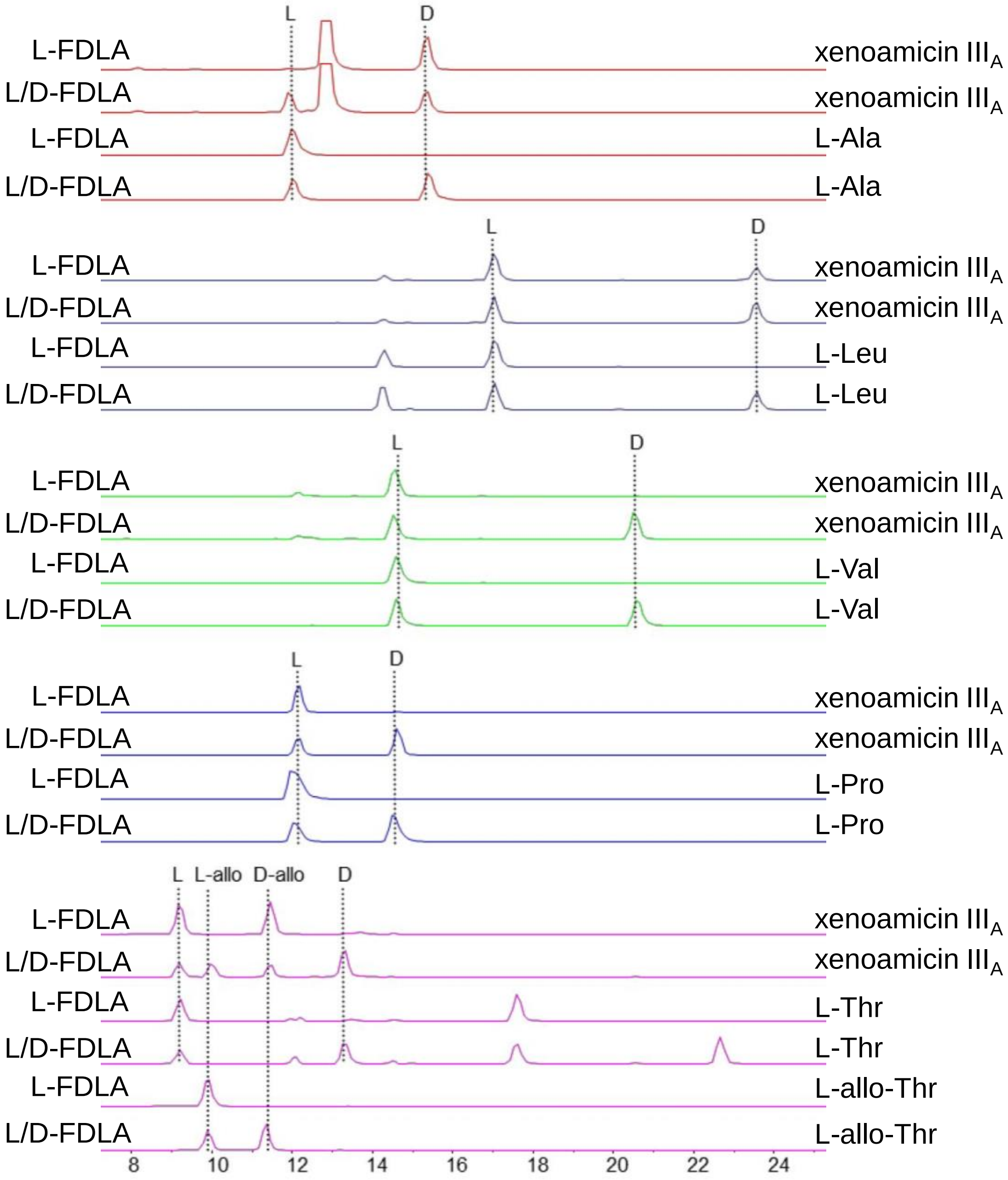

Supplementary Figure 23. Determination of the absolute configuration of amino acids in XAM-1320 by the advanced Marfey's method. The single amino acids were measured in the positive mode. The following m/z ratios ($[M+H]^+$) were used to detect the amino acids: alanine 384, leucine 426, valine 412, proline 410, threonine 414. For every amino acid the references are also shown.

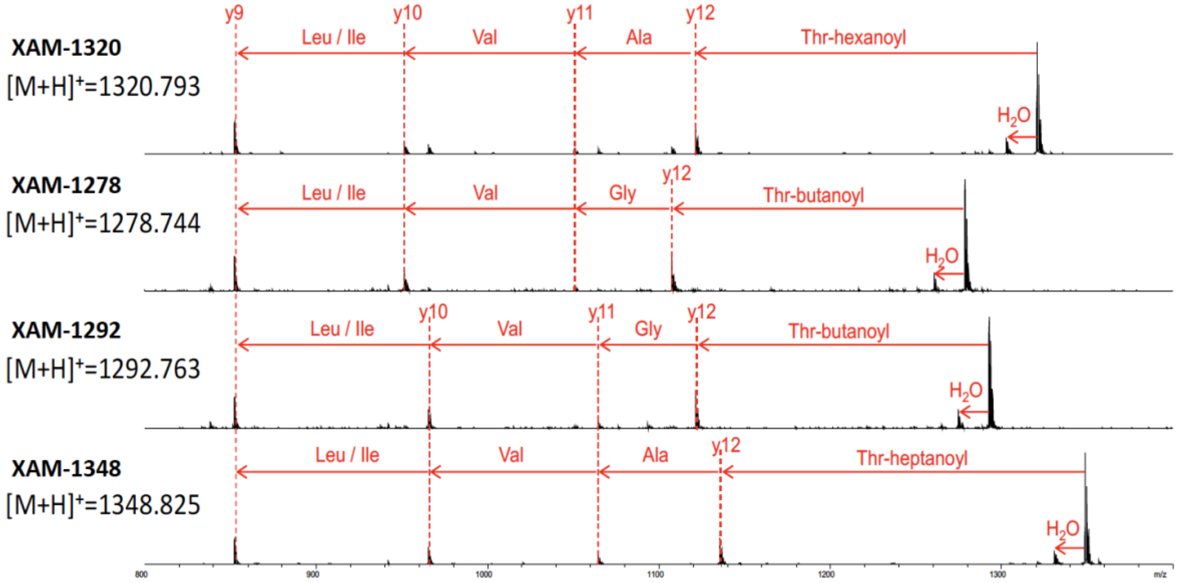

Supplementary Figure 24. MS^2 spectra of derivatives according to Xenoamicin-like Family. Compounds **14** (m/z = 1278.744 [M+H$^+$]), **15** (m/z = 1292.763 [M+H$^+$]) and **16** (m/z = 1348.825 [M+H$^+$]) differ to multiple of 14 Da from compound **12**. Mass differences could be localised between y12 and y10 ions.

25a

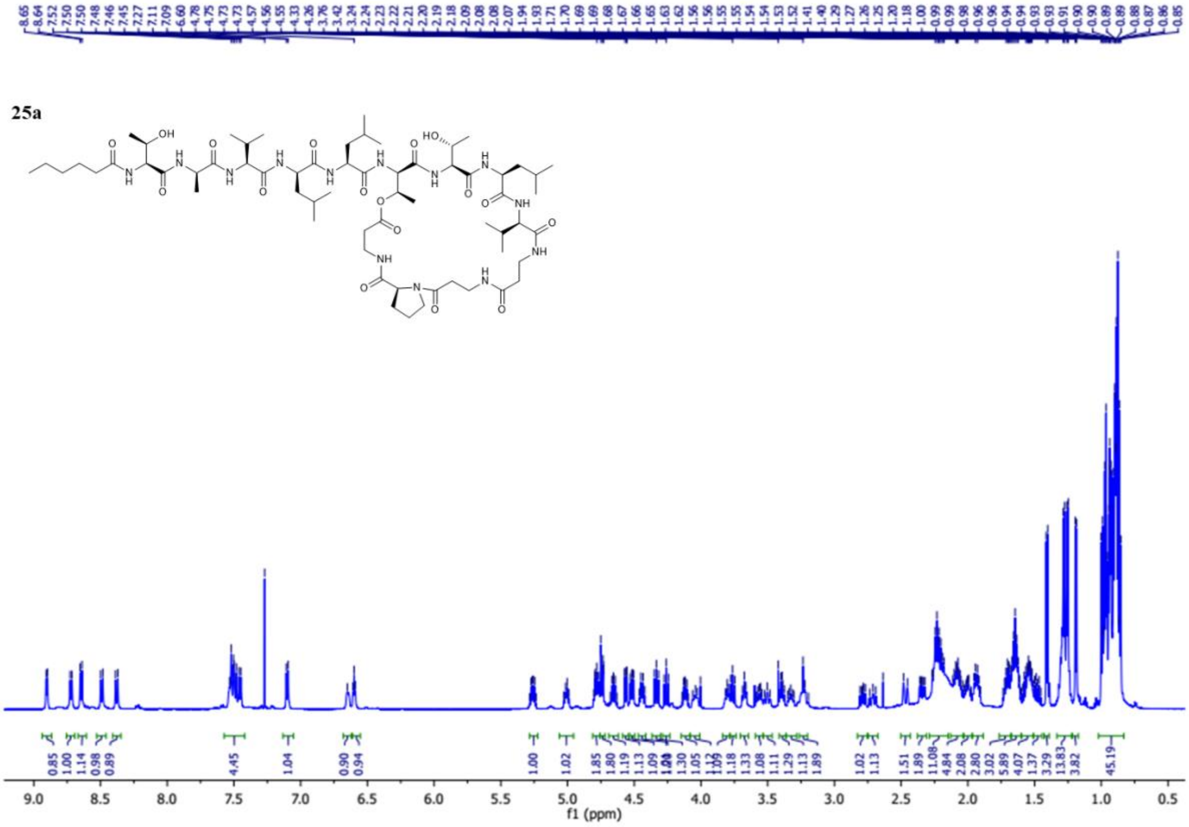

25b

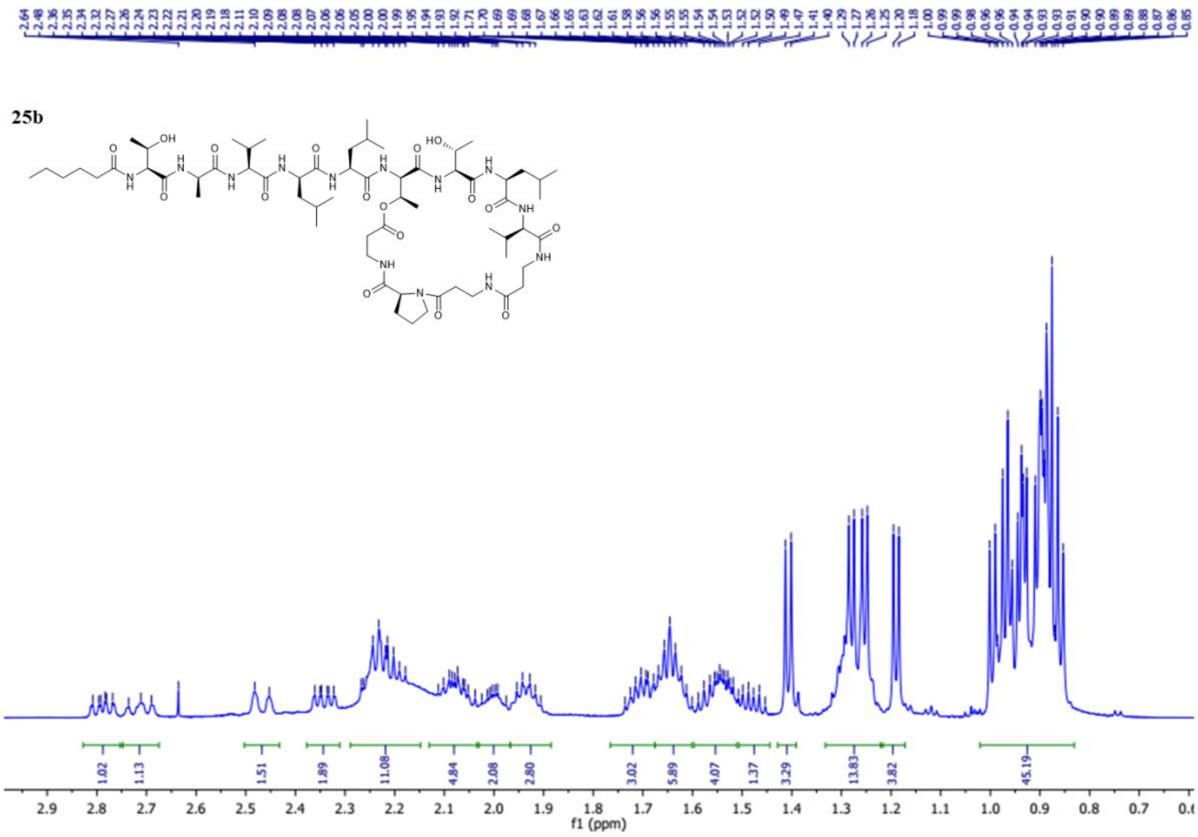

25c

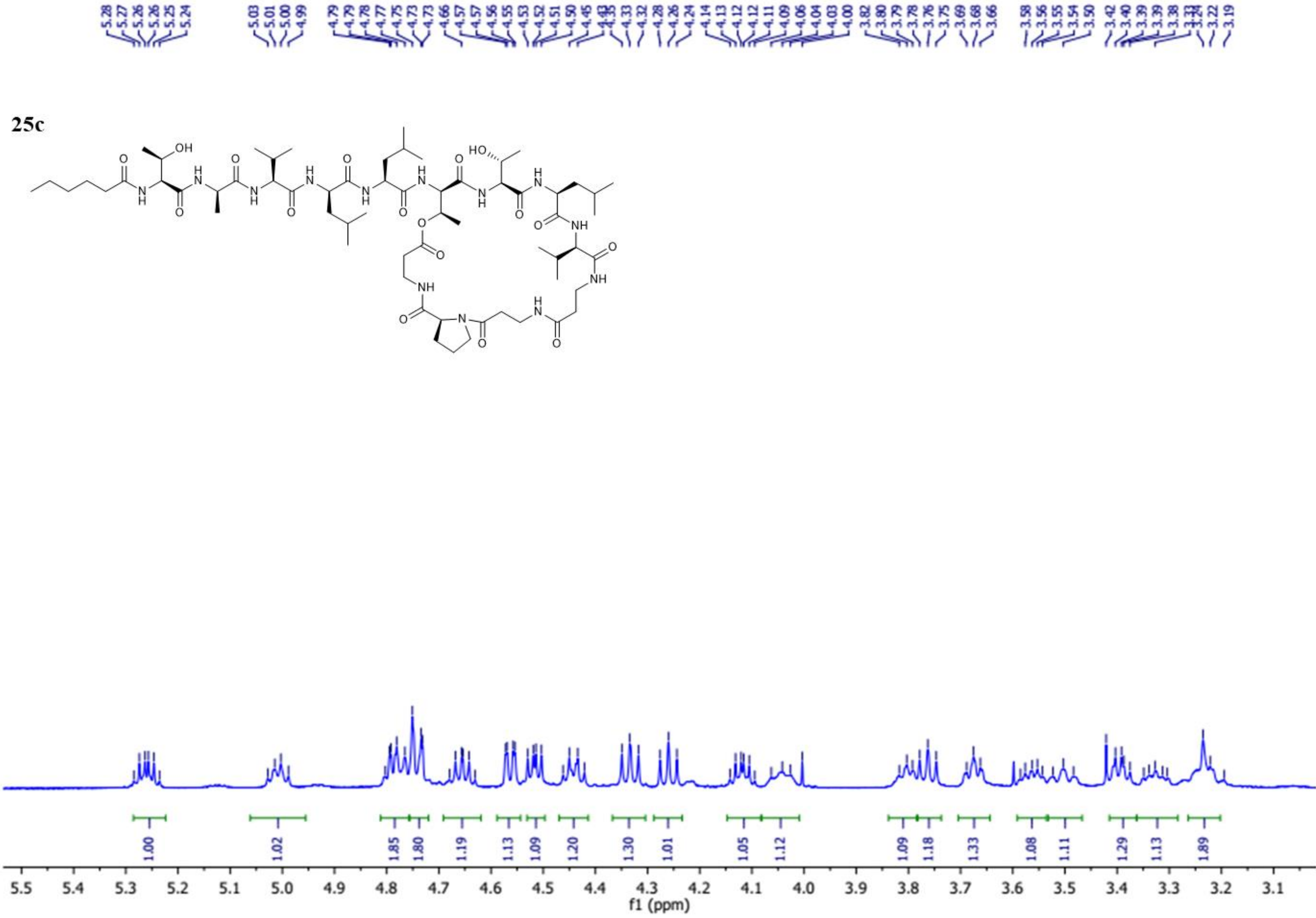

25d

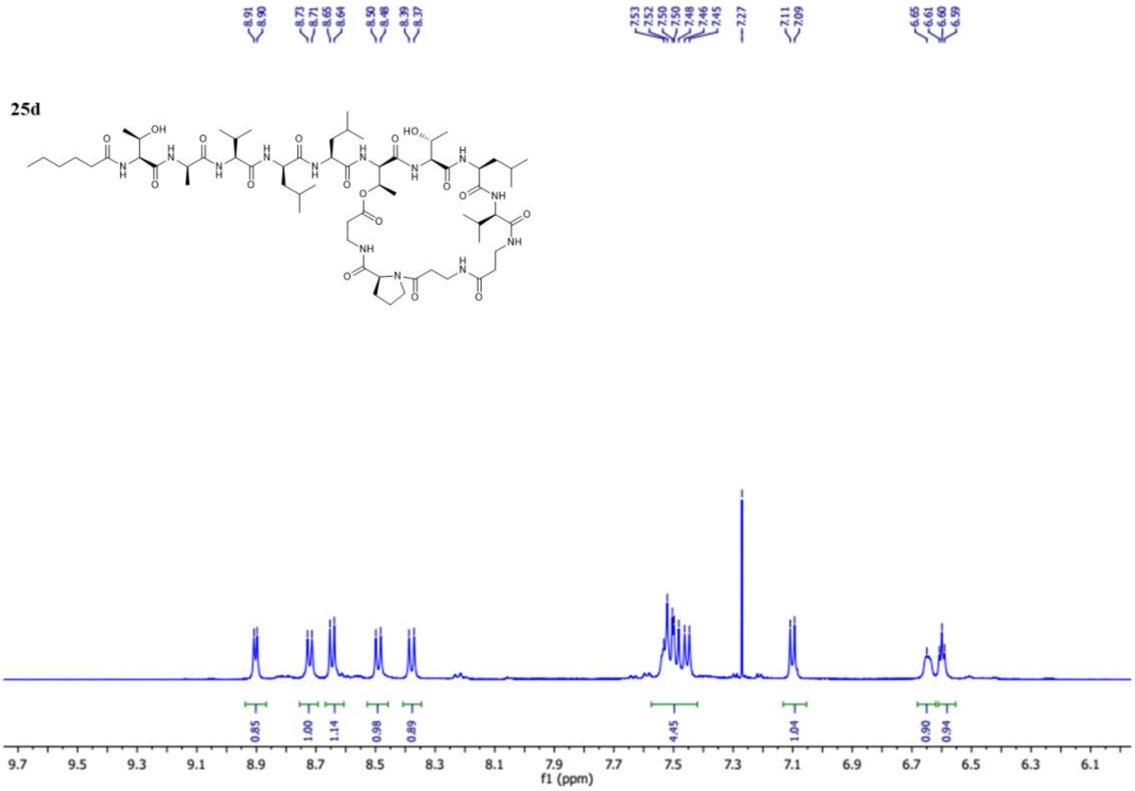

Supplementary Figure 25 (a-d). ^{1}H NMR (600 MHz) spectrum of compound **XAM-1320** in $CDCl_3$.

26a

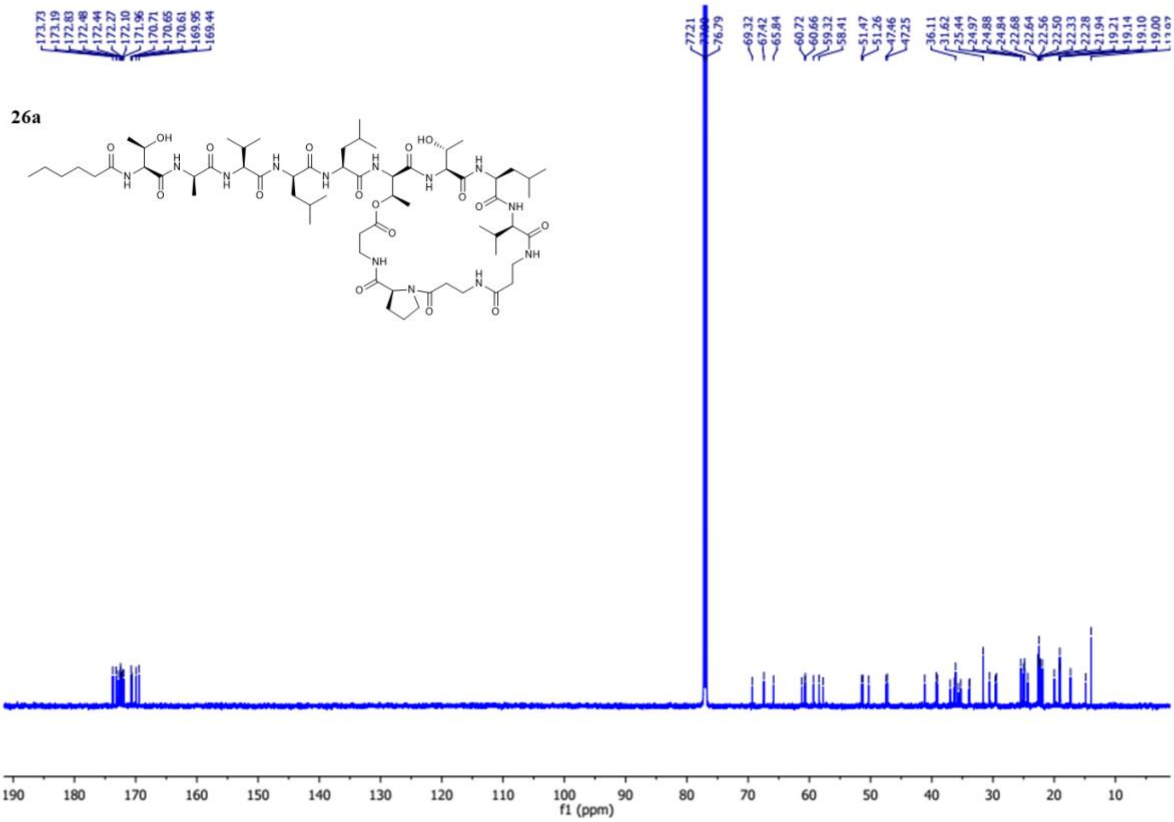

26b

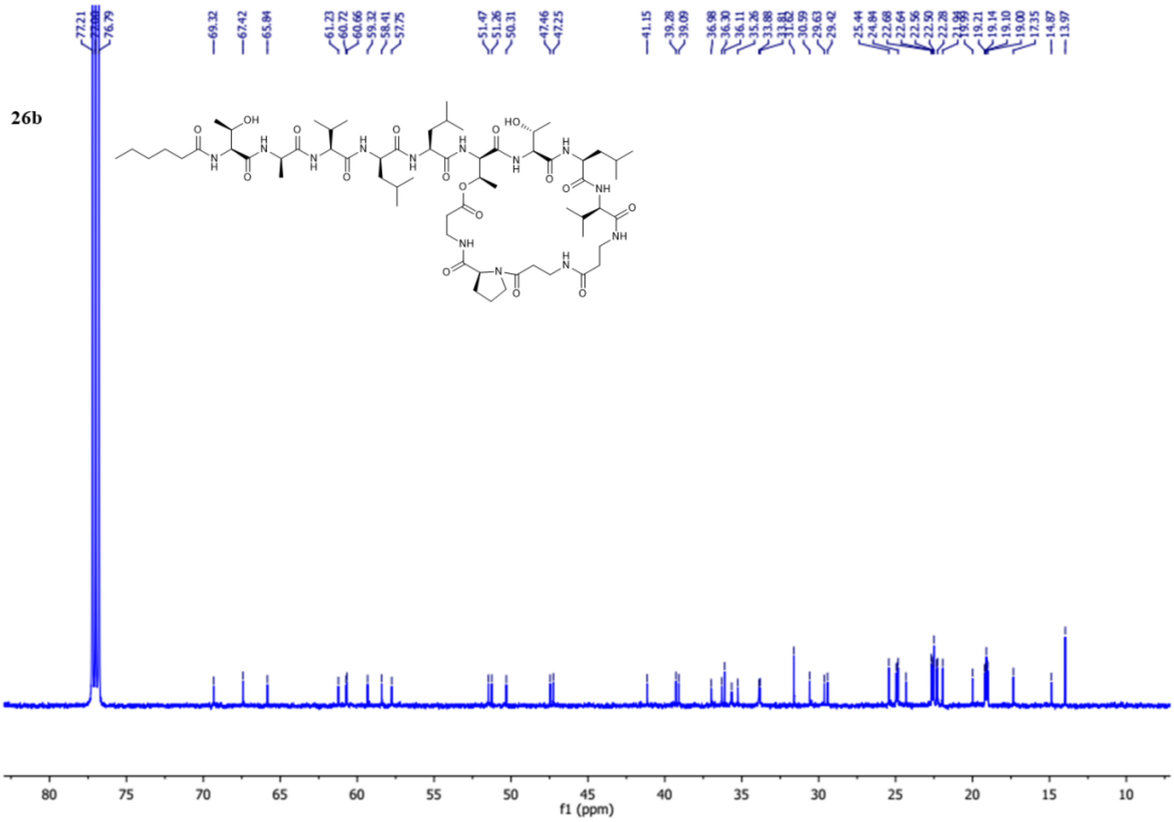

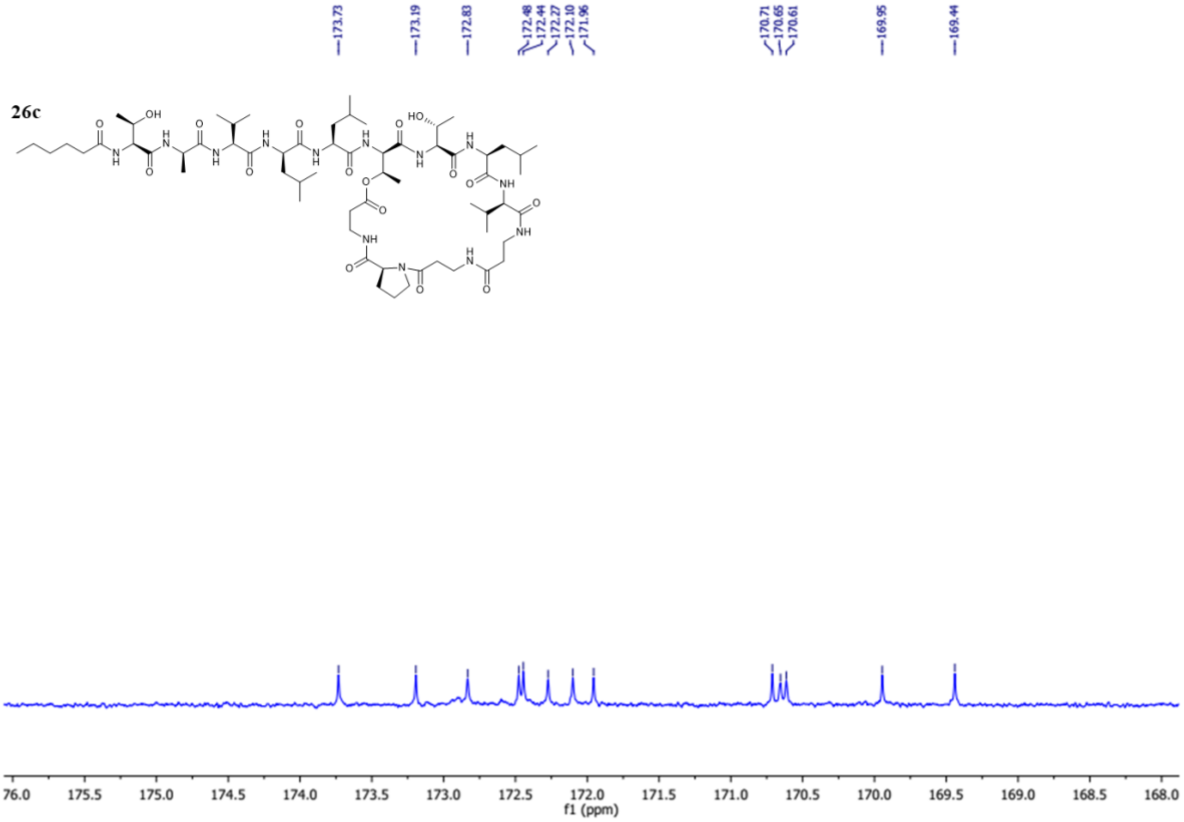

Supplementary Figure 26 (a-c). ^{13}C NMR (150 MHz) spectrum of compound **XAM-1320** in $CDCl_3$.

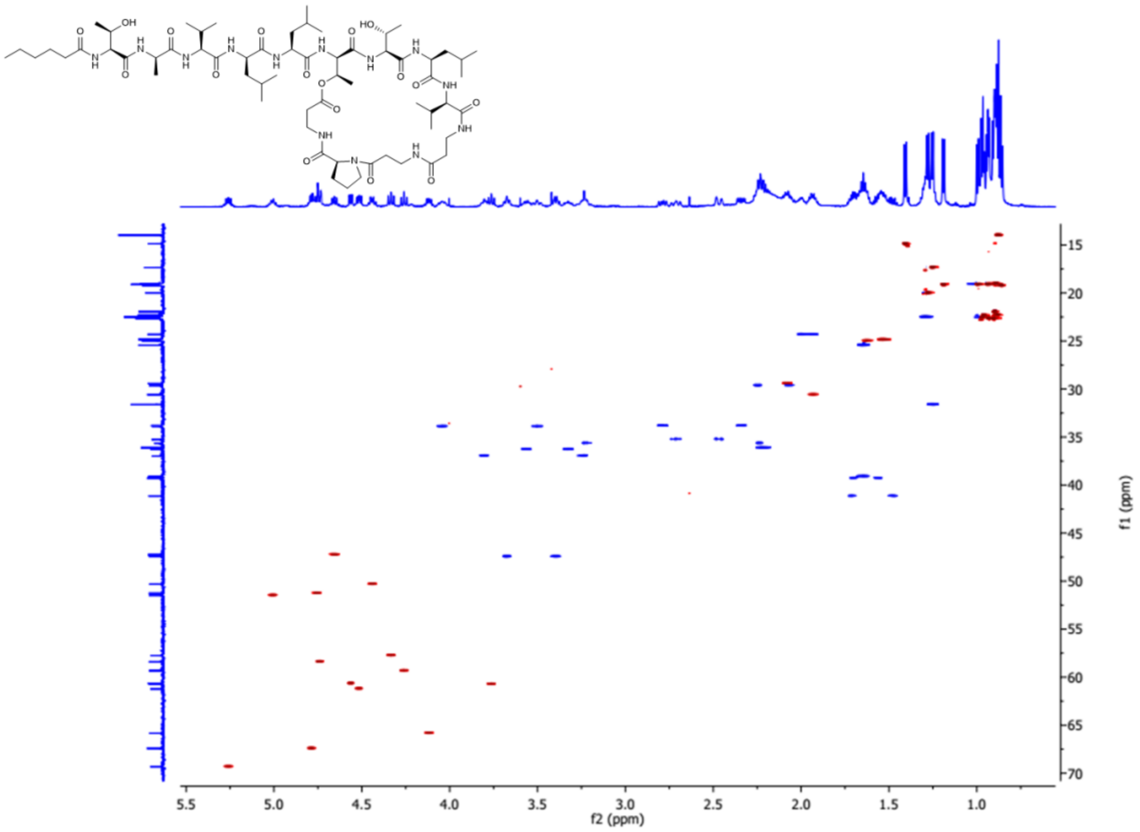

Supplementary Figure 27. HSQC (600 MHz) spectrum of compound **XAM-1320** in $CDCl_3$.

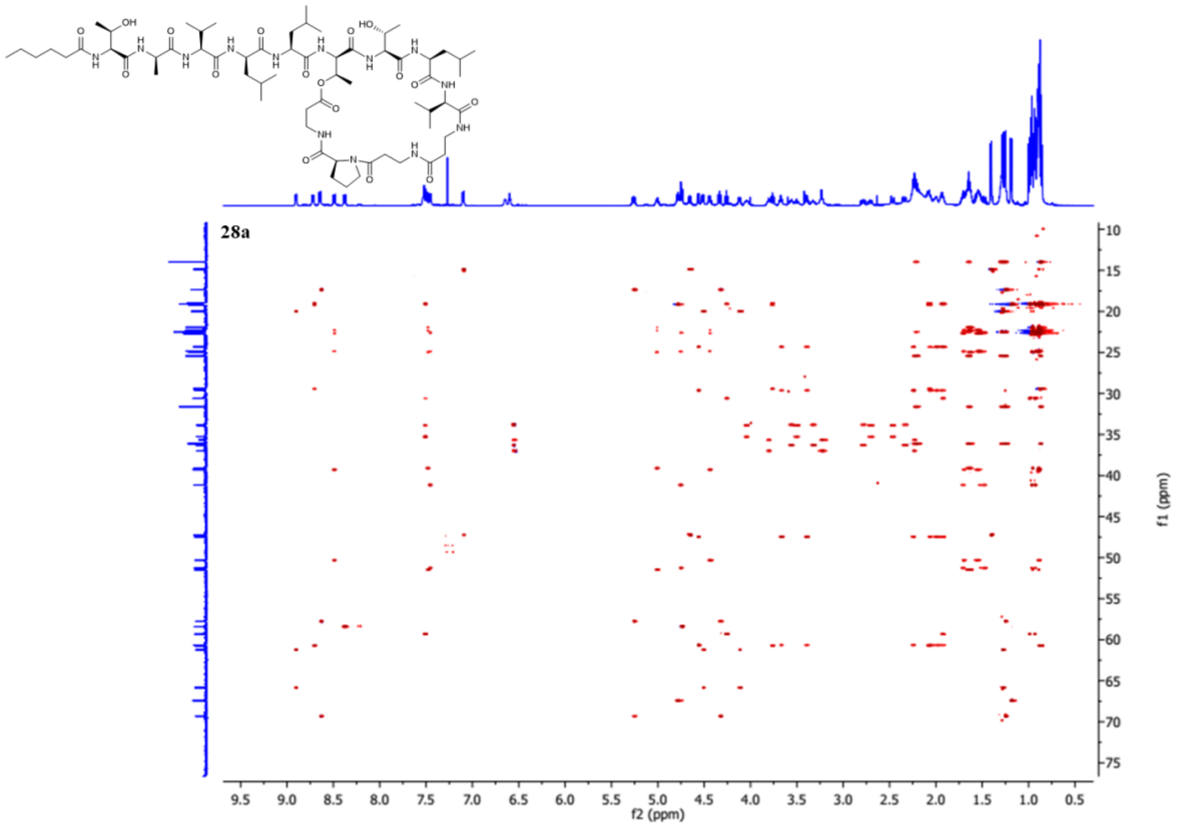

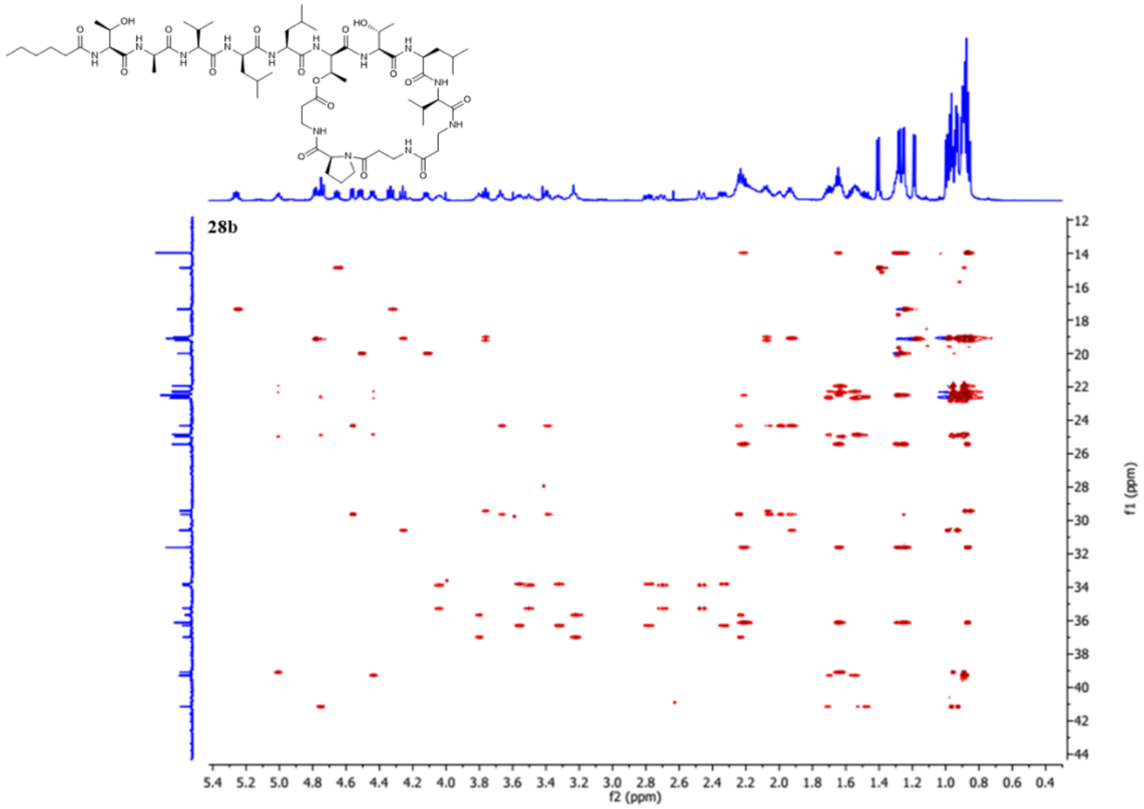

Supplementary Figure 28 (a-b). HSQC-TOCSY (600 MHz) spectrum of compound **XAM-1320** in $CDCl_3$.

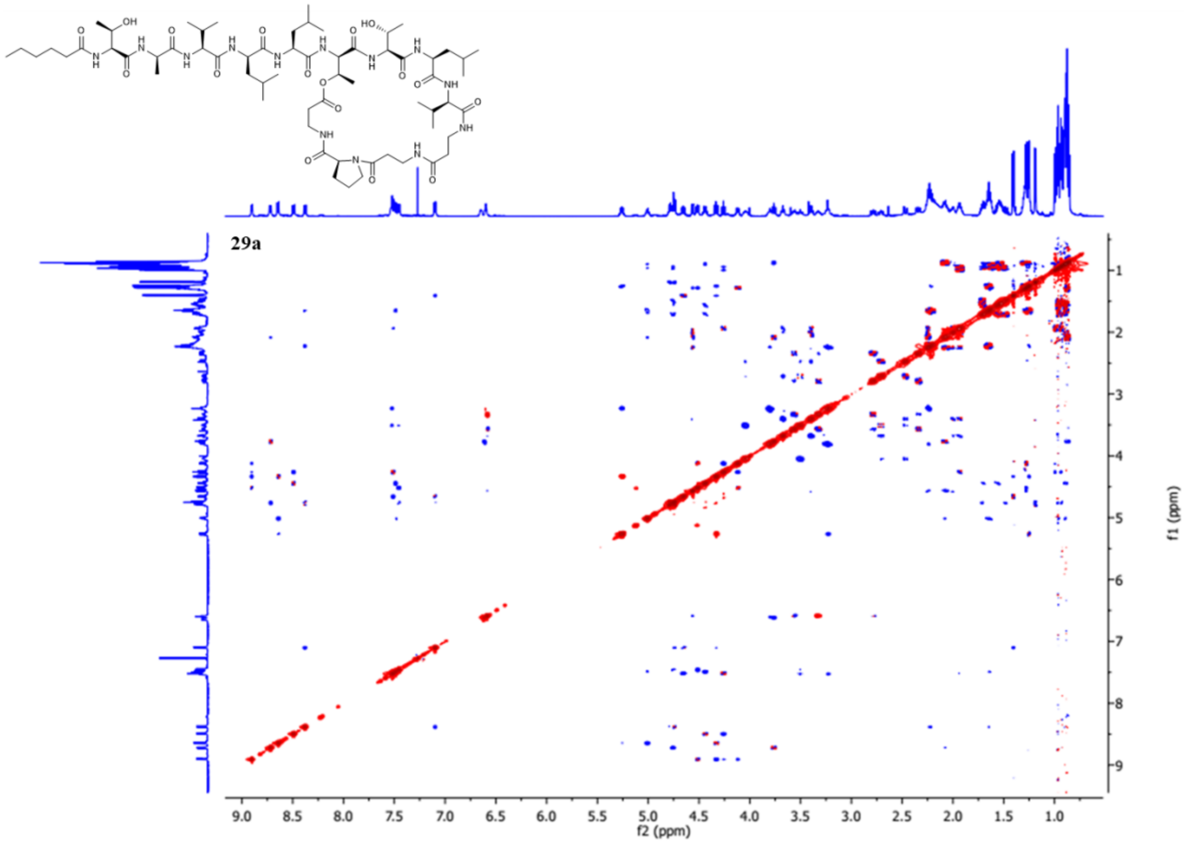

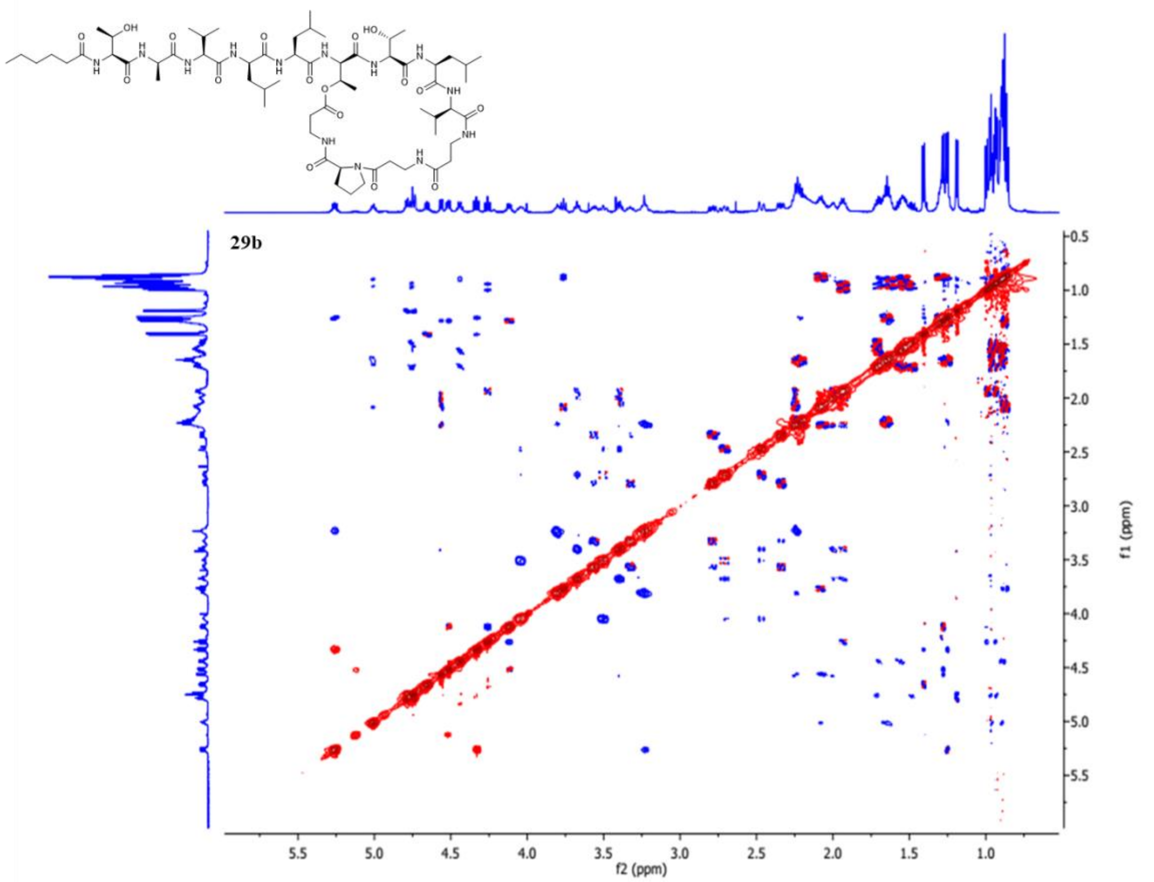

Supplementary Figure 29 (a-b). ROESY (600 MHz) spectrum of compound **XAM-1320** in $CDCl_3$.

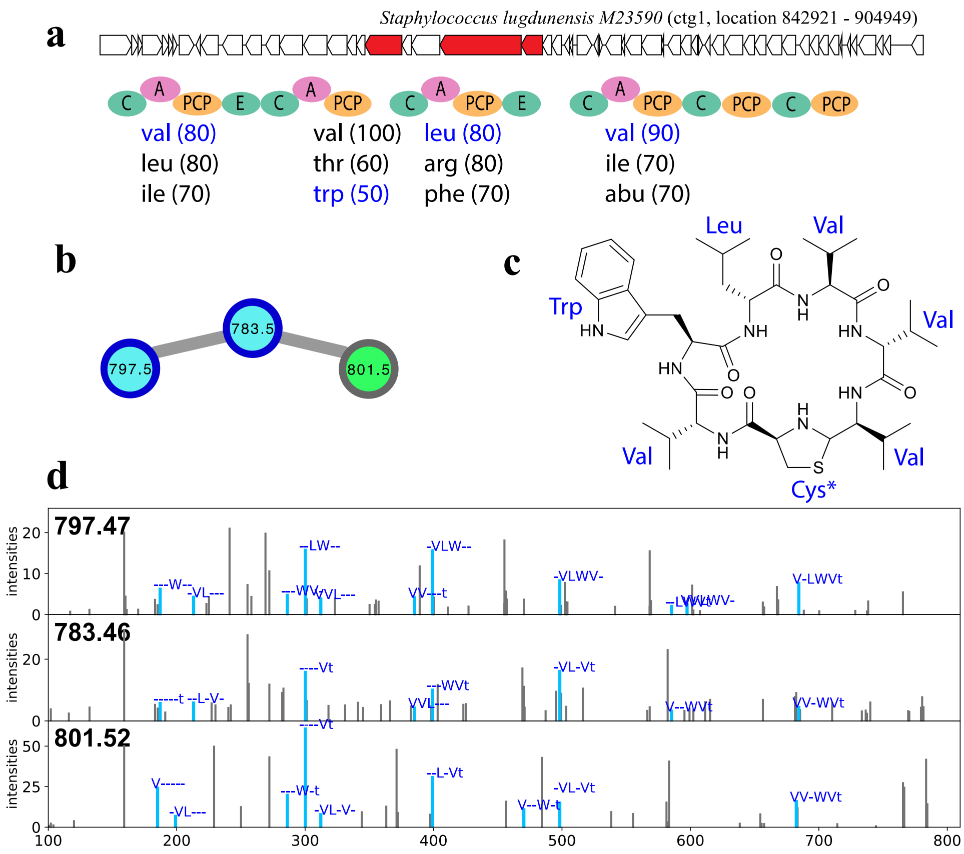

Supplementary Figure 30. Lugdunin NRP family matched by NRPminer in the SkinStaph dataset. (**a**) The BGC generating the core NRP in *S. lugdunensin* along with NRPS genes (shown in red) and the A-, C-, PCP-, and E-domains appearing in the corresponding NRPS. The rest of the genes in the corresponding contigs are shown in white. Three highest-scoring amino acids for each A-domain in this BGC (according to NRPSpredictor2[3] predictions) are shown below the corresponding A-domains. Amino acids appearing in the NRP VYLVV identified by NRPminer (with the lowest p-value) are shown in blue. The "Cys*" represent Cys-derived thiazolidine in the lugdunin structure. (**b**) Spectral network formed by spectra that originate from the NRPs in the lugdunin family. The known lugdunin NRPs are shown in blue, while the green node represents the novel variant identified by NRPminer. (**c**) Structure of a known lugdunin synthesized by a non-canonical assembly line. (**d**) For each matched NRP, an annotated spectrum of a PSM yielding the lowest p-values ($2.7x10^{-21}$, $3.6x10^{-15}$, and $7.5x10^{-15}$ from top to bottom) are shown.

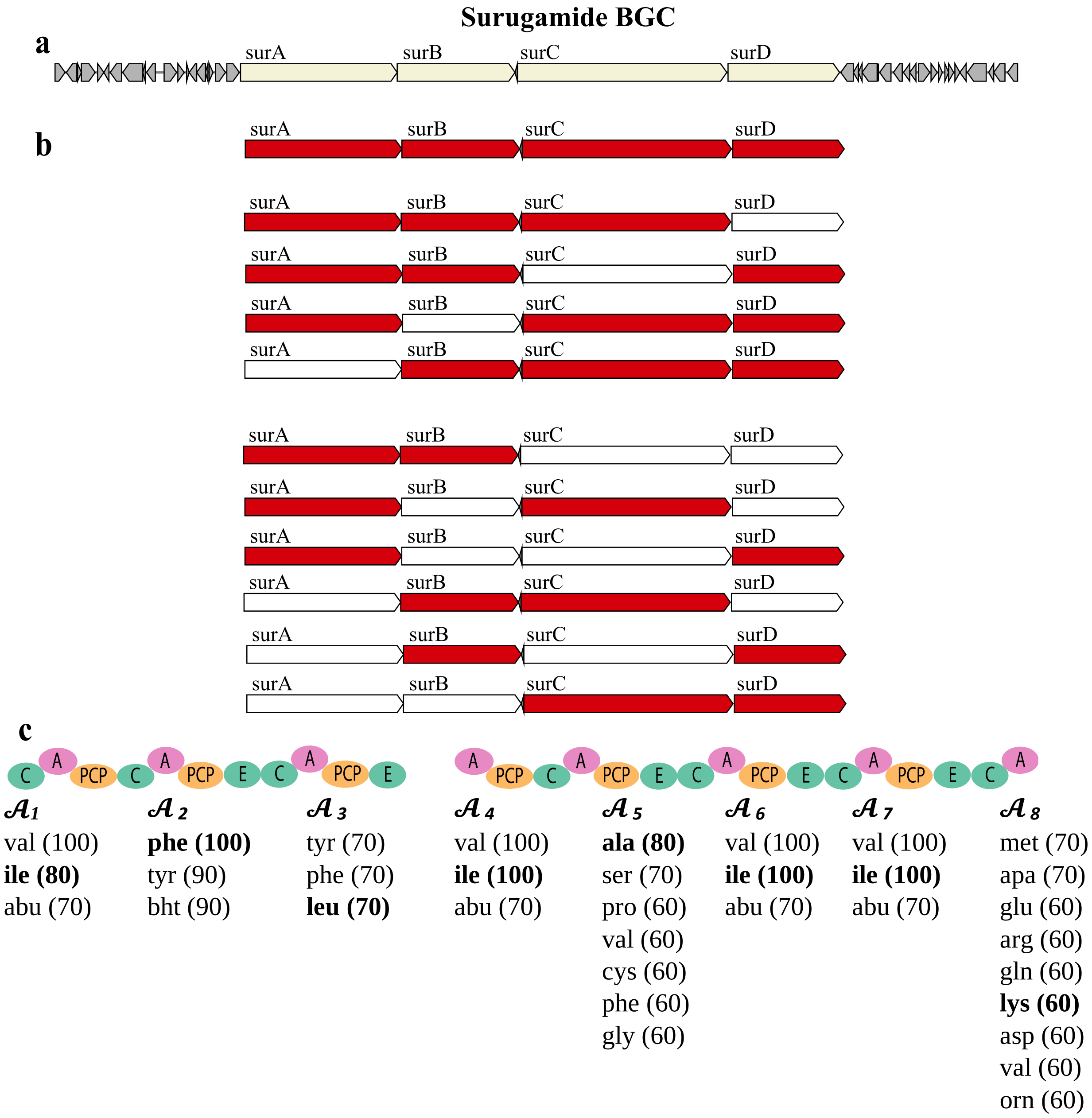

Supplementary Figure 31. Surugamide BGC and the surugamide assembly line formed by the *SurA* and *SurD* genes. (a) Surugamide BGC with four ORFs shown in yellow. **(b)** 11 assembly lines formed by deletion of zero, one and two ORFs (shown in red). NRPminer in the *OrfDel* mode explores all assembly lines generated by removing up to two ORFs. **(c)** The NRPS assembly line that synthesizes cyclic surugamides (formed by the SurA and SurD genes). At least three highest-scoring amino acids (along with their NRPSpredictor2[3] scores) are shown below each A-domain in this assembly line. Amino acids appearing in surugamide A are shown in bold. NRPminer considers all amino acids with the same score as the score of the third highest-scoring amino acid as illustrated in the case of the fifth and the eighth A-domains.

Supplementary Tables

Supplementary Table 1. The number of predicted core NRPs before and after filtering for 27 genomes in the XPF dataset. The column "#NRP producing BGCs" show the number of NRP-producing BGCs. Columns under "#unique core NRPs" show the number of core NRPs generated by NRPminer before and after filtering for each genome. For example, in the case of the *X. szentirmaii* DSM genome with 8 NRP-producing BGCs, NRPminer considers 253,027,076,774 core NRPs before filtering, while after filtering only 57,888 cores are retained. The five species corresponding to the datasets yielding the novel NRP families are shown in blue.

**Strain**	**#NRP producing BGCs**	**#unique core NRPs**	
		**before filtering**	**after filtering**
Xenorhabdus bovienii SS-2004	8	8,973,905	7,701
Xenorhabdus nematophila ATCC	6	18,043,657,358	18,062
Xenorhabdus doucetiae FRM16	8	3,726,625,228	8,013
Xenorhabdus poinarii G6	6	14,280	658
Photorhabdus luminescens PB45.5	10	2,994,745,388,283	8,333
Photorhabdus asymbiotica PB68.1	8	157,964	2,602
Xenorhabdus sp. DL20	9	94,818	2,187
Xenorhabdus sp. 30TX1	8	76,044,111	7,287
Xenorhabdus vietnamensis	15	3,373,109,836	21,648
Xenorhabdus beddingii DSM 4764	8	13,721,302	2,998
Photorhabdus temperata	9	42,555,972,979,030	6,924
Photorhabdus asymbiotica PB68.1	8	160,034	5,136
Xenorhabdus budapestensis 16342	7	149,918,342	51,600
Xenorhabdus ehlersii DSM 16337	10	5,026,725	7,542
Xenorhabdus innexi DSM 16336	10	4,957,948,632	9,184
Xenorhabdus szentirmaii US	8	360,039,991,874	57,888
Xenorhabdus mauleonii	10	51,502,147,078	19,400
Xenorhabdus miraniensis	14	11,679,221,261	14,658
Xenorhabdus szentirmaii	8	253,027,076,774	57,888
Xenorhabdus sp. KK7.4	9	5,036,899,357	17,300
Xenorhabdus hominickii DSM	13	60,224,436	6,688
Xenorhabdus stockiae DSM 17904	10	1,159,012,484,964	7,896
Xenorhabdus ishibashii	7	19,911,786	2,547
Xenorhabdus sp. KJ12.1	10	11,916,878,760	10,458
Xenorhabdus kozodoi DSM 17907	11	87,750	2,192
Xenorhabdus cabanillasii JM26	9	80,529,848	47,856
Photorhabdus temperata	11	567,909,518,582	4,823

Supplementary Table 2. PSMs identified by NRPminer in the XPF dataset representing the known NRP families. For each NRP family, the information about the PSM with the lowest p-value among all PSMs corresponding to the spectra representing the known NRPS in that family is listed. The column “matched genome” shows the name of the organism whose BGCs generated the putative NRP structure corresponding to that PSM and the column "BGC position” shows the contig and the starting and ending nucleotide position of the BGC in that contig. Columns “precursor mass” and “charge” show the precursor mass and the charge state of matched spectrum. The p-values are computed based on MCMC approach using MS-DPR[4] with 10000 simulations.

**NRP family name**	**matched genome**	**BGC position**	**p-value**	**precursor mass**	**charge**
GameXPeptide	*Photorhabdus asymbiotica* PB68.1	ctg1: 3584973 - 3640476	1.5×10^{-25}	586.394	1
PAX peptide	*Xenorhabdus nematophila* ATCC 19061	ctg1: 11609 - 67919	9.9×10^{-18}	826.538	1
Xenobactin	*Xenorhabdus mauleonii* DSM 17908	ctg11: 65321 - 162527	5.0×10^{-21}	756.425	1
Szentiamide	*Xenorhabdus szentirmaii* DSM 16338	ctg1: 762001 - 821352	7.0×10^{-31}	838.404	1
Taxlllaid	*Xenorhabdus bovienii* SS-2004	ctg1: 739318 - 804275	1.2×10^{-30}	808.55	1
Xentrivalpeptide	*Xenorhabdus* sp. KK7.4	ctg14: 6760-112451	6.4×10^{-37}	430.749	2
Ambactin	*Xenorhabdus miraniensis* DSM 17902	ctg6: 132143-191993	5.4×10^{-16}	751.41	1
Xenoamicin	*Xenorhabdus vietnamensis* DSM 22392	ctg9: 1-75156	3.3×10^{-56}	1300.8	1
Rhabdopeptide	*Xenorhabdus stockiae* DSM 17904	ctg14: 1-77935	6.1×10^{-17}	599.427	1

Supplementary Table 3. Sum formula of protegomycin (PRT) derivatives. Sum formula of the PRT variants identified via HPLC-MS analysis in extracts from *X. doucetiae*, *X. poinarii* and 30TX1.

Strain	Protegomycin (PRT)	*m/z*	sum formula	Δppm
X. doucetiae	PRT-1037	1037.4679	$C_{57}H_{64}N_8O_{11}$	8.5
	PRT-1051	1051.4830	$C_{58}H_{66}N_8O_{11}$	8.9
	PRT-1065	1065.4953	$C_{59}H_{68}N_8O_{11}$	11.8
	PRT-1012	1012.4723	$C_{56}H_{65}N_7O_{11}$	9.0
	PRT-1021	1021.4723	$C_{57}H_{64}N_8O_{10}$	9.3
	PRT-1046	1046.4551	$C_{59}H_{63}N_7O_{11}$	10.2
	PRT-1085	1085.4665	$C_{61}H_{64}N_8O_{11}$	9.4
X. poinarii	PRT-945	945.4294	$C_{54}H_{56}N_8O_8$	4.1
	PRT-929	929.4345	$C_{54}H_{56}N_8O_7$	2.8
	PRT-922	922.4106	$C_{52}H_{55}N_7O_9$	3.1
	PRT-911	911.4439	$C_{51}H_{58}N_8O_8$	1.3
30TX1	PRT-1108	1108.4841	$C_{63}H_{65}N_9O_{10}$	7.8
	PRT-1092	1092.4920	$C_{63}H_{65}N_9O_9$	5.3
	PRT-1076	1076.5029	$C_{63}H_{65}N_9O_8$	4.7

Supplementary Table 4. ^{1}H (500 MHz) and ^{13}C (125 MHz) NMR spectroscopic data for PRT-1037. ^{1}H (500 MHz) and ^{13}C (125 MHz) NMR spectroscopic data for PRT-1037 in DMSO-d_6 (δ in ppm and J in Hz).

no.	PRT-1037	
	δ_C, type	δ_H (mult., J)
1	14.25	0.88 (d, 7.4)
2	18.76	1.59 (dq, 14.6, 7.2)
3	37.45	2.21 (t, 7.2)
4	174.41	
5		8.27 (br s)
6	56.00	4.08 (m)
7	31.04	1.48 (m)
8	22.58	1.42 (br s)
9	27.98	1.42 (overlap)
10	38.17	3.31 (m)
		2.78 (m)
11		7.62 (br s)
12	171.73	
13	55.39	4.35 (m)
14	27.05	3.20 (m)
		3.03 (dd, 14.7, 9.0)
15	110.82	
16	123.95	7.22 (d, 1.9)
17		10.86 (br s)
18	136.59	
19	111.85	7.59 (d, 8.0)
20	118.82	7.00 (t, 7.2)
21	121.43	7.08 (t, 7.2)
22	118.74	7.35 (d, 8.0)
23	127.67	
24		8.11 (br s)
25	171.83	
26	55.83	4.22 (m)
27	37.12	2.60 (m)
		2.53 (overlap)
28	128.14	
29	130.32	6.81 (d, 8.5)
30	115.28	6.60 (d, 8.5)
31	156.27	
32	115.28	6.60 (d, 8.5)
33	130.32	6.81 (d, 8.5)
34		8.11 (d, 6.6)
35	171.68	
36	56.35	4.22 (overlap)
37	37.26	2.92 (overlap)
		2.80 (overlap)
38	128.69	
39	130.58	6.81 (overlap)
40	115.42	6.61 (overlap)
41	156.07	
42	115.42	6.61 (overlap)
43	130.58	6.81 (overlap)
44		8.11 (overlap)
45	171.92	
46	55.76	4.22 (overlap)
47	36.3	2.92 (overlap)

		2.80 (overlap)
48	128.67	
49	130.80	6.81 (overlap)
50	115.21	6.61 (overlap)
51	156.20	
52	115.21	6.61 (overlap)
53	130.80	6.81 (overlap)
54		8.05 (d, 6.6)
55	171.06	
56	55.53	3.96 (m)
57	34.70	2.92 (overlap)
		2.80 (overlap)
58	128.48	
59	130.61	6.81 (overlap)
60	115.32	6.61 (overlap)
61	156.29	
62	115.32	6.61 (overlap)
63	130.61	6.81 (overlap)
64		8.05 (overlap)
65	173.61	

Supplementary Table 5. NMR spectroscopic data (600 MHz (^{1}H), 125 MHz (^{13}C) in $CDCl_3$) of XAM-1320. NMR spectroscopic data (600 MHz (^{1}H), 125 MHz (^{13}C) in $CDCl_3$) of **XAM-1320**; δ in ppm; HM, hexanoyl moiety.

Spin Sys.	Pos.	δ_C	δ_H
1-HM	C=O	173.73	
	α	36.12	2.23
	β	31.64	1.28
	γ	25.46	1.66
	δ	22.50	1.29
	ε	14.00	0.88
2-Thr	C=O	172.09	
	NH		8.38
	α	58.41	4.74
	β	67.43	4.78
	γ	19.14	1.18
3-Ala	C=O	172.48	
	NH		7.10
	α	47.25	4.66
	α	14.87	1.41
4-Val	C=O	172.44	
	NH		7.51
	α	59.32	4.26
	β	30.60	1.94
	γ1	19.10	1.00
	γ2	19.10	0.94
5-Leu	C=O	169.95	
	NH		8.50
	α	50.31	4.44
	β	39.28	1.70
	β		1.56
	γ	24.84	1.53
	δ1	22.68	0.89
	δ2	22.28	0.88
6-Leu	C=O	173.20	
	NH		7.48
	α	51.48	5.01
	β	39.09	1.65
	γ	24.97	1.64
	δ1	22.34	0.97
	δ2	21.95	0.90
7-Thr	C=O	169.44	
	NH		8.64
	α	69.34	5.26
	β	57.76	4.33
	γ	17.37	1.25
8-Thr	C=O	170.65	
	NH		8.91
	α	65.84	4.12
	β	61.22	4.52
	γ	20.00	1.28
9-Leu	C=O	170.61	
	NH		7.46
	α	51.28	4.76
	β	41.18	1.72
	β		1.49
	γ	24.88	1.53
	δ1	22.64	0.93
	δ2	22.56	0.98
10-Val	C=O	171.95	
	NH		8.73
	α	60.71	3.76
	β	29.43	2.07
	γ1	19.21	0.86
	γ2	19.00	0.89
11-β-Ala	C=O	172.09	
	NH		6.49
	α	37.00	3.81
	α		3.24
	β	35.66	3.24
	β		2.25
12-β-Ala	C=O	172.27	
	NH		7.53
	α	33.89	4.06
	α		3.52
	β	35.26	2.71
	β		2.47
13-Pro	C=O	172.83	
	α	60.67	4.57
	β	29.64	2.25
	β		2.07
	γ	24.33	2.00
	γ		1.93
	δ	47.46	3.68
	δ		3.40
14-β-Ala	C=O	170.71	
	NH		6.56
	α	36.31	3.58
	α		3.35
	β	33.81	2.78
	β		2.34

Supplementary Table 6. ROE list for XAM-1320 NRP. ROE list with upper and lower distance restraint limits (90%, 110%) including pseudoatom correction from experimentally determined distance for 3D modelling of XAM-1320. Average distance and average violation of single distance restraints over ten conformations from the final MD trajectory (after energy minimization) are shown.

ROEs

ATOM1	ATOM2	LOWER	UPPER	AV_DIST	AV_VIOL
8-THR NH	8-THR γ	4	5.8	4.45	0
8-THR NH	3-ALA γ	4	5.8	4.01	0
8-THR NH	8-THR β	2.5	3.1	2.92	0
4-VAL α	8-THR NH	2.8	3.4	2.86	0
7-THR α	8-THR NH	2.2	2.7	2.42	0
8-THR α	8-THR NH	2.9	3.5	2.71	0.19
7-THR β	8-THR NH	3.5	4.3	3.82	0
10-VAL NH	10-VAL β	2.6	3.2	2.41	0.19
10-VAL α	10-VAL NH	3	3.7	2.88	0.12
9-LEU α	10-VAL NH	2.2	2.6	2.44	0
6-LEU α	10-VAL NH	3.1	3.8	4.33	0.53
7-THR NH	7-THR γ	3.6	5.5	4.2	0
7-THR α	7-THR NH	2.87	3.5	2.8	0.07
6-LEU α	7-THR NH	2	2.5	2.52	0.02
7-THR NH	7-THR β	2.8	3.4	2.85	0
5-LEU NH	8-THR α	3.6	4.4	4.83	0.43
4-VAL α	5-LEU NH	2.1	2.6	2.35	0
5-LEU NH	7-THR α	3.3	4.1	4.15	0.05
5-LEU α	5-LEU NH	2.8	3.4	2.94	0
2-THR NH	2-THR γ	2.5	4	3.63	0
2-THR NH	Acyl α	2.4	3.9	2.87	0
2-THR NH	Acyl β	2.6	4	3.43	0
2-THR NH	3-ALA NH	2.3	2.8	2.81	0.01
2-THR NH	4-VAL NH	3.3	4.1	4.27	0.17
7-THR α	12-ALA NH	3.6	4.4	5.07	0.68
8-THR α	12-ALA NH	3.2	3.9	4.21	0.31
5-LEU NH	8-THR NH	4.2	5.2	4.36	0
10-VAL NH	10-VAL γ	3.1	4.8	3.63	0
7-THR β	12-ALA NH	3.7	4.5	3.38	0.32
4-VAL NH	3-ALA γ	4.3	6.2	4.5	0
4-VAL NH	4-VAL β	2.7	3.3	3.1	0
4-VAL α	4-VAL NH	2.7	3.4	2.84	0
4-VAL NH	3-ALA α	2.1	2.6	2.7	0.1
2-THR α	4-VAL NH	3.6	4.5	4.26	0
2-THR β	4-VAL NH	3.3	4.1	2.89	0.41
4-VAL NH	3-ALA NH	3.4	4.2	2.97	0.43
6-LEU NH	6-LEU β	2.4	3.9	2.45	0
5-LEU α	6-LEU NH	2.1	2.6	2.26	0
6-LEU α	6-LEU NH	2.7	3.4	3.01	0
9-LEU NH	8-THR γ	3.7	5.5	4.35	0
2-THR β	9-LEU NH	3.8	4.6	4.74	0.14
8-THR α	9-LEU NH	2	2.5	2.34	0
9-LEU α	9-LEU NH	2.7	3.3	3.06	0
3-ALA NH	7-THR γ	3.5	5.3	3.23	0.27
3-ALA NH	Acyl α	3.4	5.1	3.64	0
3-ALA NH	7-THR α	3	3.7	2.76	0.24
3-ALA α	3-ALA NH	2.7	3.4	3.01	0
2-THR α	3-ALA NH	3	3.6	3.7	0.1
2-THR β	3-ALA NH	3.5	4.2	4.22	0.01
7-THR β	7-THR γ	2.4	2.9	2.49	0

7-THR β	3-ALA γ	4	5.9	5.75	0
7-THR β	Acyl α	3.1	4.7	5.09	0.39
6-LEU α	10-VAL β	2.5	3	3.42	0.42
9-LEU α	9-LEU δ	2.6	4.2	4.67	0.47
9-LEU α	9-LEU δ	2.6	4.2	3.17	0
2-THR β	2-THR γ	2.2	3.7	2.47	0
2-THR α	2-THR γ	2.3	3.8	2.88	0
3-ALA α	3-ALA γ	2.1	3.6	2.66	0
13-PRO α	8-THR γ	2.6	4.2	3.58	0
13-PRO α	13-PRO δ	2.6	3.2	3.48	0.28
13-PRO α	13-PRO δ	3.6	4.5	4.15	0
13-PRO α	13-PRO β	2.6	3.2	2.24	0.36
8-THR α	8-THR γ	2.3	3.9	2.82	0
5-LEU α	5-LEU δ	2.4	3.9	3.59	0
5-LEU α	5-LEU γ	2.6	3.15	2.51	0.09
7-THR α	7-THR γ	2.4	3.9	3.09	0
7-THR α	3-ALA γ	2.6	4.2	4.19	0
4-VAL β	7-THR α	3.6	4.5	5.01	0.51
4-VAL α	4-VAL β	2.7	3.3	2.38	0.32
4-VAL α	8-THR β	2.1	2.6	2.34	0
4-VAL α	4-VAL γ	2.5	3.1	3.7	0.6
4-VAL α	4-VAL γ	2.5	3.1	3.16	0.06
8-THR β	4-VAL γ	2.2	3.7	3.36	0
8-THR β	4-VAL γ	3.1	4.8	5.15	0.35
8-THR β	8-THR γ	3	4.6	2.71	0.29
10-VAL α	10-VAL γ	2.5	4.1	3.44	0
10-VAL α	10-VAL γ	2.5	4.1	3.04	0
10-VAL α	10-VAL β	2.8	3.4	3	0
12-ALA α1	12-ALA NH	2.4	3	2.27	0.13
12-ALA α1	12-ALA β1	2.5	3	2.58	0
12-ALA α2	12-ALA β2	2.4	3	2.57	0
12-ALA α2	12-ALA β1	3.3	4.1	3.16	0.14
12-ALA β1	13-PRO δ	2.6	3.2	3.46	0.26
12-ALA β1	13-PRO δ	2.2	2.7	2.48	0
12-ALA β2	13-PRO δ	2.3	2.8	2.56	0
12-ALA β2	13-PRO δ	2.8	3.5	2.61	0.19
14-ALA α1	14-ALA β1	2.4	3.1	2.6	0
14-ALA β1	14-ALA α2	2.4	3.1	3.15	0.05
14-ALA α1	14-ALA β2	2.4	3.1	2.68	0
14-ALA β1	14-ALA α2	2.4	3.1	2.61	0
12-ALA β1	14-ALA NH	3.3	4.1	3.53	0
12-ALA α1	12-ALA NH	3.4	4.2	3.12	0.28
12-ALA α2	12-ALA NH	2.4	3	2.37	0.03
12-ALA NH	12-ALA β1	3.5	4.4	3.36	0.14
12-ALA β1	12-ALA NH	2.3	2.8	2.44	0
9-LEU NH	12-ALA α2	2.6	3.3	2.88	0
8-THR NH	14-ALA α	3.8	5.5	3.58	0.22
8-THR NH	14-ALA β	3.9	5.7	4.89	0
7-Thr NH	14-ALA α	3.6	5.3	4.47	0
5-LEU NH	5-LEU β	3	3.9	2.43	0.57
5-LEU NH	5-LEU β	3.2	3.9	3.74	0
5-LEU NH	9-LEU α	3.1	3.8	3.9	0.1
4-VAL NH	4-VAL γ	3	3.7	2.76	0.24
4-VAL NH	4-VAL γ	3.6	4.4	4.64	0.24
9-LEU NH	9-LEU β	3.1	3.8	2.53	0.57
9-LEU NH	9-LEU β	3.3	4	3.78	0
6-LEU α	6-LEU β	2.6	4.3	2.59	0.01
9-LEU α	9-LEU β	2.6	4.1	2.53	0.07

5-LEU α	5-LEU β	2.4	3.9	2.82	0
9-LEU α	10-VAL γ	3.2	4.9	4.23	0
7-THR β	12-ALA α1	3.4	4.2	4.36	0.16
7-THR β	12-ALA β1	2	2.5	2.26	0
13-PRO α	14-ALA β	4.3	6.1	6.27	0.17
8-THR α	14-ALA α	3.1	4.8	4.42	0
7-THR α	14-ALA α	3.5	5.12	5.26	0.14
7-THR α	12-ALA β2	3	3.7	4.45	0.75
11-ALA NH	10-VAL γ	3.4	4.2	3.68	0
14-ALA NH	13-PRO γ	3.5	5.2	3.41	0.09
13-PRO α	14-ALA NH	2.9	3.6	3.51	0
14-ALA NH	13-PRO δ	2.9	3.6	3.07	0
8-THR α	14-ALA NH	3.1	3.8	4.06	0.26

Average Restraint Violation: 0.114
Average RMS RestrViolation: 0.116

Supplementary Table 7. The number of predicted core NRPs before and after filtering for the genomes of the 20 soil-dwelling Actinobacteria strains in SoilActi. The columns show the number of NRP-producing BGCs (column "#NRP-producing BGC") along with the number core NRPs generated by the canonical and non-canonical assembly lines for each genome before and after filtering by NRPminer using OrfDel option. Column "removing no ORFs" shows the number of core NRPs generated from the canonical assembly lines before and after filtering. For example, in the case of *S. albus* genome, NRPminer produces 102,852,968,758 core NRPs before filtering, while after filtering only 2,368 core NRPs are retained. Column "removing one ORF" shows the number of core NRPs generated from all non-canonical assembly lines resulting from removing A-domains encoded by one ORF on the corresponding BGC, before and after filtering with NRPminer. Column "removing two ORFs" shows this figure for non-canonical assembly lines generated by removing A-domains encoded by two ORFs. Column "total" shows the total number of core NRPs before and after filtering across all considered assembly lines for each organism. The strains corresponding to the datasets yielding the novel NRPs in SoilActi are shown in blue.

strain	#NRP-producing BGCs	#unique core NRPs before / after filtering generated by different assembly lines			
		removing no ORFs	removing one ORF	removing two ORFs	total
SCNY228	3	2,369/102,852,968,758	5,759/1,537,478,841	7,483/4023,756	15,611/104,394,471,355
albus	3	3,189/25,713,264,922	5,788/473,652,036	7,471/2237,220	16,460/2,618,9154,178
CNS654	5	1,560/21,499,085,734	3,870/87,589,011	2,331/45,216	7,761/21,586,719,961
griseoflav	7	3,235/17,916,143,265	6,431/75,146,556	2,484/45,695	12,150/17,991,335,516
hygro	5	3,753/79,748,772	12,887/27,905,444	11,964/5481,248	28,604/113,135,464
15998	3	2,436/19,088,674	8,084/49,356,874	19,156/43,902,448	29,676/112,347,996
coelicolor	3	1,191/787,524	1,693/75,438	91/819	2,975/863,781
lividan	2	1,032/262,476	2,662/178,686	1,572/31644	5,266/472,806
ghana	2	1,666/115,488	5,516/246,416	6,137/146728	13,319/508,632
kutzneria	9	4,983/47,046	9,866/73,172	5,748/53050	20,597/173,268
aa4	2	1,381/111,780	798/2,554	103/351	2,282/114,685
CNB091	3	960/29,448	603/16,976	290/3124	1,853/49,548
cattleya	4	1,300/23,068	1,475/6,165	77/225	2,852/29,458
11379	4	1,643/6,853	2,800/11,961	1,632/6,882	6,075/25,696
griseoflav	2	2,173/15,488	1,240/3,016	368/368	3,771/18,872
tu6071	4	1,674/10,638	0/0	0/0	1,674/10,638
pristin	2	864/864	279/279	0/0	1,143/1,143
afghan	0	252/252	288/288	72/72	612/612
e14	1	240/240	0/0	0/0	240/240
viridochromoges	1	36/36	0/0	0/0	36/36

Supplementary Table 8. Amino acid sequences of the 19 NRPs identified by NRPminer appearing in spectral network presented in Supplementary Figure 2.b (with the lowest p-value among the PSMs corresponding to all spectra originating from the same NRP). The known surugamide variants are shown in green. The column "predicted aa sequence" shows the sequence of corresponding NRPs as predicted by NRPminer. The "[+14]" represents addition of [+14.01Da] and "[+28]" represents addition of [+28.03Da]. Column "precursor mass" shows the precursor mass of the matched spectra and the column "p-vale" presents the p-value of the corresponding PSMs. The p-values are computed based on MCMC approach using MS-DPR[4] with 10000 simulations.

predicted aa Sequence	precursor mass	p-value
IAI---FL	558.37	9.2×10^{-16}
IAV--IFL	657.44	4.9×10^{-19}
IAI--IFL	671.45	3.1×10^{-32}
IAII-IFL	770.52	3.1×10^{-27}
IAV-KVFL	771.52	1.3×10^{-44}
IAII-IFL	784.54	3.5×10^{-20}
IAV-KIFL	785.53	8.1×10^{-47}
IAI-KIFL	799.55	6.4×10^{-43}
IAI-[+14]KIFL	813.56	5.6×10^{-50}
VAVVKVFL	856.57	4.9×10^{-45}
IAIVKIIL	864.63	4.1×10^{-55}
IAVVKVFL	870.59	8.7×10^{-73}
IAIIKIIL	878.65	1.4×10^{-27}
IAVVKIFL	884.60	2.6×10^{-59}
IAIVKIFL	898.62	3.3×10^{-67}
IAIIKIFL	912.63	6.9×10^{-65}
IAIVKIYL	914.61	3.5×10^{-43}
IAII[+14]KIFL	926.65	1.3×10^{-56}
IAII[+28]KIYL	928.63	1.9×10^{-56}

Supplementary Table 9. PSMs identified by NRPminer in the TinyEarth dataset representing the known NRP families. For each NRP family, the information about the PSM with the lowest p-value among all PSMs corresponding to the spectra representing the NRPs in that family, is listed. The column “matched genome” shows the name of the organism whose BGCs generated the putative NRP structure corresponding to the listed PSM and the column "BGC position” presents the contig and the starting and ending nucleotide position of the BGC in that contig. Columns “precursor mass” and “charge” list the precursor mass and the charge state of the matched spectra. The p-values are computed based on MCMC approach using MS-DPR[4] with 10000 simulations.

**NRP family name**	**matched genome**	**BGC position**	**p-value**	**precursor mass**	**charge**
Surfactin	*Bacillus amyloliquefaciens* sp. GZYCT-4-2	ctg1: 416695 - 482102	1.6×10^{-46}	1036.7	1
Plipastatin	*Bacillus amyloliquefaciens* sp. GZYCT-4-2	ctg1: 2727818 - 2749701	7.0×10^{-55}	731.4	2
Arthrofactin	*Pseudomonas baetica* sp. 04-6(1)	ctg1: 3,566,169 - 3,642,017	2.7×10^{-39}	1354.8	2

Supplementary Table 10. NRPminer-generated PSMs representing all known surfactins[6] and plipastatins[7,8] identified in spectra$_{\text{TinyEarth}}$ dataset. For each known NRP, the PSM with the lowest p-value among all PSMs corresponding to the spectra generated from that NRP, is listed. The columns "core NRP aa sequence" and "structure" presents the core NRP and the backbone structure of each variant identified in TinyEarth dataset. Column "precursor mass" and "charge" lists the precursor mass and the charge state of the matched spectra. The p-values are computed based on MCMC approach using MS-DPR[4] with 10000 simulations.

NRP family name	core NRP aa sequence	structure	precursor mass	p-value	charge
Surfactins	ELLVDLL	cyclic	966.5	2.5×10^{-23}	1
	ELLVDLL	cyclic	980.6	3.4×10^{-30}	1
	ELLVDLL	cyclic	994.7	4.0×10^{-35}	1
	ELLVDLL	cyclic	1008.7	2.9×10^{-45}	1
	ELLVDLL	linear	1012.7	2.1×10^{-17}	1
	ELLIDLL	cyclic	1022.7	1.5×10^{-41}	1
	ELLVDLL	linear	1026.7	3.3×10^{-19}	1
	ELLVDLL	cyclic	1029.7	8.1×10^{-20}	1
	ELLIDLL	cyclic	1036.7	1.6×10^{-46}	1
	ELLVDLL	linear	1040.7	9.0×10^{-19}	1
	ELLVDLL	cyclic	1044.7	9.2×10^{-16}	1
	ELLVDLL	cyclic	1050.7	2.1×10^{-28}	1
	ELLVDLL	linear	1054.7	6.8×10^{-16}	1
	ELLIDLL	cyclic	1057.7	7.8×10^{-24}	1
	ELLVDLL	cyclic	1064.7	2.0×10^{-41}	1
	ELLVDLL	linear	1068.7	6.4×10^{-31}	1
	ELLVDLL	cyclic	1071.7	3.9×10^{-22}	1
Plipastatins	EOYTEAPQYI	cyclic	718.4	9.6×10^{-33}	2
	EOYTEAPQYI	cyclic	724.4	2.9×10^{-30}	2
	EOYTEAPQYI	cyclic	725.4	2.5×10^{-38}	2
	EOYTEAPQYI	cyclic	731.4	7.0×10^{-55}	2
	EOYTEAPQYI	cyclic	732.4	2.3×10^{-37}	2
	EOYTEVPQYI	cyclic	739.4	3.2×10^{-49}	2
	EOYTEVPQYI	cyclic	746.4	5.5×10^{-43}	2
	EOYTEVPQYI	cyclic	753.4	6.1×10^{-42}	2
	EOYTEVPQYI	cyclic	760.4	2.6×10^{-21}	2

Supplementary Table 11. Oligonucleotides used in this study.

Oligo	Sequence	Purpose	Reference
PEB_317	TTTGGGCTAACAGGAGGCTAGCAT_ ATGAGAATACCTGAAGGTTCG	generating a fragment of *prtA* with homologous arms to pCEP-km	this study
PEB_318	TCTGCAGAGCTCGAGCATGCACAT_ CGTAATGAAACGAGTTCAGG	verification of integration of pCEP_*prtA*-km into the genome	this study
PEB_319	GACAGGGGTAATGCTAATGCC	verification of integration of pCEP_*prtA*-km into the genome	this study
VpCEP-fw	GCTATGCCATAGCATTTTTATCCAT AAG	verification of integration of pCEP_*prtA*-km into the genome	5

Supplementary References

1. He, J. & Hertweck, C. Iteration as Programmed Event during Polyketide Assembly; Molecular Analysis of the Aureothin Biosynthesis Gene Cluster. *Chemistry and Biology* **10,** 1225–1232 (2003).
2. Wilkinson, B. *et al.* Novel octaketide macrolides related to 6-deoxyerythronolide B provide evidence for iterative operation of the erythromycin polyketide synthase. *Chemistry and Biology* **7,** 111–117 (2000).
3. Röttig, M. *et al.* NRPSpredictor2 - A web server for predicting NRPS adenylation domain specificity. *Nucleic Acids Research* **39,** 362–367 (2011).
4. Mohimani, H., Kim, S. & Pevzner, P. A. A new approach to evaluating statistical significance of spectral identifications. *Journal of Proteome Research* **12,** 1560–1568 (2013).
5. Bode, E. *et al.* Promoter Activation in Δhfq Mutants as an Efficient Tool for Specialized Metabolite Production Enabling Direct Bioactivity Testing. *Angewandte Chemie* **131,** 19133–19139 (2019).
6. Sandrin, C., Peypoux, F. & Michel, G. Coproduction of surfactin and iturin A, lipopeptides with surfactant and antifungal properties, by Bacillus subtilis. *Biotechnology and Applied Biochemistry* **12,** (1990).
7. Nishikiori, T., Naganawa, H., Muraoka, Y., Aoyagi, T. & Umezawa, H. Plipastatins: New inhibitors of phospholipase A2, produced by bacillus cereus BMG302-fF67: II. structure of fatty acid residue and amino acid sequence. *The Journal of Antibiotics* **39,** 745–754 (1986).
8. Gao, L. *et al.* Plipastatin and surfactin coproduction by Bacillus subtilis pB2-L and their effects on microorganisms. *Antonie van Leeuwenhoek, International Journal of General and Molecular Microbiology* **110,** 1007–1018 (2017).
